# Variance and volatility swap valuation with nonlinear liquidity-feedback stochastic volatility and regime switching

**DOI:** 10.1371/journal.pone.0355147

**Published:** 2026-10-27

**Authors:** Jing Fu, Ke Wang

**Affiliations:** 1 School of Mathematics and Statistics, Zhaotong University, Zhaoyang, Zhaotong, Yunnan, P.R. China; 2 School of Science, Civil Aviation Flight University of China (CAFUC), Guanghan, Sichuan, P.R. China; Mercer University, UNITED STATES OF AMERICA

## Abstract

This paper values discretely sampled variance swaps and absolute-return-based volatility swaps under regime-switching stochastic volatility and stochastic liquidity. Extending an existing stochastic-liquidity model, a quadratic liquidity-stress term shifts the variance-factor mean-reversion target. An exponential-quadratic forward transform yields pricing formulas through coupled Riccati equations and a finite-state Markov backward equation. Explicit finite-horizon sufficient conditions establish martingale and transform admissibility. Recomputed numerical experiments assess simulation agreement, integration accuracy and nested liquidity channels. Under the uncalibrated baseline parameterization, delayed variance feedback contributes more than the direct return-liquidity channel, while their cross-channel interaction is small. These conclusions concern the stated illustrative parameter settings and the specified absolute-return contract, whose payoff differs from the square root of realized variance.

## Introduction

Volatility risk is one of the central sources of uncertainty in financial markets. Since asset volatility is not directly traded and usually evolves randomly over time, variance swaps and volatility swaps provide useful instruments for transferring, hedging and acquiring exposure to realized variation. Their payoffs are linked to specified realized-variation measures over a prescribed monitoring period. This paper retains the absolute-return-based volatility measure used in the predecessor model and distinguishes it from the square root of realized variance. Consequently, their valuation depends not only on the dynamics of the underlying asset price, but also on the stochastic behavior of volatility, the prevailing economic state and the monitoring convention used in the contract.

A key feature of practical volatility derivatives is discrete monitoring. Continuous sampling is mathematically convenient and often leads to tractable limiting representations, but market contracts are typically monitored on daily, weekly or monthly grids. Under discrete simple-return monitoring, finite-period returns and finite-period compounding enter the payoff directly. This feature is especially relevant for variance swaps, whose payoff preserves squared-return amplification, and for volatility swaps, whose payoff in this paper uses an absolute-return aggregation. Pricing formulas that retain the discrete monitoring structure are therefore important for both valuation and numerical implementation.

Stochastic volatility provides a natural starting point for modeling realized variation. Volatility dynamics in financial markets are also affected by changing economic conditions and market liquidity. Regime switching captures the effect of economic states on volatility levels, while stochastic market liquidity represents market-wide liquidity stress and its impact on trading prices. These considerations motivate a hybrid stochastic framework in which regime-switching economic states, stochastic variance and liquidity stress jointly affect the distribution of monitored returns.

This paper studies discretely sampled variance and volatility swaps in such a hybrid framework. The liquidity state is represented by a signed liquidity-stress factor $\lambda_{t}$, and its square $\lambda_{t}^{2}$ is interpreted as liquidity-stress intensity. The model separates two liquidity transmission channels. The direct return-liquidity channel affects instantaneous asset returns through the liquidity loading in the price dynamics. The delayed volatility-feedback channel enters the variance drift through the effective variance mean-reversion target ${\tilde{\delta }}_{X_{t}}+\chi \lambda_{t}^{2}$. The feedback coefficient quantifies the nonlinear transmission from liquidity stress to future variance while preserving a tractable transform representation.

This paper is related to several strands of the financial mathematics literature. The martingale pricing framework follows the continuous-time no-arbitrage foundation of Harrison and Pliska [1]. Stochastic volatility and transform-based valuation are closely connected to the Heston model [2] and affine transform methods [3,4]. Fourier-based computation further provides an efficient tool for derivative valuation under latent stochastic state variables [5]. These methods form the analytical foundation for characteristic-function-based pricing.

Variance and volatility swap valuation under stochastic volatility and discrete monitoring has been widely studied. Research on realized-variation claims derives analytical or semi-analytical representations for variance swaps, volatility swaps and related payoffs [6–10]. These contributions also highlight the importance of the monitoring convention, since discretely monitored simple returns need not coincide with continuous quadratic variation over finite horizons.

Related extensions address non-Markovian volatility. Horvath et al. [11] develop simulation and approximation methods for volatility options with Gaussian and modulated Volterra volatility. Salmon and SenGupta [12] combine fractional long memory (*H* > 1/2) and jumps in a fractional Barndorff–Nielsen–Shephard model; this should be distinguished from the low-Hurst rough-volatility setting. Wang and Xia [13] obtain transforms of average forward variance for power-type derivatives under rough volatility with jumps. These works address memory and nonlinear volatility payoffs, whereas the present finite-dimensional Markov model isolates liquidity feedback. Combining these features would require new state or transform constructions.

Regime switching provides a flexible way to represent changing economic conditions. Financial markets move across different macroeconomic or market states, and long-run volatility levels may depend on the prevailing state. Markov-modulated models and hidden Markov formulations offer a natural probabilistic framework for such state-dependent dynamics [14–18]. In volatility derivative valuation, regime switching makes it possible to capture state-dependent shifts in the distribution of realized variation.

Stochastic market liquidity is another relevant source of risk for derivative valuation. Liquidity conditions affect transaction prices, asset discounts and the compensation required for holding liquidity-sensitive positions. Liquidity-adjusted pricing models and subsequent studies on options, variance swaps and volatility swaps with stochastic liquidity show that market liquidity can have a significant impact on derivative prices [19–23]. Building on this line of research, the proposed framework complements stochastic-liquidity valuation models by allowing liquidity-stress intensity to shift the future variance mean-reversion target. The model enriches the liquidity-volatility transmission mechanism through a nonlinear stochastic feedback channel.

The relationship to He et al. [23] is specific. The no-feedback risk-neutral dynamics, regime-dependent variance target, signed OU liquidity factor, simple-return contract definitions and two-stage forward-transform architecture are inherited from that framework. The incremental model term is $\chi \lambda^{2}$ in the variance target; it induces the two forcing terms $\epsilon \chi \xi_{2}$ and $\epsilon \chi \xi_{6}$ and permits a nested decomposition of direct and delayed liquidity effects. The shared derivation framework comprises the exponential-quadratic ansatz, the no-feedback coefficient equations and two-stage conditioning; the additional $\epsilon \chi \xi_{2}$ and $\epsilon \chi \xi_{6}$ forcings arise by substituting the feedback drift. The authors confirm that derivation materials were not copied or adapted from that article and that its code was not inherited or modified; methodological inheritance is nevertheless explicitly acknowledged. The revised numerical comparison reports both agreement and the residual discrepancy with its published graph. No figures or tabulated results from that paper are reused as new results here; the attributed graph readings in the external comparison serve only as comparison data.

The tractability mechanism belongs to the established quadratic and linear–quadratic transform literature [24–26]. The contribution is this particular liquidity-feedback specification and its analysis, not a new general transform class. The word “nonlinear” refers to the dependence on the signed factor in the original state variables; the augmented state includes $\lambda^{2}$. The forward-transform and discrete-monitoring methods also build on Elliott and Lian [17], Zheng and Kwok [10], Zhu and Lian [9], Broadie and Jain [27].

The remainder of this paper is organized as follows. section “Model” presents the regime-switching stochastic volatility model with stochastic liquidity and nonlinear feedback. section “Pricing formulas” derives the semi-analytical forward characteristic function and the pricing formulas for discretely monitored swaps. section “Numerical analysis” presents the numerical analysis, including validation, convergence checks, nested benchmark decompositions and nonlinear feedback diagnostics. section “Conclusion” concludes the paper.

## Model

In this section, we construct the hybrid modeling framework that underlies the subsequent valuation analysis. We first introduce a continuous-time Markov chain to govern regime-switching economic states, then specify the liquidity-adjusted price dynamics and the stochastic variance equation with nonlinear feedback, and finally derive the log-price dynamics that serves as the basis for the characteristic-function-based pricing approach.

### Regime-switching economic states

We introduce a continuous-time Markov chain $\{X_{t}{\}}_{t\geq 0}$ taking values in the unit vectors $e_{1},...,e_{N}$ of ${\mathbb{R}}^{N}$, each corresponding to a distinct economic regime with its own variance parameters. Under the risk-neutral measure ℚ, the regime transitions are governed by the row generator


$$\begin{array}{c} G^{\mathbb{Q}}=[g_{ij}{]}_{i,j=1}^{N},\;g_{ij}\geq 0\;(i\text{≠}j),\;\sum_{j=1}^{N}g_{ij}=0, \end{array}$$ (1)

where $g_{ij}$ are the instantaneous transition intensities and the diagonal entries ensure zero row sums. The row probability vector $p(t)=\mathbb{Q}(X_{t}=e_{i})$ then satisfies $p^{\prime }(t)=p(t)G^{\mathbb{Q}}$.

The regime-switching mechanism enters through the long-run variance target. For a column vector ${\tilde{\delta }}^{*}=({\tilde{\delta }}_{1},...,{\tilde{\delta }}_{N}{)}^{⊤}$, we define


$$\begin{array}{c} {\tilde{\delta }}_{X_{t}}=\langle {\tilde{\delta }}^{*},X_{t}\rangle , \end{array}$$ (2)

so that the target varies with the economic regime.

**Assumption 1** (Markov chain and Brownian correlation structure). *The process X is independent of the Brownian vector* $\left(W^{1},W^{2},W^{3},W^{4}\right)$*. The variance process nevertheless depends on X through its drift. The Brownian motions* $\left(W^{1},W^{2},W^{3},W^{4}\right)$ *satisfy*


$$\begin{array}{c} \;dW_{t}^{1}\;dW_{t}^{2}=\rho_{1}\;dt,\;\;dW_{t}^{3}\;dW_{t}^{4}=\rho_{2}\;dt, \end{array}$$ (3)

*with all other instantaneous covariations zero.*

The correlation $\rho_{1}$ captures the leverage effect, while $\rho_{2}$ allows for asymmetric liquidity dynamics.

### Liquidity-adjusted price and variance dynamics

With the regime-switching structure in place, we specify the dynamics of the liquidity-adjusted asset price and variance process. Under ℚ,


$$\begin{array}{c} \frac{\;dS_{t}}{S_{t}}=r\;dt+\sqrt{\eta_{t}}\;dW_{t}^{1}+\sigma \lambda_{t}\;dW_{t}^{3}. \end{array}$$ (4)

The base variance-factor process $(\eta_{t}{)}_{t\geq 0}$ and signed liquidity-stress process $(\lambda_{t}{)}_{t\geq 0}$ follow


$$\begin{array}{cc} \;d\eta_{t} & =\epsilon \left({\tilde{\delta }}_{X_{t}}+\chi \lambda_{t}^{2}-\eta_{t}\right)dt+\omega \sqrt{\eta_{t}}\;dW_{t}^{2}, \end{array}$$ (5)

$$\begin{array}{cc} \;d\lambda_{t} & =\tilde{\gamma }\left(\tilde{\alpha }-\lambda_{t}\right)dt+\beta \;dW_{t}^{4}. \end{array}$$ (6)

The parameters are interpreted as follows: *r* is the risk-free rate; σ controls the direct return-liquidity channel; ϵ is the mean-reversion speed of variance; ω is the volatility of variance (vol-of-vol); χ is the nonlinear liquidity-feedback coefficient; $\tilde{\gamma }$ and $\tilde{\alpha }$ are the mean-reversion speed and long-run mean of the liquidity factor, respectively; β is the volatility of the liquidity factor; and the regime-dependent target ${\tilde{\delta }}_{X_{t}}$ is defined in (2). The correlations $\rho_{1}$ and $\rho_{2}$ are specified in Assumption 1. Here χ≥0 shifts the variance target upward with liquidity-stress intensity. The direct return-liquidity channel is controlled by σ, while the delayed feedback channel is governed by χ, a key feature examined numerically in section “Numerical analysis”. Under the stated zero correlation between *W*^1^ and *W*^3^, the instantaneous return variance rate is $v_{t}=\eta_{t}+\sigma^{2}\lambda_{t}^{2}$, and its square root is the instantaneous return volatility. Thus $\eta_{t}$ alone is not the variance of liquidity-adjusted returns.

#### Scale convention and model restrictions.

For any *c* > 0, the transformation


$$\begin{array}{c} (\lambda ,\tilde{\alpha },\beta ,\lambda_{0})\mapsto c(\lambda ,\tilde{\alpha },\beta ,\lambda_{0}),\;\sigma \mapsto \sigma /c,\;\chi \mapsto \chi /c^{2} \end{array}$$

leaves return dynamics and feedback unchanged; $\tilde{\gamma }$ is unchanged. To remove this free scale in the present family, we fix $\lambda_{0}=0.20$ as the liquidity unit and do not estimate it. For positive initial signed stress, any other positive initial scale can be mapped to this convention. This is a normalization, not evidence that initial stress is empirically known. All sensitivity experiments retain this fixed value, so variations in β or χ change the economic scenario rather than merely its units. A zero initial factor would require another scale convention, such as fixing β; negative initial stress requires a separately specified sign convention. The chosen normalization remains well defined at χ=0 and σ=0.

The signed OU specification permits positive and negative stress states with a Gaussian transition law; both enter the target through their squared magnitude. A CIR intensity ℓ≥0, with return loading $\sigma \sqrt{\ell }$ and feedback χℓ, is a parsimonious affine alternative. We do not claim an empirical advantage for the OU choice. The assumption $dW^{2}dW^{4}=0$ is a tractability restriction: nonzero correlation would introduce a mixed derivative proportional to $\sqrt{\eta }\;f_{\eta \lambda }$ and, under the current ansatz, terms outside the retained linear–quadratic basis. The weak baseline sensitivity to $\rho_{2}$ also limits the empirical case for this additional dependence channel.

**Assumption 2** (Parameter admissibility and variance positivity). *Parameters satisfy*


$$\begin{array}{c} \epsilon ,\omega ,\tilde{\gamma },\beta >0,\;\sigma ,\chi \geq 0,\;\eta_{0}>0,\;{\tilde{\delta }}_{i}>0\;(i=1,...,N),\;\rho_{1},\rho_{2}\in (-1,1), \end{array}$$

*and the sufficient Feller-type condition*


$$\begin{array}{c} 2\epsilon \min_{i}{\tilde{\delta }}_{i}>\omega^{2}. \end{array}$$ (7)

**Assumption 3** (Checkable martingale sufficient condition). *For the chosen finite maturity T, the parameter inequalities (A.3) in Novikov bound hold. They imply*


$$\begin{array}{c} {𝔼}^{\mathbb{Q}}\;\left[\exp\left\{\frac{1}{2}\int_{0}^{T}(\eta_{s}+\sigma^{2}\lambda_{s}^{2},ds\right\}\right]<\infty . \end{array}$$ (8)

*The condition is sufficient, not necessary, and is checked for the maturity in use rather than assumed for every maturity simultaneously.*

**Lemma 1** (Existence and strict positivity of the variance factor). *Let* $\underset{―}{\delta }=\min_{i}{\tilde{\delta }}_{i}$*. Under Assumption 2, the variance process in (5) admits a nonnegative weak solution and remains strictly positive on finite horizons.*

*Proof.* Since $(\lambda_{t}{)}_{t\geq 0}$ is an Ornstein-Uhlenbeck process with globally Lipschitz drift, it has a unique non-explosive strong solution. As χ≥0 and $\lambda_{t}^{2}\geq 0$,


$$\begin{array}{c} {\tilde{\delta }}_{X_{t}}+\chi \lambda_{t}^{2}\geq \underset{―}{\delta }. \end{array}$$ (9)

Consider the comparison CIR process driven by the same *W*^2^:


$$\begin{array}{c} \;d{\bar{\eta }}_{t}=\epsilon (\underset{―}{\delta }-{\bar{\eta }}_{t},dt+\omega \sqrt{{\bar{\eta }}_{t}}\;dW_{t}^{2},\;{\bar{\eta }}_{0}=\eta_{0}. \end{array}$$ (10)

For x≥0, the drift of $\eta_{t}$ satisfies


$$\begin{array}{c} \epsilon ({\tilde{\delta }}_{X_{t}}+\chi \lambda_{t}^{2}-x)\geq \epsilon (\underset{―}{\delta }-x), \end{array}$$ (11)

with the same diffusion coefficient $\omega \sqrt{x}$. For a precise comparison result, Theorem 1.1 and its localized version in Remark 1.1 of Ikeda and Watanabe [28] allow adapted drift processes bounded by deterministic comparison drifts. Here $|\omega \sqrt{x^{+}}-\omega \sqrt{y^{+}}|\leq \omega \sqrt{\left|x-y\right|}$ and the comparison CIR equation is pathwise unique. The random drift is locally integrable after localization because the OU paths are continuous and the regime targets are bounded. Local existence with the square-root diffusion follows by approximation, and localization plus the finite first moments of $\int_{0}^{T}\lambda_{s}^{2}ds$ precludes finite-horizon explosion. The comparison theorem therefore applies; hence $\eta_{t}\geq {\bar{\eta }}_{t}$. Under $2\epsilon \underset{―}{\delta }>\omega^{2}$, the comparison CIR process stays strictly positive, so, almost surely, $\eta_{t}>0$ for all t≥0. □

**Proposition 1** (Risk-neutral admissibility and true martingale property). *Under Assumptions 1–3,* $\eta_{t}>0$ *on finite horizons and* $(e^{-rt}S_{t}{)}_{0\leq t\leq T}$ *is a true martingale under* ℚ *on the chosen admissible horizon.*

*Proof.* From (4),


$$\begin{array}{c} e^{-rt}S_{t}=S_{0}\;ℰ(M{)}_{t}, \end{array}$$ (12)

with ℰ the Doléans-Dade exponential and


$$\begin{array}{c} M_{t}=\int_{0}^{t}\sqrt{\eta_{s}}\;dW_{s}^{1}+\int_{0}^{t}\sigma \lambda_{s}\;dW_{s}^{3}. \end{array}$$ (13)

The quadratic variation is


$$\begin{array}{c} \langle M\rangle_{t}=\int_{0}^{t}(\eta_{s}+\sigma^{2}\lambda_{s}^{2},ds. \end{array}$$ (14)

By the exponential supersolution bound in Novikov bound and Assumption 3, Novikov’s criterion holds, so ℰ(M) is a true martingale.

### Log-price dynamics

Having established the stochastic dynamics and their admissibility, we derive the log-price process, which underpins the characteristic-function approach in section “Pricing formulas”. Let $z_{t}=\ln S_{t}$. Applying Itô’s formula to (4) yields


$$\begin{array}{c} \;dz_{t}=\left[r-\frac{1}{2}\left(\eta_{t}+\sigma^{2}\lambda_{t}^{2}\right)\right]\;dt+\sqrt{\eta_{t}}\;dW_{t}^{1}+\sigma \lambda_{t}\;dW_{t}^{3}. \end{array}$$ (15)

Eq (15) reveals the combined effect of both liquidity channels. The direct channel contributes to the instantaneous return variance via $\sigma^{2}\lambda_{t}^{2}$ and the diffusion term $\sigma \lambda_{t}\;dW_{t}^{3}$. The delayed feedback channel, though not directly visible in the log-price equation, influences future returns through its effect on $\eta_{t}$, which in turn feeds back into the log-price dynamics. This two-tier structure–-liquidity affecting variance, and variance affecting returns–-generates the interaction patterns explored in the pricing formulas below.

Eq (15) serves as the basis for the transform derivation. In section “Pricing formulas”, we construct the forward characteristic function of log-return increments using the exponential-quadratic structure, and employ it to obtain semi-analytical pricing formulas for discretely monitored variance and volatility swaps.

## Pricing formulas

This section derives pricing formulas for discretely monitored variance and volatility swaps within the hybrid framework of section “Model”. We first construct the forward characteristic function of log-return increments, then express the variance swap strike in terms of this function at specific imaginary arguments, and finally obtain the volatility swap strike via a Fourier identity for the absolute value. Throughout, we assume Assumption 4 holds.

### Forward characteristic function

The forward characteristic function provides a compact representation of the conditional distribution of log-return increments. For $u_{1}\leq u_{2}<u_{3}$, define


$$\begin{array}{c} K_{\chi }(\psi ;\eta_{u_{1}},\lambda_{u_{1}},X_{u_{1}},u_{1},u_{2},u_{3})={𝔼}^{\mathbb{Q}}\left[\exp\{j\psi (z_{u_{3}}-z_{u_{2}}\}|{ℱ}_{u_{1}}\right], \end{array}$$ (16)

where *z* is the log-price process with dynamics (15), $j=\sqrt{-1}$, $\tau_{2}=u_{3}-u_{2}$ and $\tau_{1}=u_{2}-u_{1}$.

**Assumption 4** (Checkable transform sufficient condition). *For some h > 0, the parameter inequalities (A.4) hold on the entire pricing horizon. They imply finite return moments for real exponential loadings in* [−h,2+h] *and hence existence of the complex transform on the associated strip for every real Fourier frequency. Both stages of the forward transform are covered. In the numerical experiments h = 0.1; parameters outside this sufficient region require additional analysis and are not automatically inadmissible.*

**Proposition 2** (Forward characteristic function). *Under Assumptions 1–4, the forward characteristic function is given as follows. Let* $\xi_{2},\xi_{3},\xi_{4},{\bar{\xi }}_{1}$ *solve, with zero initial values,*


$$\begin{array}{cc} \frac{\;d\xi_{4}}{\;ds} & =2\beta^{2}\xi_{4}^{2}+\left(2j\beta \sigma \rho_{2}\psi -2\tilde{\gamma }\right)\xi_{4}-\frac{1}{2}\sigma^{2}(j\psi +\psi^{2})+\epsilon \chi \xi_{2}, \end{array}$$ (17)

$$\begin{array}{cc} \frac{\;d\xi_{3}}{\;ds} & =\left(2\beta^{2}\xi_{4}-\tilde{\gamma }+j\beta \sigma \rho_{2}\psi \right)\xi_{3}+2\tilde{\gamma }\tilde{\alpha }\xi_{4}, \end{array}$$ (18)

$$\begin{array}{cc} \frac{\;d\xi_{2}}{\;ds} & =\frac{1}{2}\omega^{2}\xi_{2}^{2}+\left(j\omega \rho_{1}\psi -\epsilon \right)\xi_{2}-\frac{1}{2}(j\psi +\psi^{2}), \end{array}$$ (19)

$$\begin{array}{cc} \frac{\;d{\bar{\xi }}_{1}}{\;ds} & =\frac{1}{2}\beta^{2}\xi_{3}^{2}+\tilde{\gamma }\tilde{\alpha }\xi_{3}+\beta^{2}\xi_{4}+jr\psi \end{array}$$ (20)

*on* $s\in [0,\tau_{2}]$*. Let* $\xi_{6},\xi_{7},\xi_{8},{\bar{\xi }}_{5}$ *solve*


$$\begin{array}{cc} \frac{\;d\xi_{8}}{\;ds} & =2\beta^{2}\xi_{8}^{2}-2\tilde{\gamma }\xi_{8}+\epsilon \chi \xi_{6}, \end{array}$$ (21)

$$\begin{array}{cc} \frac{\;d\xi_{7}}{\;ds} & =\left(2\beta^{2}\xi_{8}-\tilde{\gamma }\right)\xi_{7}+2\tilde{\gamma }\tilde{\alpha }\xi_{8}, \end{array}$$ (22)

$$\begin{array}{cc} \frac{\;d\xi_{6}}{\;ds} & =\frac{1}{2}\omega^{2}\xi_{6}^{2}-\epsilon \xi_{6}, \end{array}$$ (23)

$$\begin{array}{cc} \frac{\;d{\bar{\xi }}_{5}}{\;ds} & =\frac{1}{2}\beta^{2}\xi_{7}^{2}+\tilde{\gamma }\tilde{\alpha }\xi_{7}+\beta^{2}\xi_{8} \end{array}$$ (24)

*on* $s\in [0,\tau_{1}]$*, with*


$$\begin{array}{c} \xi_{6}(0)=\xi_{2}(\tau_{2}),\;\xi_{7}(0)=\xi_{3}(\tau_{2}),\;\xi_{8}(0)=\xi_{4}(\tau_{2}),\;{\bar{\xi }}_{5}(0)=0. \end{array}$$ (25)

*Define*


$$\begin{array}{c} b_{\psi }(t)=\left\{ \begin{array}{cc} \epsilon \xi_{6}(u_{2}-ttilde\delta^{*}, & u_{1}\leq t\leq u_{2},\\ \epsilon \xi_{2}(u_{3}-t){\tilde{\delta }}^{*}, & u_{2}<t\leq u_{3}, \end{array}\right. \end{array}$$ (26)

*and let* $F_{\psi }(t)$ *solve*


$$\begin{array}{c} \frac{\;dF_{\psi }(t)}{\;dt}=-\left[G^{\mathbb{Q}}+\mathrm{diag}(b_{\psi }(t))\right]F_{\psi }(t),\;F_{\psi }(u_{3})=1_{N}. \end{array}$$ (27)

*If* $X_{u_{1}}=e_{i}$*, set* $M_{\psi }(u_{1})=F_{\psi ,i}(u_{1})$*. Then*


$$\begin{array}{c} K_{\chi }(\psi )=\exp\left[{\bar{\xi }}_{1}(\tau_{2})+{\bar{\xi }}_{5}(\tau_{1})+\xi_{6}(\tau_{1}eta_{u_{1}}+\xi_{7}(\tau_{1})\lambda_{u_{1}}+\xi_{8}(\tau_{1})\lambda_{u_{1}}^{2}\right]M_{\psi }(u_{1}). \end{array}$$ (28)

The derivation uses *s* as the remaining time to the relevant terminal date. With this convention, the backward Kolmogorov equation $\partial_{t}f+Lf=0$ is written as $\partial_{s}f=Lf$, which explains the sign convention in the Riccati-type deterministic ODE systems. For a fixed regime state with variance target δ, the diffusion generator acting on a smooth test function f=f(z,η,λ) is


$$\begin{array}{c} \begin{array}{cc} ℒf= & \left(r-\frac{1}{2}(\eta +\sigma^{2}\lambda^{2})\right)f_{z}+\epsilon (\delta +\chi \lambda^{2}-\eta )f_{\eta }+\tilde{\gamma }(\tilde{\alpha }-\lambda )f_{\lambda }\\ & +\frac{1}{2}(\eta +\sigma^{2}\lambda^{2})f_{zz}+\frac{1}{2}\omega^{2}\eta f_{\eta \eta }+\frac{1}{2}\beta^{2}f_{\lambda \lambda }+\rho_{1}\omega \eta f_{z\eta }+\rho_{2}\sigma \beta \lambda f_{z\lambda }. \end{array} \end{array}$$

Over the log-return interval $\left[u_{2},u_{3}\right]$, the exponential-quadratic form is


$$\begin{array}{c} f(s,z,\eta ,\lambda )=\exp\{A(s)+B(seta+C(s)\lambda +D(s)\lambda^{2}+j\psi z\}. \end{array}$$

Substitution into $\partial_{s}f=Lf$ and matching the coefficients of η, λ, $\lambda^{2}$ and the constant term gives the equations for $\xi_{2}$, $\xi_{3}$, $\xi_{4}$ and ${\bar{\xi }}_{1}$, respectively. The nonlinear feedback contribution enters the $\lambda^{2}$ coefficient through


$$\begin{array}{c} \epsilon \chi \lambda^{2}f_{\eta }=\epsilon \chi B(s)\lambda^{2}f, \end{array}$$

which produces the $\epsilon \chi \xi_{2}$ term in the first-stage equation for $\xi_{4}$.

Over the preceding interval $\left[u_{1},u_{2}\right]$, the future log-return increment has not yet started, so the second-stage ansatz has no *z*-loading:


$$\begin{array}{c} g(s,\eta ,\lambda )=\exp\{A(s)+B(s)\eta +C(s)\lambda +D(s)\lambda^{2}\}. \end{array}$$

Consequently there is no jψ term in this stage. The same coefficient matching gives the second Riccati system, and the nonlinear feedback source becomes $\epsilon \chi \xi_{6}$, which enters the equation for $\xi_{8}$.

The regime-dependent contribution is not absorbed into the scalar coefficient *A*(*s*). The term


$$\begin{array}{c} \epsilon {\tilde{\delta }}_{X_{t}}B(s) \end{array}$$

is a Markov-chain additive functional, so the regime component is evaluated through the finite-dimensional backward equation


$$\begin{array}{c} F_{\psi }^{\prime }(t)=-\left(G^{Q}+\mathrm{diag}(b_{\psi }(t))\right)F_{\psi }(t), \end{array}$$

rather than by an ordinary matrix exponential.

The Riccati equations are integrated in the remaining-time variable in two stages of lengths $\tau_{2}$ and $\tau_{1}$, with the stated terminal-to-initial chaining. Verification and conditioning on the chain verifies the candidate by localization and exponential bounds, and proves the conditioning-on-the-chain-path step. The Markov-chain component $M_{\psi }(u_{1})$ accounts for regime transitions over the entire horizon.

**Remark 1** (Role of the nonlinear feedback). *The feedback enters through* $\epsilon \chi \xi_{2}$ *in (17) and* $\epsilon \chi \xi_{6}$ *in (21), direct consequences of the quadratic variance target in (5). With* χ=0*, the system reduces to the stochastic-liquidity and regime-switching variance backbone of He et al. [*23*]; the return-liquidity channel remains when* σ≠0*. Setting both* χ=0 *and* σ=0 *gives the regime-switching Heston return model.*

**Remark 2** (Markov-chain evaluation). *Under the row-generator convention, the Markov term is evaluated by the backward ODE (27); a single matrix exponential is not used because* $b_{\psi }(t)$ *varies with time through the Riccati solutions.*

**Remark 3** (Transform consistency). *When* ψ=0*, all coefficients vanish, so* $K_{\chi }(0)=1$*. When* ψ=−j*,* $j\psi +\psi^{2}=0$ *and*


$$\begin{array}{c} K_{\chi }(-j;\eta_{u_{1}},\lambda_{u_{1}},X_{u_{1}},u_{1},u_{2},u_{3})=\exp(r(u_{3}-u_{2})). \end{array}$$ (29)

*This identity provides a useful numerical consistency check*.

The identities $K_{\chi }(0)=1$ and $K_{\chi }(-j)=e^{r(u_{3}-u_{2})}$ are implementation sanity checks, not tests of true-martingale admissibility. At these arguments the Riccati source terms vanish algebraically, while $G^{\mathbb{Q}}1_{N}=0$; a formal implementation can therefore satisfy the identities even without a valid probabilistic representation. Martingale and moment admissibility instead follow from the independently checked conditions in Appendix A. Explicit finite-horizon bounds and transform verification.

We now apply the forward characteristic function derived in the previous subsection to obtain the fair strike of a discretely sampled variance swap. Let the monitoring dates be equally spaced over the contract lifetime *T*, i.e., $t_{n}=nT/N$ for n=0,1,...,N, where *N* is the total number of observations. We set *m* = 100 to convert decimal returns into percentage quotation units; annualization is supplied by the time normalization 1/*T*.

The realized variance over the monitoring period, based on simple returns, is defined as


$$\begin{array}{c} RV_{A}=\frac{m^{2}}{T}\sum_{n=1}^{N}{\left(\frac{S_{t_{n}}-S_{t_{n-1}}}{S_{t_{n-1}}}\right)}^{2}. \end{array}$$ (30)

This is the simple-return contract convention adopted here and in the comparison with reference [23]; it is not a claim that all traded variance swaps use this convention rather than log returns.

The fair strike $P_{A}^{\chi }$ of a variance swap is the fixed rate that makes the initial value of the swap zero. Under the risk-neutral measure ℚ, this is equivalent to setting


$$\begin{array}{c} P_{A}^{\chi }={𝔼}^{\mathbb{Q}}[RV_{A}], \end{array}$$ (31)

since the floating leg pays the realized variance and the fixed leg pays the strike. Thus, the pricing problem reduces to computing the risk-neutral expectation of the sum of squared simple returns.

For each monitoring interval $\left[t_{n-1},t_{n}\right]$, write the simple return as


$$\begin{array}{c} \frac{S_{t_{n}}-S_{t_{n-1}}}{S_{t_{n-1}}}=e^{\Delta z_{n}}-1, \end{array}$$ (32)

where $\Delta z_{n}=z_{t_{n}}-z_{t_{n-1}}$ is the log-return over the interval. Squaring yields


$$\begin{array}{c} {\left(e^{\Delta z_{n}}-1\right)}^{2}=e^{2\Delta z_{n}}-2e^{\Delta z_{n}}+1. \end{array}$$ (33)

Taking expectations under ℚ and conditioning on the information at time $t_{n-1}$, we obtain


$$\begin{array}{c} {𝔼}^{\mathbb{Q}}\left[{\left(e^{\Delta z_{n}}-1\right)}^{2}|{ℱ}_{t_{n-1}}\right]=K_{\chi }(-2j;\cdot )-2K_{\chi }(-j;\cdot )+1, \end{array}$$ (34)

where the characteristic function $K_{\chi }(\psi ;\cdot )$ is evaluated at ψ=−2j and ψ=−j, respectively, and the state variables are taken at time $t_{n-1}$. The term 1 arises from the expectation of the constant, and the intermediate step uses the tower property of conditional expectations. Note that the arguments −2j and −j give the second and first moments of the gross return $e^{\Delta z_{n}}$ under the risk-neutral measure.

Since the initial state variables $\eta_{0},\lambda_{0},X_{0}$ are known at time 0, the expectation over each monitoring interval is obtained by applying the Markov property and the law of total expectation. The forward characteristic function in (16) gives the conditional expectation of the log-return increment over $\left[t_{n-1},t_{n}\right]$ given the state at $t_{n-1}$. By iterating this conditioning, the unconditional expectation reduces to substituting the initial state variables into the characteristic function for each interval with time lags $\left(0,t_{n-1},t_{n}\right)$. This yields the following corollary.

**Corollary** 1 (Discretely sampled variance swap strike). *Under the admissibility conditions of Proposition 2,*


$$\begin{array}{c} P_{A}^{\chi }=\frac{m^{2}}{T}\sum_{n=1}^{N}\left[K_{\chi }(-2j;\eta_{0},\lambda_{0},X_{0},0,t_{n-1},t_{n})-2K_{\chi }(-j;\eta_{0},\lambda_{0},X_{0},0,t_{n-1},t_{n})+1\right]. \end{array}$$ (35)

This semi-analytical formula preserves discrete monitoring. It depends on χ implicitly via the characteristic function, and reduces to the no-feedback stochastic-liquidity result when χ=0.

### Volatility swap

We retain the absolute-return-based volatility contract of He et al. [23], with a non-polynomial single-period payoff that can be valued through a Fourier identity. Under the same grid $t_{n}=nT/N$ and quotation factor *m*, its realized measure is


$$\begin{array}{c} RV_{O}=m\sqrt{\frac{\pi }{2NT}}\sum_{n=1}^{N}\left|\frac{S_{t_{n}}-S_{t_{n-1}}}{S_{t_{n-1}}}\right|. \end{array}$$ (36)

This is not $\sqrt{RV_{A}}$. If independent benchmark returns were *N*(0,*vT*/*N*) with constant *v*, then $𝔼|R_{n}|=\sqrt{2/\pi }\sqrt{vT/N}$ and the displayed scaling would give $𝔼[RV_{O}]=m\sqrt{v}$. This benchmark only explains the normalization; neither zero-mean normal simple returns nor independence of monitored returns is assumed in the proposed model. In general $𝔼[RV_{O}]$, $𝔼[\sqrt{RV_{A}}]$ and $\sqrt{𝔼[RV_{A}]}$ are distinct. Throughout this article “volatility swap” refers to this explicitly specified absolute-return-based contract, not the conventional square-root-of-realized-variance payoff. Its fair strike is $P_{O}^{\chi }={𝔼}^{\mathbb{Q}}[RV_{O}]$.

For each interval, let $Y=\Delta z_{n}=z_{t_{n}}-z_{t_{n-1}}$. Under the transform admissibility and integrability conditions used in Proposition 2, the absolute simple-return expectation is evaluated through the expectation-level Fourier identity


$$\begin{array}{c} {𝔼}^{\mathbb{Q}}\left[\left|e^{Y}-1\right|\right]=\frac{2}{\pi }\int_{0}^{\infty }ℜ\left[\frac{{𝔼}^{\mathbb{Q}}(e^{(1+j\psi )Y})-{𝔼}^{\mathbb{Q}}(e^{j\psi Y})}{j\psi }\right]\;d\psi . \end{array}$$

In the present notation,


$$\begin{array}{c} {𝔼}^{\mathbb{Q}}(e^{(1+j\psi )\Delta z_{n}})=K_{\chi }(\psi -j;\cdot ),\;{𝔼}^{\mathbb{Q}}(e^{j\psi \Delta z_{n}})=K_{\chi }(\psi ;\cdot ), \end{array}$$

where $K_{\chi }$ is evaluated at the time-zero state and time arguments $\left(0,t_{n-1},t_{n}\right)$. Summing over *n* and incorporating the prefactor yields the following.

**Corollary 2** (Discretely sampled volatility swap strike). *Under the admissibility conditions of Proposition 2, the fair volatility swap strike is*


$$\begin{array}{cc} P_{O}^{\chi } & =m\sqrt{\frac{2}{\pi NT}}\sum_{n=1}^{N}\int_{0}^{\infty }ℜ\left[\frac{{𝒦}_{n}^{\left(1\right)}(\psi )-{𝒦}_{n}^{\left(0\right)}(\psi )}{j\psi }\right]\;d\psi ,\\ {𝒦}_{n}^{\left(1\right)}(\psi ) & =K_{\chi }(\psi -j;\eta_{0},\lambda_{0},X_{0},0,t_{n-1},t_{n}),\\ {𝒦}_{n}^{\left(0\right)}(\psi ) & =K_{\chi }(\psi ;\eta_{0},\lambda_{0},X_{0},0,t_{n-1},t_{n}). \end{array}$$ (37)

The sign-function Fourier representation is inherited from the transform approach used in He et al. [23] and related discrete-monitoring work [9,10,17]. Verification and conditioning on the chain states its damped-limit justification. Only the real part has the removable lower-endpoint limit


$$\begin{array}{c} \lim_{\psi ↓0}ℜ\frac{K_{\chi }(\psi -j)-K_{\chi }(\psi )}{j\psi }=𝔼[(e^{Y}-1)Y]. \end{array}$$

The full complex quotient can have an imaginary 1/ψ term when $𝔼[e^{Y}]\neq 1$. Our Gauss–Legendre rule integrates from zero without evaluating the endpoint. The upper bound is chosen adaptively from the per-interval return scale and checked jointly with quadrature refinement; no fixed range such as 50–100 is asserted to suffice at every frequency.

## Numerical analysis

### Parameter settings and implementation

The baseline is an illustrative, uncalibrated parameterization inherited from the numerical example of He et al. [23], with the added feedback coefficient. It is not an estimate of a particular market. Table 1 specifies *T*, *X*_0_ and the liquidity-scale convention. Time is in years; ϵ=1 corresponds to a variance-factor reversion half-life log2≈0.693 years, so it is not described as fast mean reversion. The illustrative $\rho_{2}=-0.30$ is not an empirically established price-liquidity leverage estimate. The European-option estimates in reference [23] concern a different fit (including a positive $\rho_{2}$) and do not validate this sign choice. Our correlation sweep includes negative, zero and positive values.

**Table 1:** Illustrative parameters; row-generator convention. The initial liquidity value fixes the scale and is held constant in every sweep.

Parameter	Value	Parameter	Value
ϵ	1.00	$\rho_{2}$	−0.30
$\tilde{\delta }$	(0.08, 0.12)	σ	0.20
ω	0.14	χ	1.00
$\tilde{\gamma }$	2.00	*g*_12_	0.10
$\tilde{\alpha }$	0.30	*g*_21_	0.40
β	0.40	$\eta_{0}$	0.15
*r*	0.04	$\lambda_{0}$	0.20
$\rho_{1}$	−0.50	*T* (years)	1.00
*X*_0_	*e*_1_	*m*	100
*N* (baseline)	12	$\lambda_{0}$ scale	fixed at 0.20

The revised Python implementation integrates the complex Riccati and chain equations with DOP853 at rtol = 10^–10^ and atol = 10^–12^. No exponent clipping is used. Both integrations end exactly at the stage junction; the second starts from the first’s terminal state. Complex arithmetic is used directly, with an independent real/imaginary-stacked implementation for cross-checking. On an equally spaced grid, the common active-return stage is solved once per Fourier argument, and the earlier-stage semigroup is evaluated at all n−1 interval lengths. This reuses identical coefficients without changing the two-stage representation. The finite-state chain is integrated jointly with the Riccati equations; a time-dependent source is not replaced by one ordinary matrix exponential.

Fourier integration uses Gauss–Legendre nodes on (0,*U*), so no lower interval is discarded. Start with $U_{0}=\max\{40,8\sqrt{N/[T(\eta_{0}+\sigma^{2}\lambda_{0}^{2})]}\}$ and 64 nodes. Compare (*U*,*n*) with (*U*,2*n*) and (2*U*,4*n*), enlarging/refining until both price differences are below $2\times 10^{-6}$. This is an empirical convergence criterion, not a rigorous tail-error bound. The accepted extended grid is used for production. Appendix B. Numerical settings, convergence and reproducibility separates domain, node and ODE checks. The complete per-case parameters and final grids are saved in results/prices.csv in the supplementary code package.

Monte Carlo uses full truncation for the variance factor, an Euler OU step, a log-price step and the exact grid-point chain transition $\exp(G^{\mathbb{Q}}\Delta t)$. The grid contains every monitoring date: a requested step count *L* gives N⌈L/N⌉ actual steps over *T* = 1. Grid-point chain transitions are exact, but within-step occupation and diffusion integration remain discretized. Each run has 300,000 paths comprising 150,000 independent antithetic pairs. The paired Brownian increments have opposite signs, and paired chain uniforms are *u* and 1−u. Standard errors are computed from the pair-averaged payoffs, not from 300,000 supposedly independent observations. The seed is 20260626 + *N* for validation and 20260626 for the step diagnostic. Reported intervals are pointwise $\bar{P}\pm 1.96\hat{se}$ and quantify sampling error, not all discretization bias.

### Formula validation

Tables 2–3 and Figs 1–2 compare the recomputed transform prices with simulations. The retained *N* = 1 case is a coarse-sampling stress test, not a representative traded-contract design. Absolute prices in the two legs have different units and are not directly comparable measures of sensitivity. Changes across monitoring frequencies reflect changes in the defined simple-return payoffs, rather than changes in the accuracy of an otherwise exact transform formula. The corrected pointwise intervals contain the transform prices at the reported settings. The step-refinement experiment is reported in Appendix B. Numerical settings, convergence and reproducibility; interval overlap alone is not a proof of zero time-discretization bias.

**Table 2:** Monte Carlo validation: *T* = 1, 300,000 paths (150,000 independent antithetic pairs); confidence intervals use pair means. Variance leg.

*N*	Steps	$P_{A}$	MC mean	95% interval
1	1000	1804.9530	1817.71	[1801.94, 1833.48]
4	1000	1650.0657	1647.68	[1641.46, 1653.90]
12	1008	1619.9988	1620.82	[1617.38, 1624.26]
52	1040	1608.8319	1609.96	[1608.27, 1611.64]
104	1040	1607.1752	1607.18	[1605.95, 1608.41]
252	1008	1606.2044	1606.42	[1605.55, 1607.29]

**Table 3:** Monte Carlo validation: Absolute-return leg, same settings and paired confidence intervals.

*N*	Steps	$P_{O}$	MC mean	95% interval
1	1000	40.4646	40.5638	[40.4047, 40.7229]
4	1000	39.8929	39.8739	[39.7977, 39.9501]
12	1008	39.7751	39.7817	[39.7377, 39.8257]
52	1040	39.7311	39.7372	[39.7156, 39.7588]
104	1040	39.7245	39.7263	[39.7108, 39.7419]
252	1008	39.7207	39.7210	[39.7103, 39.7317]

**Fig 1:**
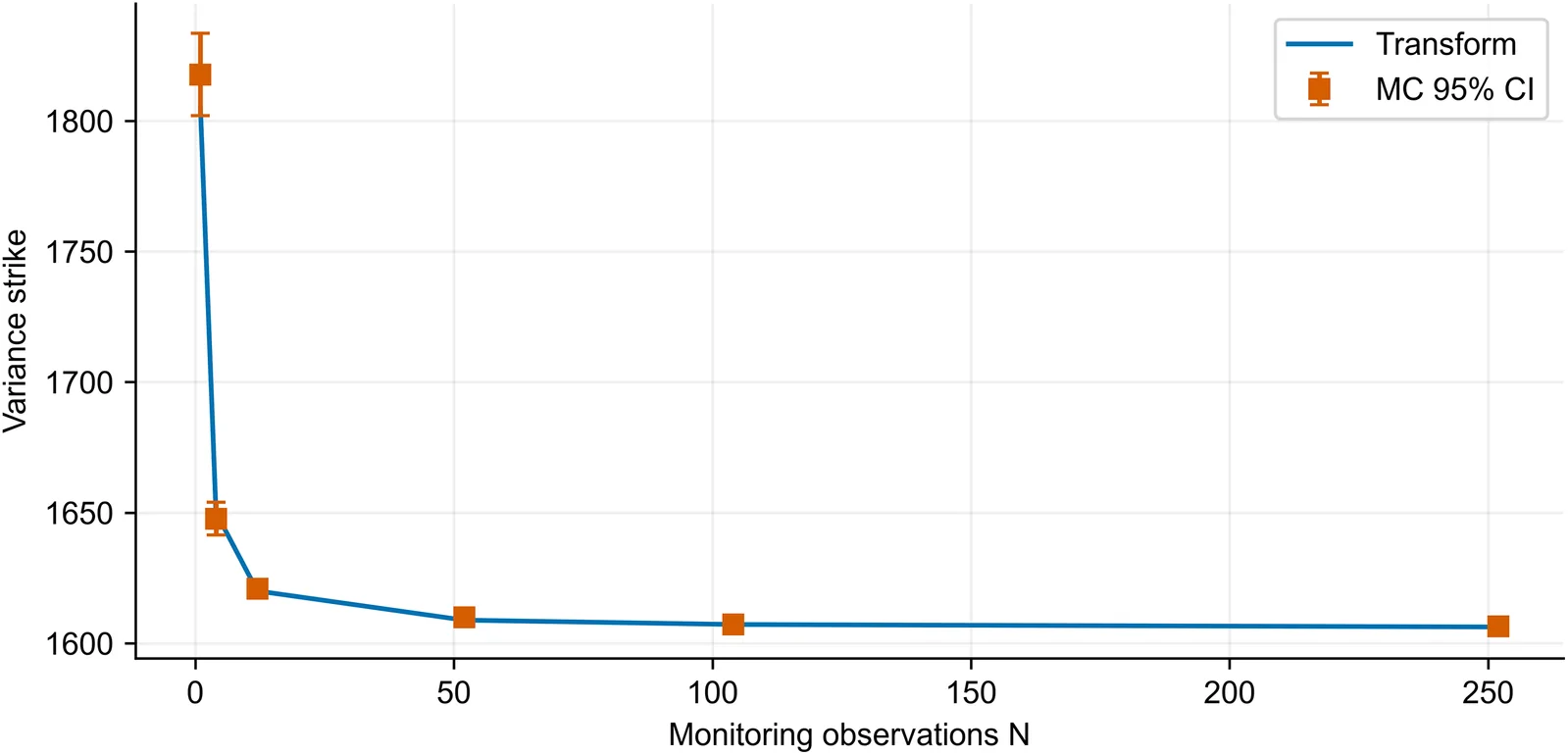
Variance-swap validation (VAL); error bars are paired 95% Monte Carlo intervals.

**Fig 2:**
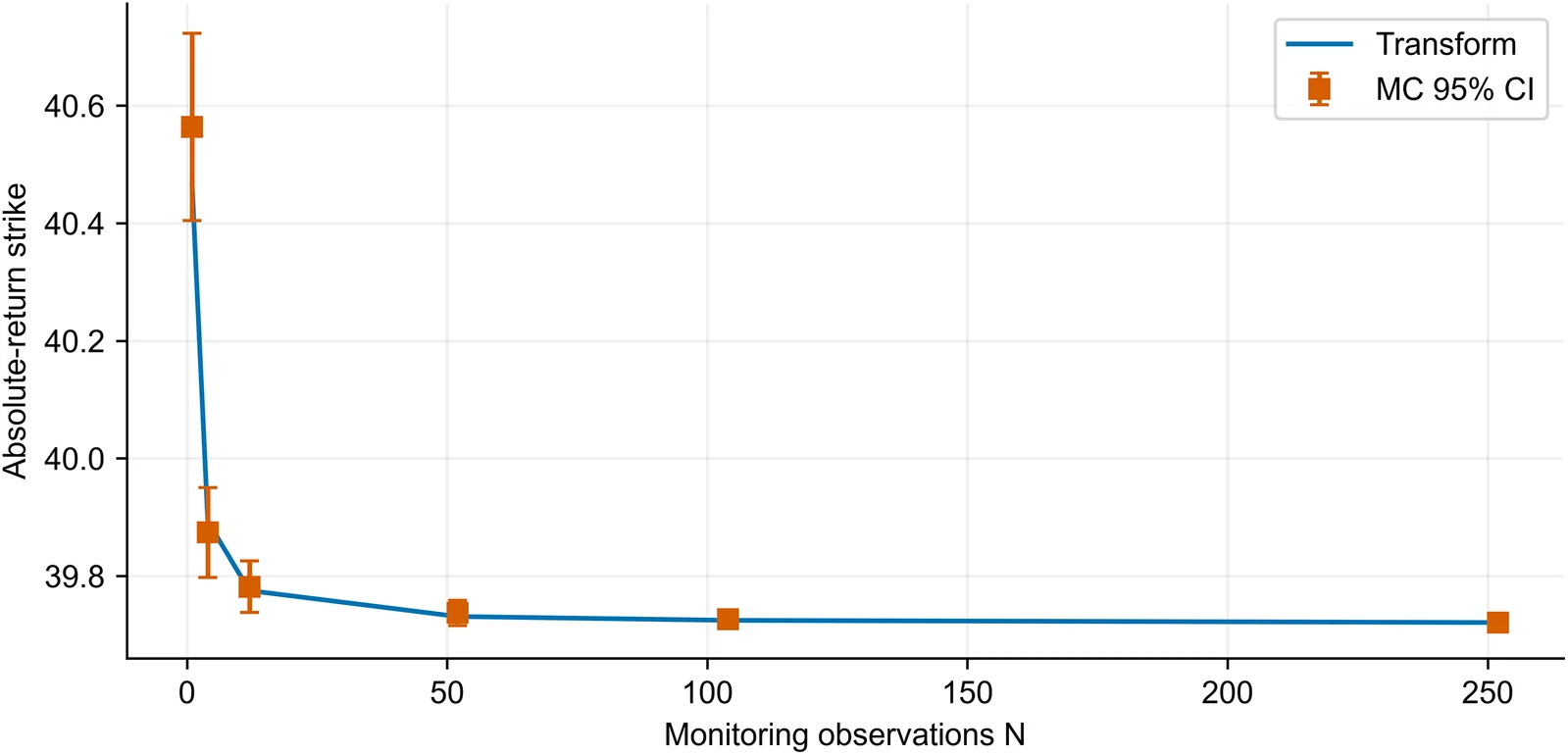
Absolute-return-swap validation (VAL); same simulation settings.

### Nonlinear feedback effects

Fig 3 shows how the quadratic target shift raises both contract strikes in the illustrative scenarios. The percentage contribution $(P^{\chi }-P^{0})/P^{0}$ in Fig 4 compares relative changes across their different quotation units. It is a model-implied price contribution, not a risk premium: no physical-measure model or market price of risk is estimated here. The larger relative variance-leg response is an observed property of these experiments. It is not attributed to concavity of an absolute-value payoff, which is not concave.

**Fig 3:**
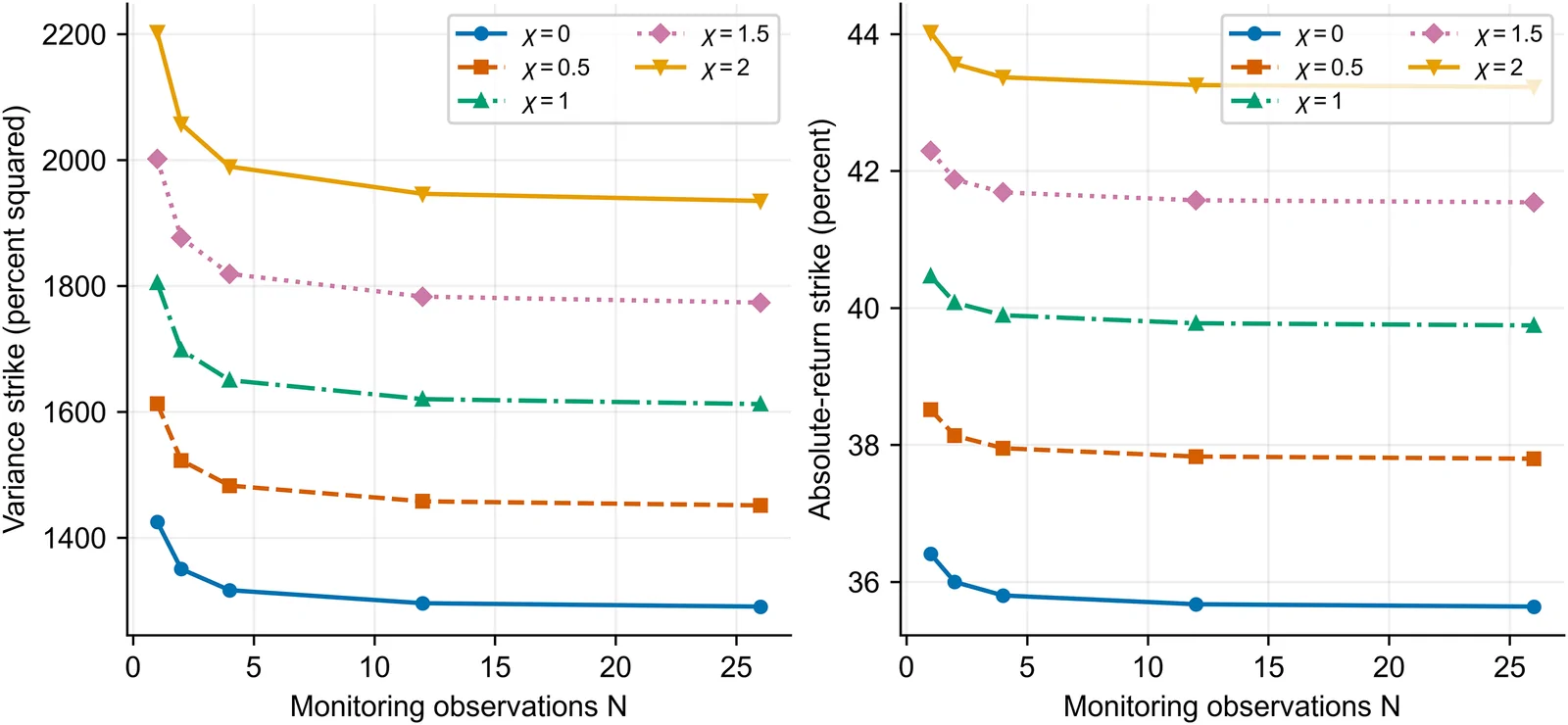
Feedback effects across monitoring frequencies (CHI); *N =* 1 is a stress case.

**Fig 4:**
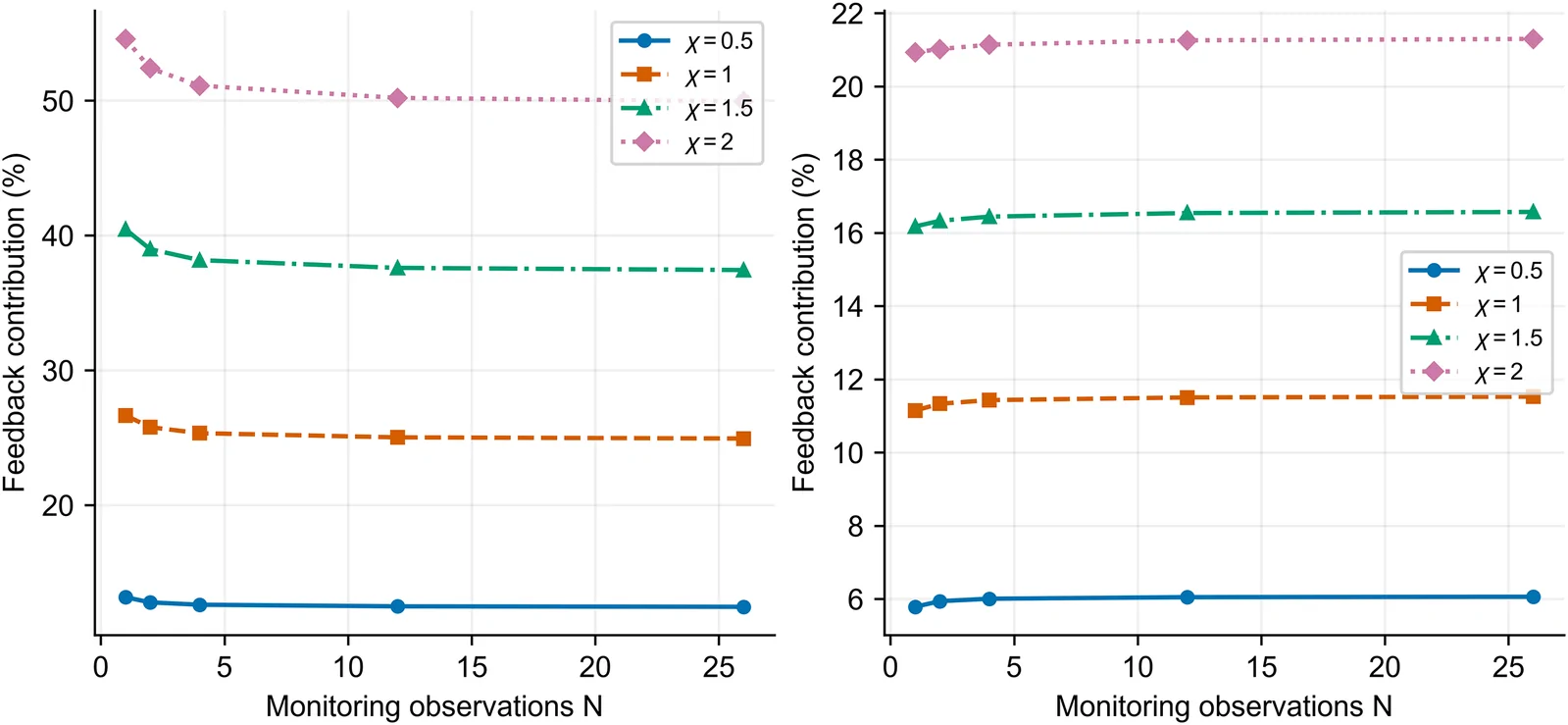
Relative feedback contributions (CHI).

### Liquidity-stress level, persistence and volatility

The OU second moment satisfies the exact identity


$$\begin{array}{c} 𝔼[\lambda_{t}^{2}]={\left[\tilde{\alpha }+(\lambda_{0}-\tilde{\alpha })e^{-\tilde{\gamma }t}\right]}^{2}+\frac{\beta^{2}}{2\tilde{\gamma }}(1-e^{-2\tilde{\gamma }t}). \end{array}$$ (38)

Fig 5 varies the positive long-run mean over the stated range. With the fixed positive initial factor, this increases the squared-mean contribution; this is not a global monotonicity claim for arbitrary signed means. Fig 6 varies persistence. Since $\lambda_{0}<\tilde{\alpha }$, faster reversion increases the early conditional mean but reduces dispersion, so competing effects can produce a weak or non-monotone price response. Fig 7 increases liquidity dispersion, which affects the target through $\chi 𝔼[\lambda_{t}^{2}]$. In all three sweeps the scale convention $\lambda_{0}=0.20$ is held fixed.

**Fig 5:**
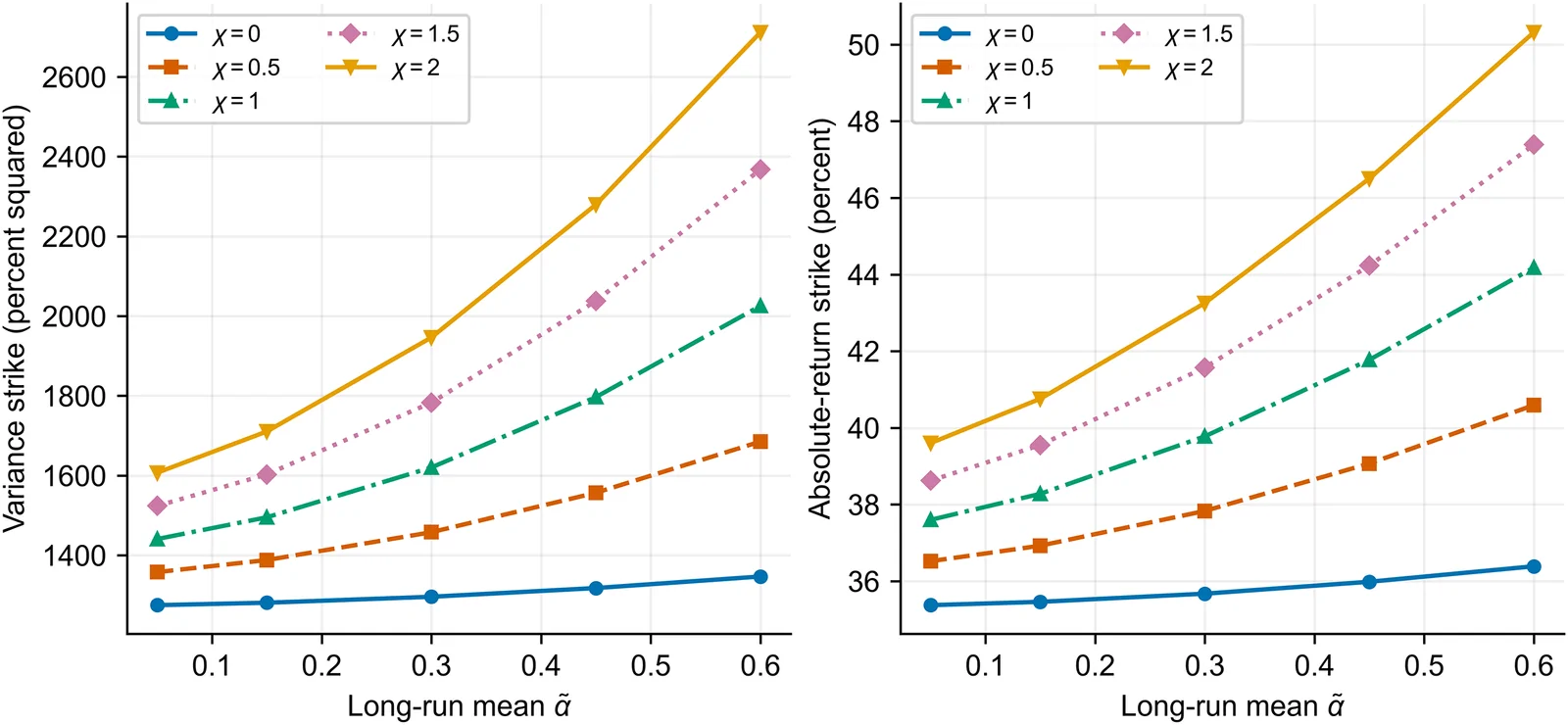
Long-run liquidity mean and feedback (ALPHA).

**Fig 6:**
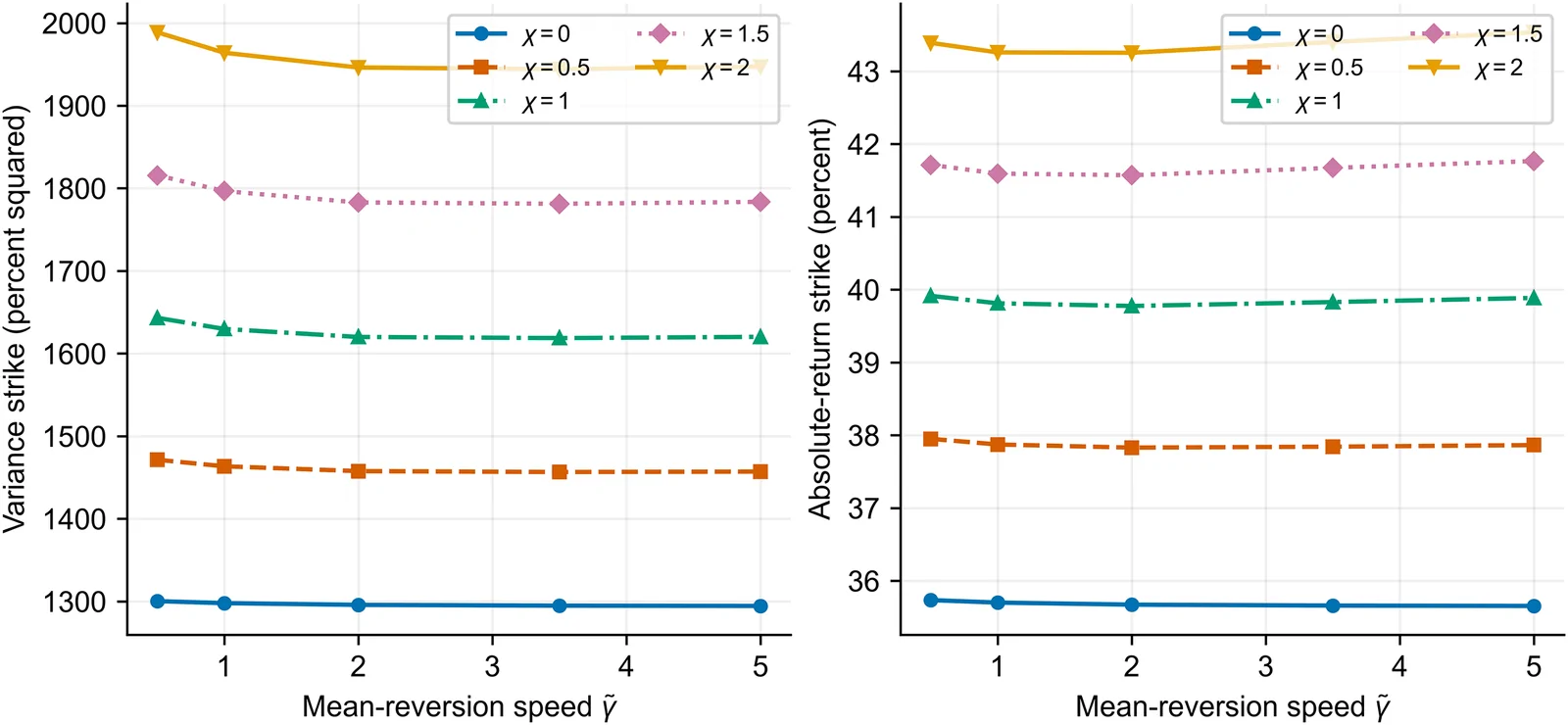
Liquidity persistence and feedback (GAMMA).

**Fig 7:**
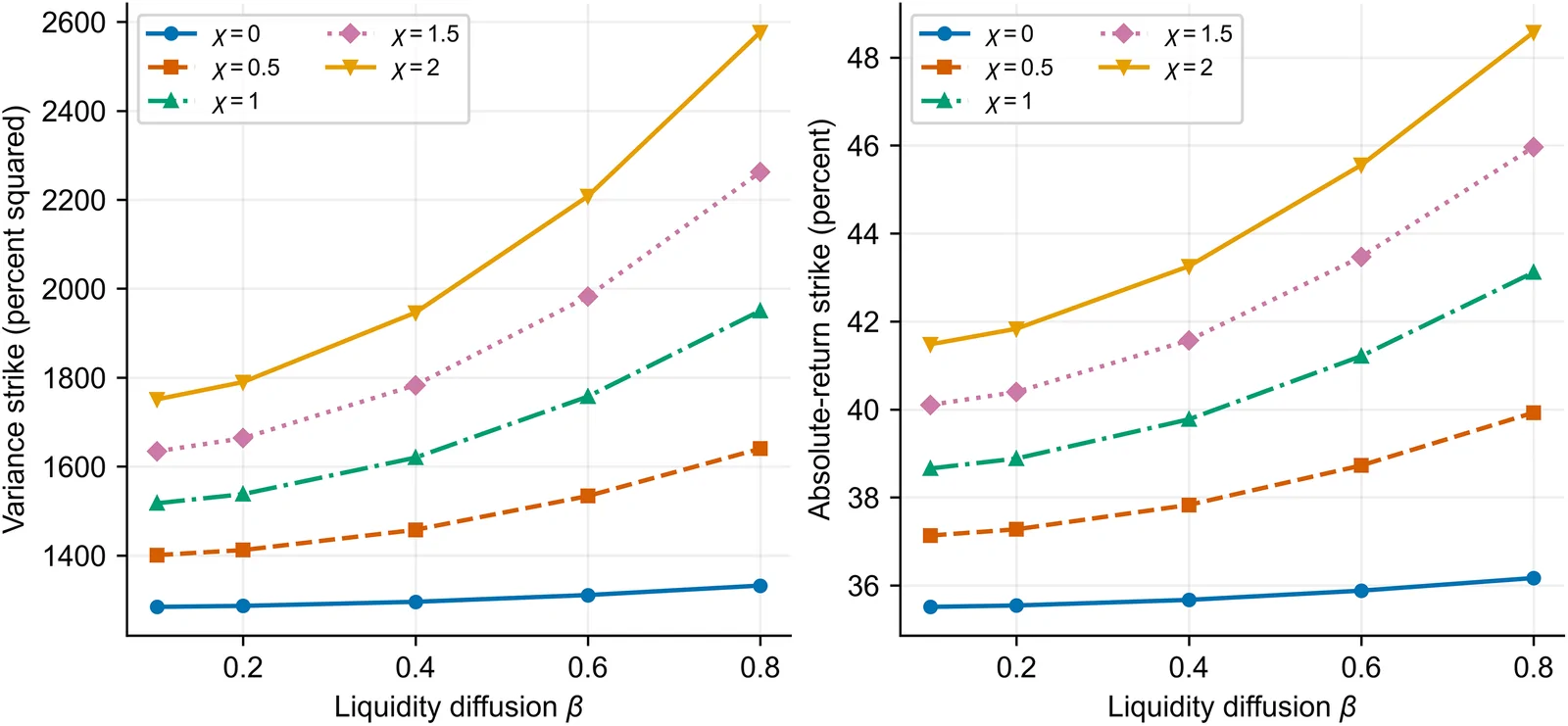
Liquidity diffusion and feedback (BETA).

### Correlation effects

The correlation $\rho_{1}$ enters the return–variance mixed derivative and the linear coefficient of the variance Riccati equation; $\rho_{2}$ enters the return–liquidity mixed derivative. Fig 8 separates these sweeps, retaining all other parameters at baseline. Over $\rho_{1}\in [-0.75,0.75]$, the variance strike changes from 1615.5112 to 1642.6992 (+1.683%), and the absolute-return strike from 39.7695 to 39.8083 (+0.098%). Over $\rho_{2}\in [-0.75,0.75]$, the variance strike changes from 1619.6313 to 1620.8661 (+0.076%), and the absolute-return strike from 39.7745 to 39.7746 (+0.000%). These are conditional model sensitivities, not empirical estimates or tests of statistical significance. The finite-horizon certificates hold at every positive and negative correlation setting used here.

**Fig 8:**
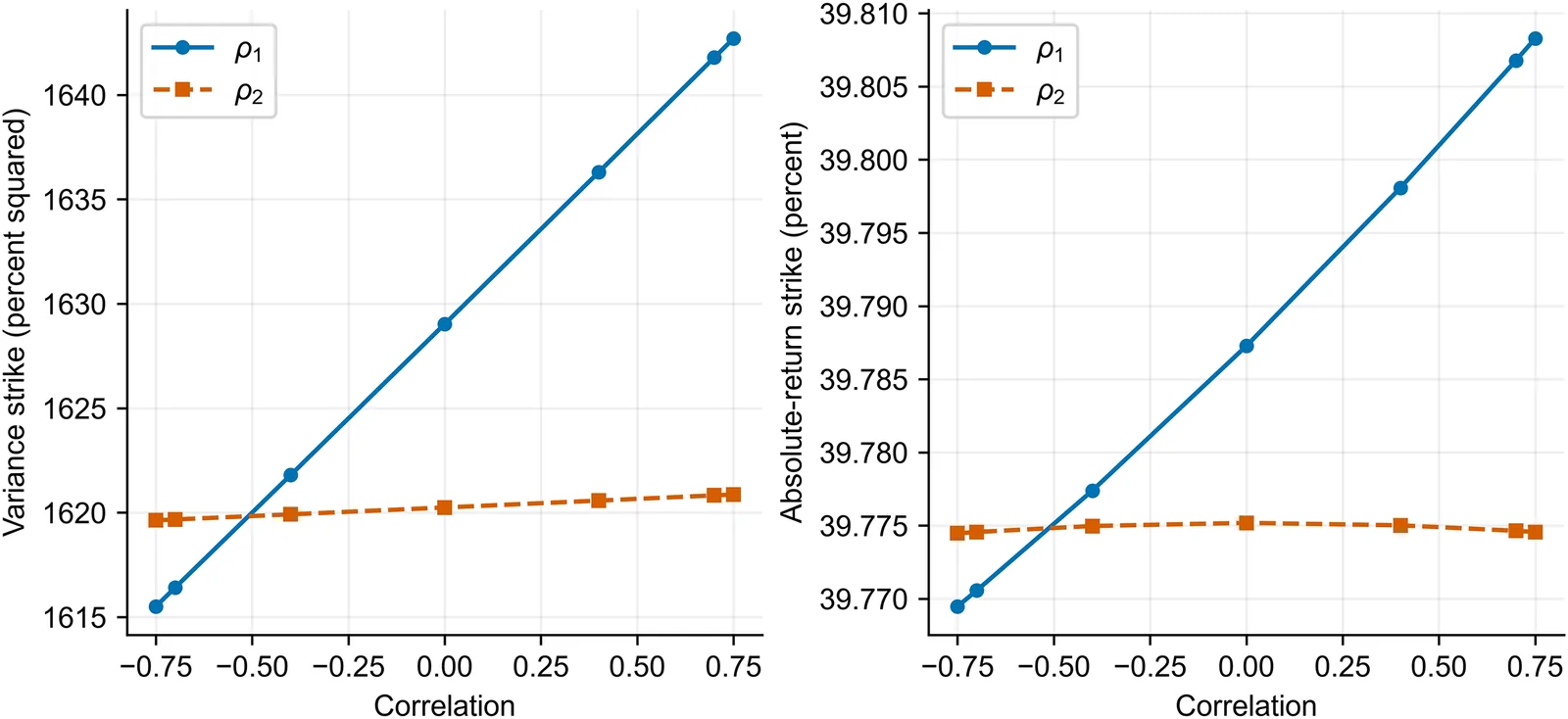
Separate correlation sweeps at *N =* 12, *T =* 1 (RHO).

### Nested benchmarks and numerical convergence

Fig 9 and Table 4 remove each liquidity channel in turn. The no-feedback model retains direct return liquidity; only setting both χ and σ to zero removes both channels. All values were regenerated with the revised production settings.

**Table 4:** Nested benchmark at *N* = 12, *T* = 1; settings NEST.

Specification	$P_{A}$	$P_{O}$
full	1619.9988	39.7751
no feedback	1295.7203	35.6716
no direct	1580.7111	39.3105
neither	1256.5192	35.1149

**Fig 9:**
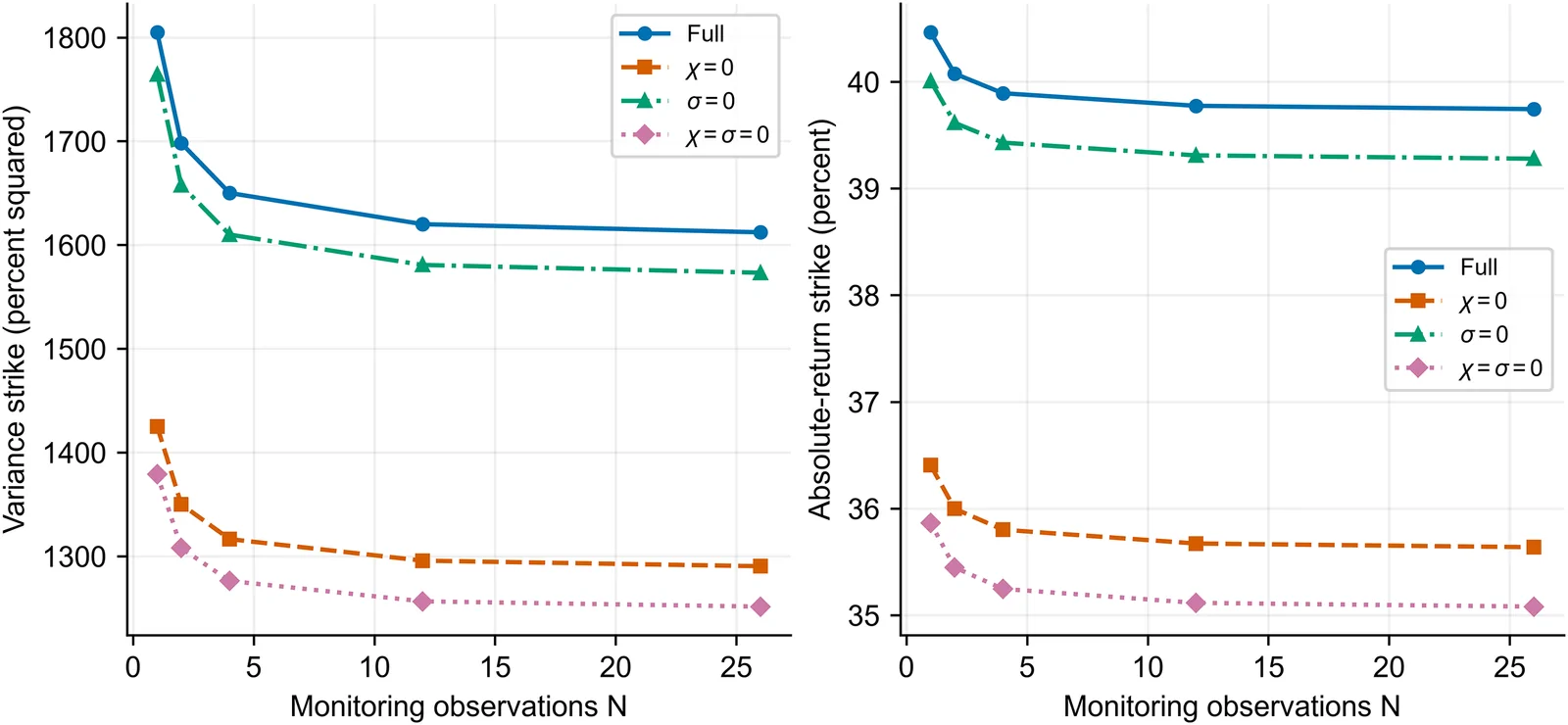
Nested liquidity specifications (NEST).

At *N* = 12, removing feedback reduces the variance strike by 324.2784 and the absolute-return strike by 4.1035; removing the direct channel reduces them by 39.2877 and 0.4646, respectively. Define the cross-channel interaction as


$$\begin{array}{c} I=P(\chi ,\sigma )-P(0,\sigma )-P(\chi ,0)+P(0,0). \end{array}$$

The interactions are + 0.0866 and −0.0921, respectively, or 0.024% and −1.976% of their total two-channel increments. Thus the channels are approximately additive at baseline; the quadratic state dependence chiefly acts within the feedback channel. This decomposition is parameter-dependent. Table 5 reports dimensionless contributions at other feedback strengths.

**Table 5:** Feedback contribution relative to χ=0, in percent; *N* = 12, *T* = 1, settings CHI.

χ	Variance (%)	Absolute-return (%)
0.50	12.4952	6.0492
1.00	25.0269	11.5035
1.50	37.5951	16.5402
2.00	50.2001	21.2570

The original tolerance table is reproducible with the historical unsplit Markov integration. At *N* = 12, its 10^–7^ and 10^–8^ prices are 1619.943884 and 1620.053829, whereas explicitly ending the chain integration at the junction gives 1619.998789 and 1619.998779 with the same tolerances. An adaptive solver can miss a short piece of the source, and local error tolerances do not enforce monotonicity of the final cancellation-sensitive price error. The revised joint, split integration gives 1619.998771 and is checked against the independently split historical implementation and a second solver. The corrected *N* = 252 variance strike is 1606.204436, rather than 1606.510890. These changes propagate to all tables and figures. Appendix B. Numerical settings, convergence and reproducibility reports convergence and actual settings instead of recommending a tolerance that was not applied.

#### External no-feedback comparison.

He et al. [23], p. 11, uses the same no-feedback example parameters and *T* = 1. Its Fig 1(a) provides a graph rather than tabulated strike data. Table 6 extracts the vector path using the plotted axis limits (*N*:0–150, strike:1280–1440); graph values are quoted to 0.1 with an approximate 0.5-unit reading tolerance. Differences use unrounded converted ordinates: at *N* = 1 the extracted value is 1426.977841 and the difference is −1.8622, while at *N* = 6 the ordinate is 1305.948971 and the difference is approximately zero at the displayed precision. These extra digits document the coordinate conversion, not the accuracy of the published graph. The comparison uses squared simple returns, *m* = 100 percentage quotation, the displayed example parameters, and the plotted monitoring frequencies; the 0.5-unit reading tolerance is not a statistical confidence interval or a solver error bound. We condition on $X_{0}=e_{1}$, as in the present implementation; that page of reference [23] does not explicitly state its initial regime. The maximum selected discrepancy is about 1.86 (0.131%) at *N* = 1, beyond graphical rounding, while several higher-frequency values differ by approximately 0.4. Consequently this is a quantified graphical reconciliation, not a claim of exact reproduction of unpublished numerical data. The remaining difference cannot be assigned to a particular cause without the predecessor’s code and complete settings.

**Table 6:** No-feedback comparison with vector-extracted Fig 1(a) of He et al.; *T* = 1, $X_{0}=e_{1}$. Published graph ordinates are displayed to 0.1. Difference uses the unrounded coordinate-converted ordinate, retained with the PDF coordinates in the reproduction package. Its two decimals do not imply accuracy beyond the approximate 0.5-unit graph-reading tolerance.

*N*	Published graph	Recomputed	Difference
1	1427.0	1425.1156	−1.86
4	1316.7	1316.5013	−0.22
6	1305.9	1305.9490	+0.00
40	1288.4	1288.7568	+0.40
75	1287.0	1287.3838	+0.43
150	1286.2	1286.6022	+0.45

### Two-dimensional nonlinear diagnostics

Fig 10 displays the joint χ–β sensitivity on the same grid used by the one-dimensional experiments. Larger dispersion and feedback jointly raise prices in this grid. Because the two legs have different units, gradients across panels are not compared as absolute measures of economic sensitivity. Fig 11 gives $𝔼[{\tilde{\delta }}_{X_{t}}+\chi \lambda_{t}^{2}]$, calculated using $e^{G^{\mathbb{Q}}t}$ and (38), and illustrates the target mechanism without treating it as independent market evidence.

**Fig 10:**
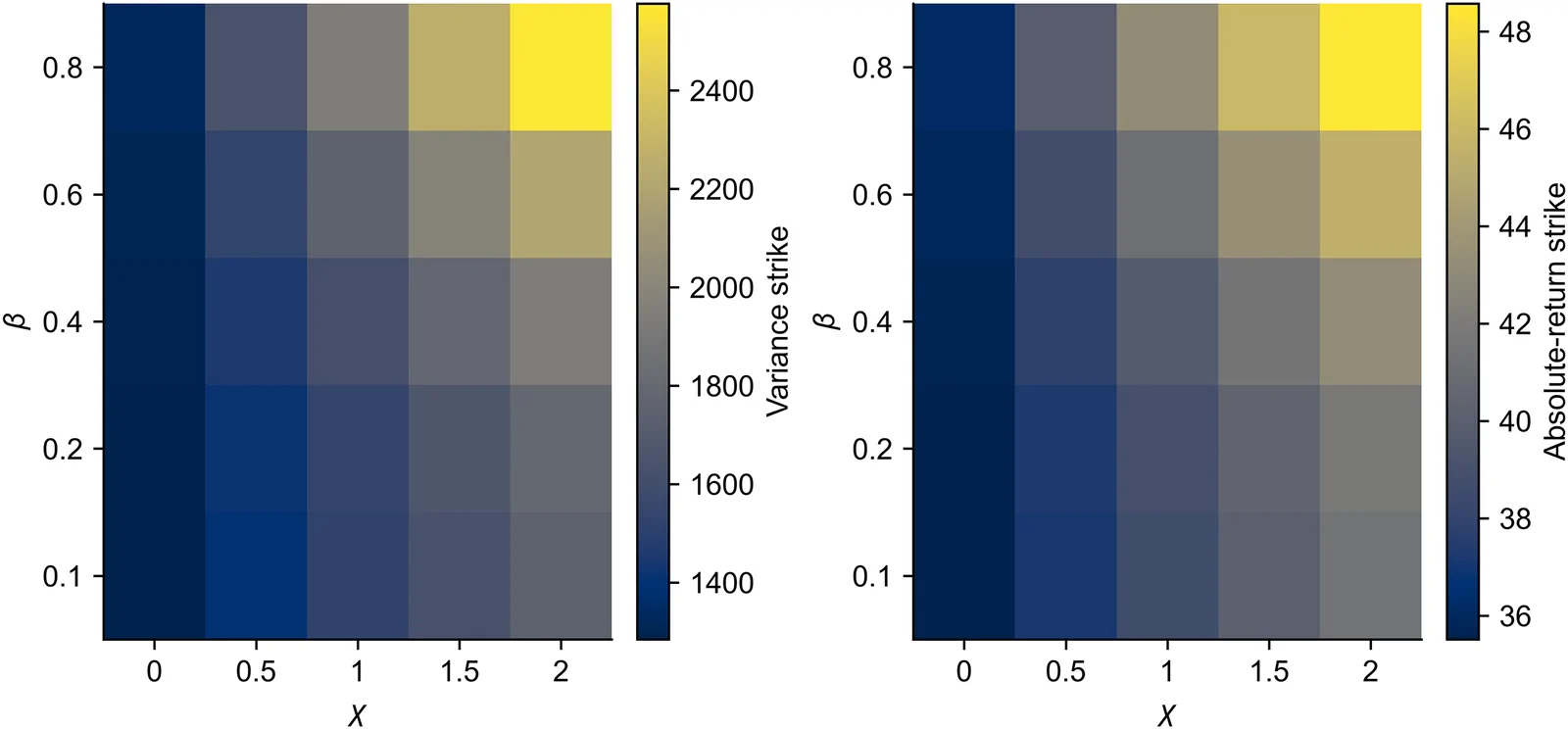
Joint feedback and liquidity-diffusion scenarios (BETA); discrete parameter levels are labeled explicitly.

**Fig 11:**
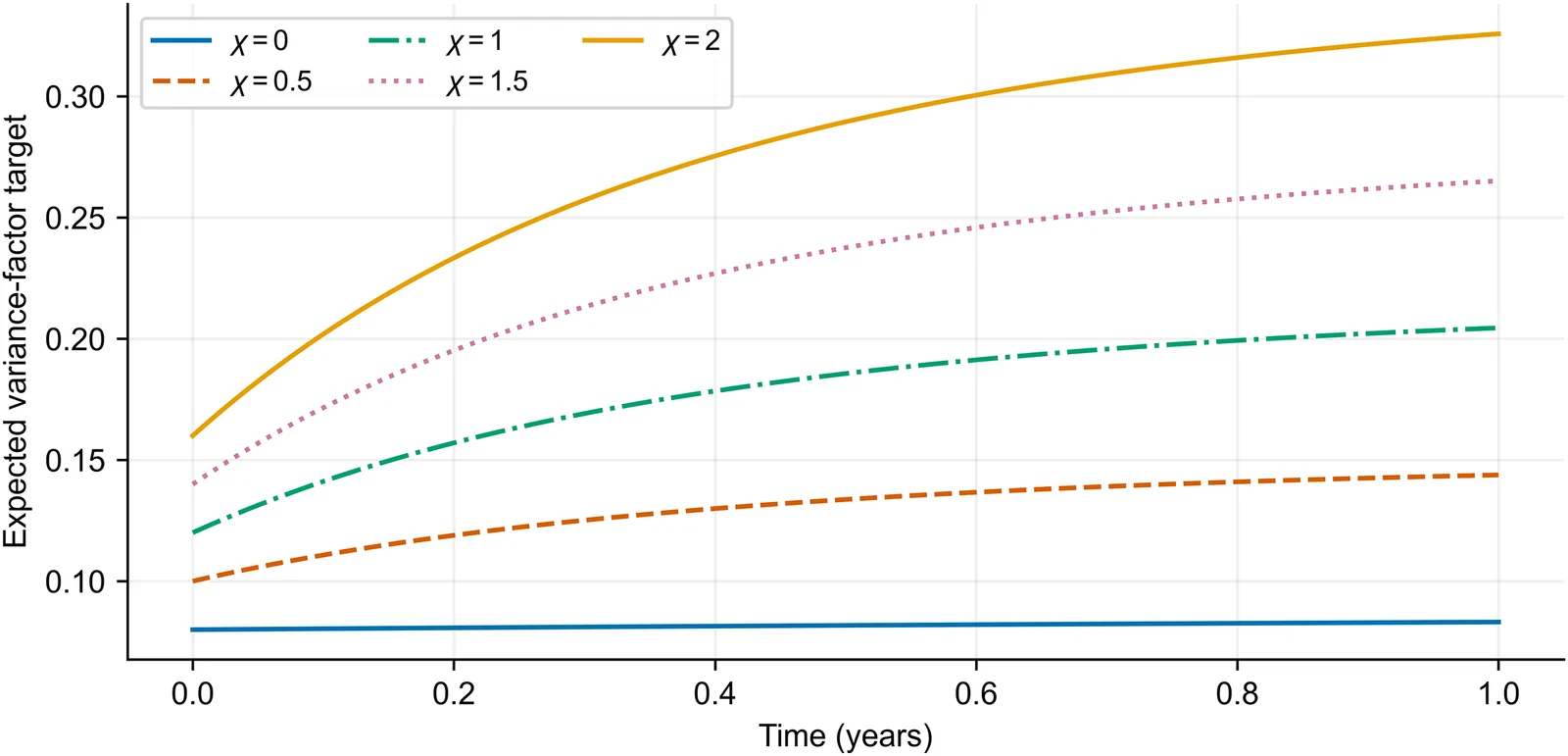
Expected effective variance-factor target (TARGET).

### Economic summary of the numerical mechanisms

In these uncalibrated experiments, delayed feedback dominates direct return liquidity and relative variance-leg contributions exceed those of the absolute-return leg. Near-additivity at baseline does not remove the quadratic dependence on the signed liquidity state. Neither channel ranking nor parameter magnitudes should be generalized to market data without estimation and identification checks.

#### Calibration and nonlinear-payoff limitations.

Fixing the liquidity scale removes one redundant parameter, but it does not establish empirical identification of all remaining coefficients. A practical study would need a measured liquidity proxy or informative restrictions, joint option/return/liquidity information, admissibility-constrained estimation, multi-start optimization and out-of-sample comparison with simpler nested models. Fixing the ordering of the regime targets would also prevent regime-label ambiguity. We do not claim that this calibration has been performed or is necessarily stable.

The sum of single-period expectations suffices for the two additive contract measures studied here. It does not directly price $𝔼[\sqrt{RV_{A}}]$ or an option such as $𝔼[(\sqrt{RV_{A}}-K{)}^{+}]$, which requires the temporal dependence of the whole realized-variance functional. Path simulation or an enlarged state/appropriate joint transform is needed. Section 3.2 of Horvath et al. [11] discusses simulation for volatility options; Proposition 5 of Wang and Xia [13] uses a transform of average forward variance for power options in a different model. Those results motivate possible extensions but are not interchangeable formulas for this paper’s discretely realized variance. A rough-volatility extension would introduce memory beyond the present finite-dimensional closure.

## Conclusion

This paper adds a quadratic liquidity-stress feedback term to a stochastic-liquidity and regime-switching variance model. Within the established exponential-quadratic transform class, the additional Riccati forcings yield a forward transform for discretely sampled variance and absolute-return-based volatility contracts. Explicit sufficient conditions, a verification argument and independently checked numerical implementation clarify the domain in which these formulas apply. The absolute-return contract is distinct from a conventional square-root-of-realized-variance volatility swap.

The recomputed, uncalibrated baseline experiments show a larger contribution from delayed feedback than from direct liquidity exposure, with small cross-channel interactions. Liquidity mean, dispersion and persistence affect prices through the second moment of the signed OU factor; correlation effects are quantified rather than asserted to be statistically significant. These findings remain conditional on the specified parameters, scale convention and zero variance–liquidity Brownian correlation. Robust market calibration and nonlinear options on the entire realized-variance path are outside the demonstrated results.

## Appendix

### Appendix A. Explicit finite-horizon bounds and transform verification

The following sufficient tests depend only on parameters and the chosen horizon. They are deliberately conservative and are not necessary conditions. Failure of a test is not evidence of moment explosion. The connection between exponential moments and Riccati equations is classical [29,30]; the bounds below include the additional liquidity-feedback forcing explicitly.

#### An elementary scalar comparison function.

Let *R*(*A*,*a*,*q*;*t*) solve $R^{\prime }=AR^{2}-aR+q$, *R*(0) = 0, where *A* > 0 and q≥0. Set T(A,a,q) equal to its first explosion time. For *q* = 0, *R* = 0 and T=∞. For *q* > 0 and $d^{2}=a^{2}-4Aq>0$,


$$\begin{array}{c} R(A,a,q;t)=\frac{2q(1-e^{-dt})}{(a+d)-(a-d)e^{-dt}},\;d=\sqrt{a^{2}-4Aq}. \end{array}$$ (A.1)

Here T=∞ if *a* > 0, and $T=d^{-1}\log((a-d)/(a+d))$ if *a* < 0. If *a*^2^ = 4*Aq*, then *R* = *qt*/(1 + *at*/2), with T=∞ for *a* > 0 and −2/a for *a* < 0. If $d^{2}=4Aq-a^{2}>0$,


$$\begin{array}{c} R(A,a,q;t)=\frac{a+d\tan(dt/2-\arctan(a/d))}{2A},\;T(A,a,q)=\frac{\pi +2\arctan(a/d)}{d}. \end{array}$$ (A.2)

These expressions are used only for t<T. Thus no numerical ODE non-explosion assumption is needed to evaluate the tests. In particular, the positive-root argument for the second-return-moment variance block requires both $\epsilon -2\rho_{1}\omega >0$ and $(\epsilon -2\rho_{1}\omega {)}^{2}>2\omega^{2}$; the discriminant alone does not ensure positive roots.

#### Novikov bound.

Choose $\nu =\tilde{\gamma }/2>0$ and define


$$\begin{array}{c} B_{N}=R(\omega^{2}/2,\epsilon ,1/2;T),\;q_{N}=\epsilon \chi B_{N}+\sigma^{2}/2. \end{array}$$

The explicit sufficient condition is


$$\begin{array}{c} T<T(\omega^{2}/2,\epsilon ,1/2),\;T<T(2\beta^{2},2\tilde{\gamma }-\nu ,q_{N}). \end{array}$$ (A.3)

To see this, use the exponential supersolution $V(s,\eta ,\lambda )=\exp\{A(s)+B(seta+D(s)\lambda^{2}\}$, with zero initial coefficients. Take $B^{\prime }=\omega^{2}B^{2}/2-\epsilon B+1/2$, and let


$$\begin{array}{c} D^{\prime }=2\beta^{2}D^{2}-(2\tilde{\gamma }-\nu )D+q_{N},\;A^{\prime }=\epsilon \delta_{\max}B+\beta^{2}D+{\tilde{\gamma }}^{2}{\tilde{\alpha }}^{2}D/\nu , \end{array}$$

where $\delta_{\max}=\max_{i}{\tilde{\delta }}_{i}$. The inequalities $B(s)\leq B_{N}$ and $2\tilde{\gamma }\tilde{\alpha }D\lambda \leq \nu D\lambda^{2}+{\tilde{\gamma }}^{2}{\tilde{\alpha }}^{2}D/\nu$ give $\partial_{s}V\geq L_{\eta ,\lambda ,X}V+(\eta +\sigma^{2}\lambda^{2})V/2$. The switching term is zero because this upper bound is regime independent. Itô’s formula stopped on compact sets, followed by Fatou’s lemma and V≥1, gives


$$\begin{array}{c} 𝔼\exp\left\{\frac{1}{2}\int_{0}^{T}(\eta_{s}+\sigma^{2}\lambda_{s}^{2},ds\right\}\leq V(T,\eta_{0},\lambda_{0})<\infty . \end{array}$$

This proves Novikov’s condition on the specified horizon. For an isolated OU quadratic integral the familiar all-horizon threshold is $q<{\tilde{\gamma }}^{2}/(2\beta^{2})$; for an isolated CIR variance integral it is $q<\epsilon^{2}/(2\omega^{2})$. The coupled process is not an independent CIR process: $q_{N}$ contains the additional $\epsilon \chi B_{N}$ term. Our finite-horizon criterion also includes slack for the nonzero OU mean.

#### A strip bound covering both stages.

Fix *h* > 0, put *p*_+_ = 2 + *h*, $q_{+}=p_{+}(p_{+}-1)/2$, and set


$$\begin{array}{c} a=\epsilon -\omega \max\{p_{+}\rho_{1},-h\rho_{1}\},\;b=\tilde{\gamma }-\beta \sigma \max\{p_{+}\rho_{2},-h\rho_{2}\},\;B_{*}=R(\omega^{2}/2,a,q_{+};T). \end{array}$$

A sufficient test for the entire return-moment strip −h≤p≤2+h is


$$\begin{array}{cc} T & <T(\omega^{2}/2,a,q_{+}),\\ T & <T(2\beta^{2},2b-\nu ,\epsilon \chi B_{*}+q_{+}\sigma^{2}). \end{array}$$ (A.4)

For clarity, *p* denotes a real exponential loading: ψ=u−jp. To prove the bound, repeat the preceding supersolution argument with $B^{\prime }=\omega^{2}B^{2}/2-aB+q_{+}$ and $D^{\prime }=2\beta^{2}D^{2}-(2b-\nu )D+\epsilon \chi B_{*}+q_{+}\sigma^{2}$. The log-return generator contributes $p\rho_{1}\omega B$ to the η coefficient, $2p\rho_{2}\beta \sigma D$ to the $\lambda^{2}$ coefficient and $p(p-1)(\eta +\sigma^{2}\lambda^{2})/2$. These are bounded by the displayed coefficients for every *p* in the strip. Add *p*_+_|*r*| to $A^{\prime }$ to bound the constant term. The same upper bound applies when the return loading is zero before the monitored interval. Applied to the stochastic integral for the return accumulated only on $\left[u_{2},u_{3}\right]$, the stopped supersolution therefore bounds its exponential moment over the entire horizon $u_{3}-u_{1}\leq T$, including the pre-monitoring stage. There is no assumption of independence between η and λ.

Consequently $𝔼[e^{pY}]<\infty$ for the monitored increment *Y*, throughout this strip, and $\left|𝔼[e^{(p+ju)Y}]|\leq 𝔼[e^{pY}\right]$ for every real *u*, not merely on the implemented Fourier grid. The strict inequalities allow a small further enlargement of the exponents and horizon by continuity. The stopped change-of-measure argument below verifies the state-weighted transform candidate. In particular, $𝔼[(1+e^{Y})|Y|]<\infty$; the lower endpoint of the real Fourier integrand is well defined. In the numerical study *h* = 0.1 and every setting is checked separately. The conditions are sufficient at *T* = 1; they are not a claim of global moment existence for arbitrary parameters or maturities.

#### Verification and conditioning on the chain.

Condition on a complete path of *X* over $\left[u_{1},u_{3}\right]$. Independence of the Brownian vector and *X* leaves Brownian innovations with their original law under this conditioning. The path of ${\tilde{\delta }}_{X}$ is now a bounded, piecewise constant deterministic function. Substituting the exponential-quadratic candidate in the diffusion backward equation gives exactly the two Riccati systems in Proposition 2. The terminal data of the earlier stage are the later-stage coefficients, so the first stage is not restarted at zero. The scalar path-dependent factor is


$$\begin{array}{c} \exp\left\{\int_{u_{1}}^{u_{3}}\epsilon \;\hat{B}(t){\tilde{\delta }}_{X_{t}}\;dt\right\},\;\hat{B}(t)=\left\{ \begin{array}{cc} \xi_{6}(u_{2}-t), & t\leq u_{2},\\ \xi_{2}(u_{3}-t), & t>u_{2}. \end{array}\right. \end{array}$$

This factorization does not assert independence of *X* and the variance process. It follows because the Riccati coefficients are independent of the regime, and only the additive scalar coefficient contains ${\tilde{\delta }}_{X}$.

For a real loading p∈[−h,2+h], write the path-conditioned backward candidate as $F_{t}=\exp\{A(t)+B(t)\eta_{t}+C(t)\lambda_{t}+D(t)\lambda_{t}^{2}\}$ and let $Y_{t}=\int_{u_{1}}^{t}1_{\left[u_{2},u_{3}\right]}(s,dz_{s}$. Here *B*,*C*,*D* are the chained coefficients evaluated in backward time, with the regime integral included in *A*. Set $M_{t}=e^{pY_{t}}F_{t}$ and $p_{t}=p1_{\left[u_{2},u_{3}\right]}(t)$. The terminal value is $M_{u_{3}}=e^{pY}$. The scalar comparisons in (A.4) bound *B*,*D* above on the finite horizon; their Riccati equations bound them below on finite intervals, and the remaining linear equation bounds *C*. Thus these deterministic coefficients are bounded, including before *u*_2_, where $p_{t}=0$ but *B*,*C*,*D* retain their terminal coupling. Itô’s formula gives $M_{t}/M_{u_{1}}=E(L{)}_{t}$, with


$$\begin{array}{c} dL_{t}=p_{t}\sqrt{\eta_{t}}\;d{\tilde{W}}_{t}^{1}+\omega B(t)\sqrt{\eta_{t}}\;d{\tilde{W}}_{t}^{2}+p_{t}\sigma \lambda_{t}\;d{\tilde{W}}_{t}^{3}+\beta (C(t)+2D(t)\lambda_{t})\;d{\tilde{W}}_{t}^{4}. \end{array}$$

Stop at $\tau_{n}=\inf\{t\geq u_{1}:1+\eta_{t}+\lambda_{t}^{2}\geq n\}$ and use the density $E(L{)}_{u_{3}\wedge \tau_{n}}$ to define ${\tilde{\mathbb{Q}}}_{n}$. Its quadratic-variation rate is bounded before $\tau_{n}$, so this stopped density is a true martingale. Using precisely the correlations in Assumption 1, the tilted drifts before $\tau_{n}$ are


$$\begin{array}{cc} {\tilde{b}}_{\eta } & =\epsilon {\tilde{\delta }}_{X_{t}}+\epsilon \chi \lambda^{2}+(-\epsilon +p_{t}\rho_{1}\omega +\omega^{2}B(t))\eta ,\\ {\tilde{b}}_{\lambda } & =\tilde{\gamma }\tilde{\alpha }+\beta^{2}C(t)+(-\tilde{\gamma }+p_{t}\rho_{2}\sigma \beta +2\beta^{2}D(t))\lambda . \end{array}$$

There is no extra mixed-state term because ${\tilde{W}}^{2}$ and ${\tilde{W}}^{4}$ have zero correlation. For $V=1+\eta +\lambda^{2}$, these drifts and $2|\lambda |\leq 1+\lambda^{2}$ imply ${\tilde{L}}_{t}V\leq C_{T}V$, with $C_{T}$ independent of *n* and of the chain path. Gronwall’s inequality gives


$$\begin{array}{c} {\tilde{𝔼}}_{n}[V_{t\wedge \tau_{n}}]\leq V_{u_{1}}e^{C_{T}(t-u_{1})},\;{\tilde{\mathbb{Q}}}_{n}(\tau_{n}\leq u_{3})\leq \frac{V_{u_{1}}e^{C_{T}(u_{3}-u_{1})}}{n}⟶0. \end{array}$$

Hence no mass is lost in the limit: $𝔼[E(L{)}_{u_{3}}]\geq \lim_{n}{\tilde{\mathbb{Q}}}_{n}(\tau_{n}>u_{3})=1$, while the positive local-martingale property gives the reverse inequality. Therefore *M* is a true martingale on this horizon and $M_{t}=𝔼[e^{pY}|F_{t},X_{\left[u_{1},u_{3}\right]}]$. In particular its stopped values form a uniformly integrable family. This proves the real-loading identity without inferring control of state weights from return moments alone. Analytic continuation of the locally analytic Riccati representation agrees with the exponential transform throughout the interior of the moment strip; strict slack extends it to the stated boundary loadings. The return-moment bounds control that transform for every imaginary part. Finally, averaging the path factor over the finite-state chain gives the finite-state Feynman–Kac equation (27), with terminal vector $1_{N}$ and the row generator $G^{\mathbb{Q}}$. Its bounded time-dependent coefficients on a finite interval ensure existence and uniqueness. A single ordinary matrix exponential is not valid unless the relevant matrices commute. This completes the verification in addition to coefficient matching.

For the Fourier identity, first use a positive damping factor $e^{-a\psi }$, *a* > 0. The identity $\int_{0}^{\infty }e^{-a\psi }\sin(\psi Y,d\psi /\psi =\arctan(Y/a)$ permits dominated convergence, because $|(e^{Y}-1)\text{arctan}(Y/a)|\leq \pi |e^{Y}-1|/2$. Thus the formula is valid at least in its Abel-limit sense as a↓0; use of an undamped truncated quadrature is separately checked numerically. We do not infer absolute integrability at infinity merely from existence of exponential moments.

### Appendix B. Numerical settings, convergence and reproducibility

#### Exhibit settings.

All experiments use Table 1, *T* = 1, $X_{0}=e_{1}$ and $\lambda_{0}=0.20$, except for the explicitly swept parameters below. Transform production settings are DOP853 $10^{-10}/10^{-12}$ with the adaptive rule in section “Parameter settings and implementation”; the real-valued stacked historical check uses RK45 with the chain integration split at the junction. No maximum-step restriction is imposed on DOP853 within a stage. Its local dense interpolant evaluates the pre-monitoring stage at the required dates. The accepted *U* and node count are recorded for every price in results/prices.csv. Fixed-grid convergence experiments override the adaptive rule as stated in their captions. Seeds and actual MC step counts are recorded in results/monte_carlo.csv.

Table 7 lists the exhibit settings.

**Table 7:** Settings IDs used in figures and tables.

ID	Experiment and deviations from baseline
ID	Experiment and deviations from baseline
VAL	*N* = 1,4,12,52,104,252; paired MC has 300,000 paths and respectively 1000, 1000, 1008, 1040, 1040, 1008 actual steps.
CHI	χ=0,0.5,1,1.5,2 crossed with *N* = 1,2,4,12,26.
ALPHA	*N* = 12; $\tilde{\alpha }=0.05,0.15,0.30,0.45,0.60$ crossed with the five CHI values.
GAMMA	*N* = 12; $\tilde{\gamma }=0.5,1,2,3.5,5$ crossed with the five CHI values.
BETA	*N* = 12; β=0.1,0.2,0.4,0.6,0.8 crossed with the five CHI values; the heatmap uses this same discrete grid.
RHO	*N* = 12; each of $\rho_{1}$ and $\rho_{2}$ is varied separately over −0.75,−0.7,−0.4,0,0.4,0.7,0.75.
NEST	(χ,σ)=(1,0.2),(0,0.2),(1,0),(0,0) crossed with *N* = 1,2,4,12,26; the table uses *N* = 12.
TARGET	t=0,0.01,...,1; exact OU second moments and the matrix exponential of the row generator; the five CHI values. No Fourier quadrature or simulation.
REF20	χ=0; baseline parameters. The seven production rows included in the 147-row total use *N* = 1,4,12,26,52,104,150. Separately, the 15 graph-comparison points use *N* = 1,2,3,4,5,6,39,40,41,74,75,76,77,148,150; Table 6 displays the six-point subset *N* = 1,4,6,40,75,150. No predecessor raw prices or source code are assumed available.
STEP	*N* = 12; requested steps 500, 1000, 2000 become 504, 1008, 2004; fixed seed 20260626, 150,000 independent pairs.

#### Moment bounds and implementation checks.

The analytic sufficient tests in Appendix A. Explicit finite-horizon bounds and transform verification pass for all 147 production rows in results/prices.csv. These represent 81 distinct model-parameter sets and 115 distinct pricing cases when *T* and *N* are included; 32 rows repeat a pricing case across exhibits. Table 8 includes the previously unlisted 20 NEST rows and seven REF20 rows, so its total is 147 rather than 120. Row identifiers, parameter identifiers, case identifiers and all certificate values are retained in results/revision_audit/certificate_rows.csv. The 15 graph-coordinate comparisons are recorded separately; they share the already certified no-feedback parameter set, and their monitoring frequencies do not change the full-horizon sufficient bound. At baseline the return-strip envelope gives $B_{*}=0.733941$ and *D*(*T*) = 0.252662; the Novikov envelope gives $B_{N}=0.316377$ and $D_{N}(T)=0.107494$. These tests cover every monitoring interval and every real Fourier frequency on the stated strip, unlike a check of selected numerical nodes. The Feller lower-bound condition is also satisfied: $2\epsilon \min_{i}{\tilde{\delta }}_{i}=0.16>\omega^{2}=0.0196$.

**Table 8:** Finite-horizon sufficient-condition checks for all 147 production rows, including repeated cases across exhibits; *T* = 1, strip [−0.1,2.1], $\nu =\tilde{\gamma }/2$. Rows count recorded runs, not distinct model parameters.

Setting	Rows	Pass	maxB	maxD
VAL	6	6	0.734	0.253
CHI	25	25	0.734	0.501
ALPHA	25	25	0.734	0.501
GAMMA	25	25	0.734	1.207
BETA	25	25	0.734	0.584
RHO $\rho_{1}$	7	7	0.804	0.276
RHO $\rho_{2}$	7	7	0.734	0.272
NEST full	5	5	0.734	0.253
NEST no feedback	5	5	0.734	0.015
NEST no direct	5	5	0.734	0.237
NEST neither	5	5	0.734	0.000
REF20	7	7	0.734	0.015
Total	147	147	–	–

The identities $K_{\chi }(0)=1$ and $K_{\chi }(-j)=e^{rT/N}$ are only code sanity checks. Separate checks compare the transform with a lognormal closed-form limit and compare the vectorized implementation with the independently integrated, real/imaginary-stacked historical equations after correcting the junction treatment. The largest absolute transform discrepancy in the latter check is 3.18e-13. Rescaling the liquidity state by *c* = 5 and transforming all coupled parameters changes the tested variance strikes by at most 3.91e-09 and the absolute-return strikes by at most 1.42e-14. The corresponding normalized dynamics are identical; these residuals are numerical error.

#### ODE and Fourier convergence.

Table 9 gives the corresponding diagnostic.

**Table 9:** ODE convergence: *N* = 12, *T* = 1, DOP853, atol = rtol/100, *U* = 160, 256 Gauss–Legendre nodes.

rtol	*P_A_*	Abs. diff.	*P_O_*	Abs. diff.
1e-07	1619.9987715	4.22e-07	39.7750554	7.36e-11
1e-08	1619.9987711	2.69e-08	39.7750554	3.08e-12
1e-09	1619.9987710	4.78e-09	39.7750554	1.49e-13
1e-10	1619.9987710	3.24e-10	39.7750554	1.42e-13
1e-11	1619.9987710	7.73e-12	39.7750554	0.00e + 00
1e-12	1619.9987710	0.00e + 00	39.7750554	0.00e + 00

Fig 12 gives the corresponding diagnostic.

**Fig 12:**
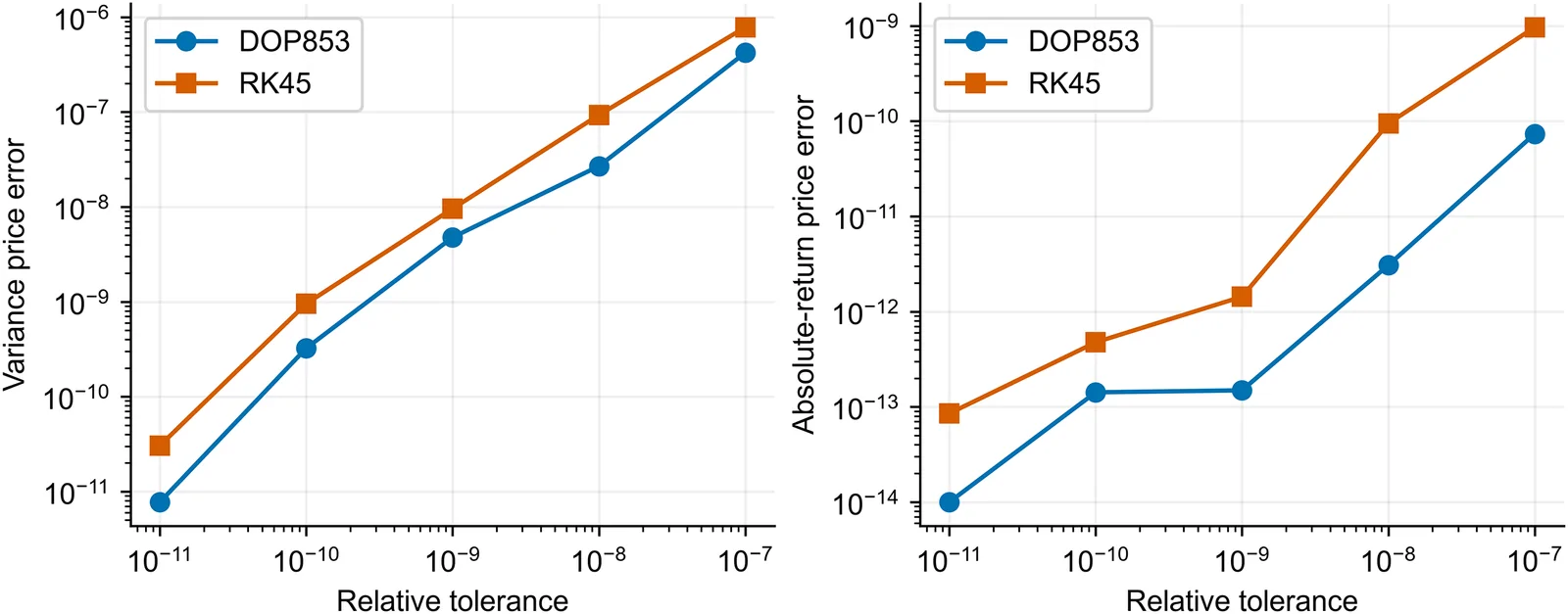
Split-stage ODE convergence against each solver’s $10^{-12}/10^{-14}$ reference. Differences below 10^–14^ are placed at that plotting floor.

Table 10 gives the corresponding diagnostic.

**Table 10:** Fourier truncation: *N* = 52, *T* = 1, 256 Gauss–Legendre nodes, lower endpoint zero, DOP853 $10^{-10}/10^{-12}$.

*U*	$P_{O}$	Difference from *U* = 480
40	38.4975689	1.23e + 00
80	39.7280527	3.02e-03
120	39.7310641	5.86e-06
160	39.7310700	2.30e-08
240	39.7310700	1.29e-12
480	39.7310700	0.00e + 00

Fig 13 gives the corresponding diagnostic.

**Fig 13:**
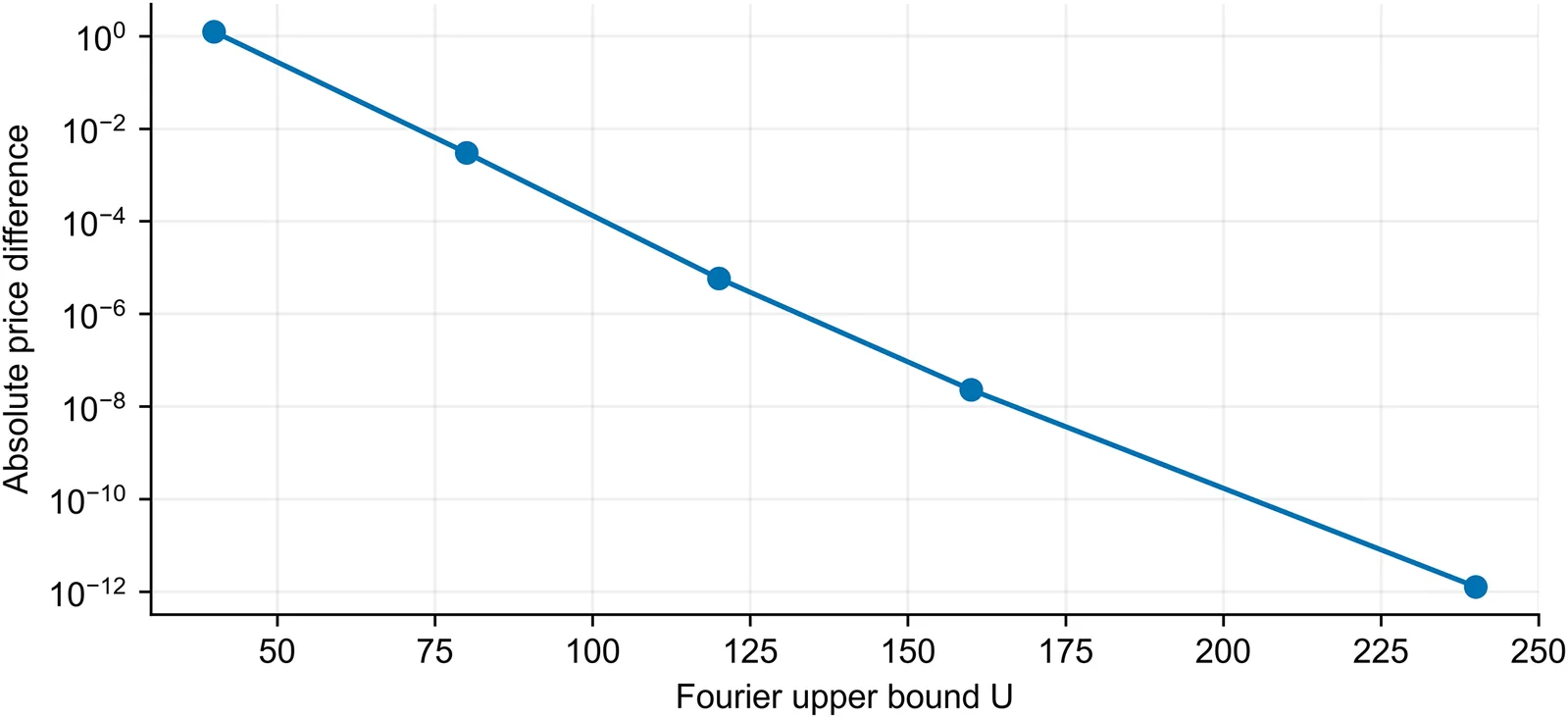
Fourier truncation at *N* = 52, with 256 nodes and *U* = 480 as the reference.

The historical tolerance values in the original table are reproduced in results/legacy_diagnosis.csv. Keeping the equations and tolerances fixed while splitting only the chain integration removes the large discrepancies. The revised production values are also compared with tighter DOP853 and RK45 solutions. Printed four-decimal production prices are supported by these checks; the additional digits in diagnostic tables are retained to expose numerical differences, not to claim exact arithmetic.

The node study uses 64, 128 and 256 points at every displayed fixed upper bound; its full output is in results/fourier_convergence.csv. A lower-cutoff experiment at *N* = 52, *U* = 300, 256 nodes gives price reductions of approximately $1.78\times 10^{-8}$, $1.78\times 10^{-6}$ and $1.78\times 10^{-4}$ when omitting (0,10^–8^), (0,10^–6^) and (0,10^–4^), respectively. Production integrates from zero and therefore introduces none of these omitted-interval biases. Gauss–Legendre nodes lie strictly inside the interval. The domain and grid tests concern numerical accuracy and are separate from the probabilistic bounds.

#### Monte Carlo time steps.

Table 11 gives the corresponding diagnostic.

**Table 11:** Time-step diagnostic: *T* = 1, 300,000 paths (150,000 independent antithetic pairs); confidence intervals use pair means. Variance leg.

*N*	Steps	$P_{A}$	MC mean	95% interval
12	504	1619.9988	1621.67	[1618.23, 1625.12]
12	1008	1619.9988	1619.93	[1616.49, 1623.36]
12	2004	1619.9988	1619.16	[1615.73, 1622.59]

Table 12 gives the corresponding diagnostic.

**Table 12:** Time-step diagnostic: absolute-return leg, same settings and paired confidence intervals.

*N*	Steps	$P_{O}$	MC mean	95% interval
12	504	39.7751	39.7844	[39.7404, 39.8284]
12	1008	39.7751	39.7838	[39.7399, 39.8276]
12	2004	39.7751	39.7645	[39.7206, 39.8084]

These are independently discretized simulations with a fixed seed, not coupled nested-grid estimators of a bias difference. Their overlap supports numerical consistency at the stated sampling precision; it does not identify the exact discretization bias. The actual step counts, rather than requested rounded counts, determine Δt.

#### Computational cost.

The separate timing run uses *N* = 252, *T* = 1, Python 3.12.10, NumPy 2.4.6 and SciPy 1.17.1 on Windows 11 with an Intel Core Ultra 5 338H, with numerical-library threads set to one. The transform timings include both legs, node refinement and domain checks, and exclude only module import. Monte Carlo timings include path generation and paired uncertainty calculation. A common empirical accuracy target is 2 variance quotation units and 0.03 absolute-return quotation units. This is a comparison at specified practical tolerances, not a claim of optimal implementation or a rigorous matched total-error bound. The median of three transform runs is 0.1754 seconds. The 300,000-path Monte Carlo run with 1008 steps takes 47.19 seconds, with 95% half-widths 0.8722 and 0.01072; its prices are 1606.4199 and 39.72101. The 300,000-path Monte Carlo run with 2016 steps takes 92.55 seconds, with 95% half-widths 0.8705 and 0.01069; its prices are 1606.5209 and 39.71982. Both simulation results and interval widths are reported so that the accuracy comparison can be assessed directly. The finer-grid run probes remaining time-discretization sensitivity; the sampling uncertainty prevents certification of a much tighter total bias. Raw times and environment details are supplied, without extrapolating them to other hardware.
