# Supplementary material for: Variance and volatility swap valuation with nonlinear liquidity-feedback stochastic volatility and regime switching
Source: PLoS One. 2026 Oct 27;21(10):e0355147. doi: 10.1371/journal.pone.0355147 (PMC13712705; doi:10.1371/journal.pone.0355147)
Supplement: Supplementary file 1 [file pone.0355147.s001.pdf]

Reviewer Report

Manuscript: "Variance and Volatility Swap Valuation with Nonlinear Liquidity-Feedback Stochastic Volatility and Regime Switching"

Review framework: the manuscript is assessed against the seven publication criteria specified by the journal (originality; no prior publication; technical standard and sufficiency of description; conclusions supported by the results; intelligibility and standard English; ethics and research integrity; reporting guidelines and data availability).

Summary

Criterion	Reviewer opinion
1. Original research	Met, with reservations on the size of the increment.
2. Results not published elsewhere	Cannot be verified as written; requires explicit delineation from He, et al. (2025) (reference [20] of the paper).
3. Technical standard and sufficient detail	Partially met — derivations verified correct, but proofs are formal and the numerical reporting is internally inconsistent.
4. Conclusions supported by the results	Partially met — core comparative statics supported; several specific claims overstated or tautological.
5. Intelligible, standard English	Met, subject to copy-editing (redundancy, typographic defects).
6. Ethics and research integrity	Met, conditional on transparent disclosure of the relationship to [20].
7. Reporting guidelines and data availability	Not met — computational study without code deposit or complete settings.

Recommendation: Major revision.

Manuscript summary

The paper prices discretely monitored variance and volatility swaps in a hybrid risk-neutral model with three ingredients: (i) an N-state continuous-time Markov chain X switching the long-run variance target $\tilde{\delta}_x$ (two states in the numerics); (ii) a Heston-type square-root variance process η with vol-of-vol ω; and (iii) a signed Gaussian Ornstein–Uhlenbeck "liquidity-stress" factor λ entering twice — contemporaneously in returns through $\sigma\lambda\, dW^3$, and with a lag through the variance drift, whose effective target is $\tilde{\delta}_x + \chi\lambda^2$. Because λ has linear drift and constant diffusion, the augmented state $(z, \eta, \lambda, \lambda^2)$ remains inside the exponential linear–quadratic transform class, and the authors derive a forward characteristic function of log-return increments from two chained Riccati ODE

systems (Proposition 2, eqs. (17) – (25)) and a finite-dimensional Markov-chain backward ODE (27). Variance-swap strikes follow by evaluating the transform at $\psi \in \{-j, -2j\}$ (Corollary 1); volatility-swap strikes from the sign-function Fourier representation of $E|e^y - 1|$ (Corollary 2). Section 4 validates against Monte Carlo, reports truncation and tolerance diagnostics, and decomposes prices across nested benchmarks ($\chi = 0$, $\sigma = 0$), concluding that the delayed feedback channel dominates the direct return–liquidity channel at the baseline parameterization.

Criterion 1. The study presents the results of original research

Met, with reservations.

The specific combination — regime-switching variance target, Heston variance, and a signed Ornstein-Uhlenbeck (OU) liquidity factor with a quadratic feedback term $\chi\lambda^2$ in the variance drift — has not, to my knowledge, been solved in closed transform form before, and the two-stage forward characteristic function for discretely monitored swaps in this model is a genuine derivation. I verified it independently: the coefficient matching in the Riccati systems (17) – (24), the terminal chaining (25), the piecewise source (26), the Feynman–Kac representation (27), and the constant algebra in Corollary 2 (the $2/\pi$ identity combined with $m\sqrt{(\pi/(2NT))}$ to give $m\sqrt{(2/(\pi NT))}$) are all correct. In that narrow sense the work is original.

Two reservations bound the size of the contribution, and the manuscript should state them itself rather than leave them to the reader. First, the tractability device is the quadratic-Gaussian / linear–quadratic transform class, standard since Ahn–Dittmar–Gallant (2002), Leippold–Wu (2002) and Cheng–Scaillet (2007), none of which is cited; the derivation is coefficient matching within a known class, and the label “nonlinear” overstates what is, in the augmented state, a linear–quadratic model. Second, relative to reference [20] — whose title differs from the present one essentially by the phrase “nonlinear liquidity feedback” and which appears to be the $\chi = 0$ backbone of this model — the increment is the single term $\chi\lambda^2$ and its induced forcings ($\kappa\chi\xi_2$, $\kappa\chi\xi_6$) in the Riccati systems. Originality also carries an unexercised opportunity cost: a fully affine alternative (Cox-Ingersoll-Ross (CIR) liquidity intensity ℓ, loading $\sigma\sqrt{\ell}$, drift term $\chi\ell$) would deliver both channels with a nonnegative intensity and no quadratic extension; the paper should explain what the signed-Gaussian specification buys, especially since its own Fig. 9 shows the distinctive ρ_2 channel is nearly flat.

Criterion 2. Results reported have not been published elsewhere

Cannot be verified as written; explicit delineation required.

I have no evidence of duplicate publication, but the overlap with reference [20] is close enough — in title, model structure, contract set, and methodology — that the manuscript must make the boundary explicit. Three requirements: (a) A dedicated paragraph stating precisely what is inherited from [20] (model backbone, transform architecture, numerical scheme) and what is new (the feedback term, the associated Riccati forcings, the benchmark decomposition). (b) A quantitative reconciliation: the nested $\chi = 0$ specification should reproduce the numbers published in [20], which would simultaneously provide the external code validation the paper currently lacks. (c) If any text, figures, or derivations are shared with [20] or with other work by the authors, this must be disclosed to the

editor. As it stands, a reader cannot determine from the manuscript alone whether the results at $\chi = 0$ — which anchor every decomposition in Section 4 — are new computations or republication.

Criterion 3. Analyses performed to a high technical standard and described in sufficient detail

Partially met. This is where most of the required revision lies.

What meets the standard. The analytical core is correct (see Criterion 1). The internal arithmetic of the numerical study is fully consistent: in Table 6 the nested differences reproduce each other exactly (324.2786 + 39.2011 = 39.2877 + 324.1920 = 363.4797 for the variance swap), and the contribution ratios in Table 7 follow from Table 6 without discrepancy. The Monte Carlo validation in Table 2 is genuine evidence: semi-analytical prices fall inside the 95% intervals at every monitoring frequency.

Where the mathematical standard falls short. The proofs assume precisely what is hard. Assumption 3 is the Novikov condition, so Proposition 1 is immediate and the substantive question — primitive parameter restrictions guaranteeing (8) — is left open. Assumption 4 assumes finiteness of the transform at $\psi = -2j$ and on the shifted strip, exactly the moment-explosion territory mapped by Andersen–Piterbarg (2007) and Glasserman–Kim (2010). Since these functionals are finite iff Riccati systems of the very type the paper solves do not explode, checkable sufficient conditions are within reach and should be supplied. For example: at $\psi = -2j$ the η-block is autonomous, $\xi_2' = \frac{1}{2}\omega^2\xi_2^2 + (2\rho_1\omega - \kappa)\xi_2 + 1$ with $\xi_2(0) = 0$; if $(\kappa - 2\rho_1\omega)^2 > 2\omega^2$ (baseline: $1.30 > 0.04$) the quadratic has two positive real roots and ξ_2 rises monotonically to the smaller one, hence is bounded for every horizon; a comparison argument then bounds the λ-block given the ξ_2 bound. Closed-form thresholds for the building blocks (Cameron–Martin: exponential moments of $\int\lambda^2$ up to $\tilde{\gamma}^2/(2\beta^2)$; CIR: $\int\eta$ up to $\kappa^2/(2\omega^2)$) belong in the paper. Separately, Proposition 2 is established by coefficient matching only; a verification argument is needed (candidate solves the backward equation; local-martingale-plus-integrability identifies it with the conditional expectation under Assumption 4), along with an explicit proof of the conditioning-on-the-chain-path step behind the factorization into $M(u_1)$. Lemma 1 should cite a comparison theorem valid for random drifts (Ikeda–Watanabe; Karatzas–Shreve Prop. 5.2.18).

Where the numerical description is insufficient or internally inconsistent. All of the following are verifiable from the paper's own tables. (a) The headline variance strike in Tables 2 and 6 ($N = 12$: 1620.0538) matches the rtol = 10^{-8} row of Table 5 to every printed digit (1620.053829), and Table 5 itself shows this tolerance is off by 5.5×10^{-2} from the rtol = 10^{-10} benchmark (1619.998354): the headline numbers were produced at a tolerance the paper's own diagnostics deem insufficient, and are printed to four decimals anyway, while §4.6 recommends the tighter tolerance "for final production runs" without applying it. (b) Table 5's error is non-monotone in rtol (5.447×10^{-2} at 10^{-7} versus 5.547×10^{-2} at 10^{-8}); unexplained, this undermines the convergence claim. (c) The diagnostics were run at different, undisclosed monitoring frequencies: Converged volatility strike (39.731069) of Table 4 matches $N = 52$ row of Table 2, while the reference (39.775055) of Table 5 matches $N = 12$; neither table states N or T. (d) The claim in §3.2 that "$U = 50 - 100$ is typically adequate" is contradicted by Table 4 ($U = 40$ off by 1.2335, ≈3% of the strike; $U = 120$ needed for 10^{-5}); a fixed U

is not meaningful in any case, since the required truncation grows with monitoring frequency roughly like $\sqrt{(N/T)}$ — an adaptive rule tied to the per-interval variance should replace it. (e) The maturity T is never stated (evidently 1, inferred from the frequency axis) and the initial regime X_0 is never stated (evidently e_1, inferred from Fig. 14's intercepts 0.08 and $0.08 + \chi\lambda_0^2 = 0.12$); both belong in Table 1, along with a unified generator notation (v_{12}/v_{21} in the table versus g_{ij} in eq. (1)). Every table should disclose its full settings: N, T, quadrature rule and ψ-grid, lower cutoff ε and its bias, solver, rtol, atol. Finally, the advertised "martingale admissibility check" is not a check at all — see Criterion 4.

Criterion 4. Conclusions are presented appropriately and supported by the data

Partially met.

The central qualitative conclusions are supported by the reported results: the feedback channel dominates the direct channel at baseline (Table 6), variance swaps respond more strongly than volatility swaps (consistent across Tables 6–7 and Figs. 4–8), the $\tilde{\gamma}$ non-monotonicity follows correctly from the mean-versus-dispersion decomposition of $E[\lambda^2]$, and the near-linearity of the contribution ratio in χ is visible in Table 7. One clean supported finding is present in the numbers but never stated and should be: the two channels are almost exactly additive at baseline (cross-channel interaction +0.0866 out of 363.48 for the variance swap; −0.0921 out of 4.66 for the volatility swap), so the interesting nonlinearities are within the feedback channel, not across channels.

Four claims are not supported as presented. (a) The "martingale admissibility check" $K(-j) = e^{rT}$ (abstract, §3.1, §4.2) is tautological: at $\psi = -j$ one has $j\psi + \psi^2 = 0$, so every Riccati source vanishes; with zero initial conditions all ξ's vanish identically, $b \equiv 0$, $F \equiv 1$ because the generator's rows sum to zero, and $\bar{\xi}_1(\tau_2) = r\tau_2$ — hence $K(-j) = e^{rT}$ for any parameters whatsoever, martingale or not (likewise $K(0) = 1$). These are code tests and must be relabeled as such; presenting them as evidence of martingale validity is a claim the mathematics does not support. (b) The reported precision (four decimals) exceeds the demonstrated accuracy (5.5×10^{-2}; Criterion 3). (c) §4.5 characterizes the ρ_1 channel as "significant" when the full sweep $\rho_1 \in [-0.75, 0.75]$ moves the strike by roughly 1.5%, against a 25% feedback contribution at $\chi = 1$; magnitudes should be quantified, not characterized. (d) The "risk premium" language in §4.3 is undefined in a Q-only model with no specified market prices of risk; either introduce the P-measure link or remove the interpretation. Additionally, all economic conclusions should be explicitly flagged as conditional on an uncalibrated parameterization — the paper says the baseline is "not intended to calibrate or represent any specific market data," and the abstract and conclusion should carry that qualification rather than bury it in §4.1.

Criterion 5. The article is intelligible and written in standard English

Met, subject to copy-editing.

The English is grammatical and the technical exposition is generally clear. Three defects need attention. First, redundancy: the two-channel mechanism paragraph appears nearly verbatim twice in the Introduction, and the variance-versus-volatility sensitivity explanation is repeated in §§4.3, 4.4, 4.5, 4.6, 4.8 and the Conclusion; Fig. 1 duplicates Figs. 2–3 exactly; Tables 3–5 and Figs. 11–12

are diagnostics best moved to an appendix. Second, a typographic defect: in the copy reviewed, en/em-dashes are dropped in many compounds (“returnliquidity”, “liquiditystress”, “pricevariance”, “designnamely” on pp. 15, 17, 19, 24) while the same compounds are correctly hyphenated elsewhere (p. 4) — evidently a character-encoding problem in the source that must be fixed globally; check also the “U = 50–100” range punctuation in §3.2 and the capitalization of “Fourier” in reference [5]. Third, smaller items: §4.4 calls $E[\lambda^2] = Var(\lambda) + (E[\lambda])^2$ a “qualitative approximation” when it is an exact identity with closed-form components for an OU process; m = 100 is a quoting convention, not an “annualization scaling factor”; the superscript in K^χ collides with the parameter χ; Assumption 1’s independence statement should say plainly that X is independent of $(W^1, \ldots, W^4)$; the N = 1 case is not a traded contract design and should be labeled a stress case; and several figures rely on red/green contrasts (Figs. 4, 6–8, 10) that are not colorblind-safe, with partially illegible legends in Figs. 5 and 13.

Criterion 6. Ethics of experimentation and research integrity

Met, conditional on disclosure.

The study involves no human or animal subjects and no personal data, so experimental-ethics requirements do not apply. Two integrity-adjacent points require action, neither of which I raise as an accusation. First, the proximity to [20] (Criterion 2) must be transparently disclosed to the editor and delineated in the text; with a near-identical title and a nested model. Second, reporting numbers to a precision the authors’ own diagnostics show is not attained (Criterion 3), and labeling a tautological identity a “martingale admissibility check” (Criterion 4), are reporting practices that overstate the evidence; both must be corrected in revision. Reference attribution also needs care: the sign-function representation of $E|e^y - 1|$ and the two-stage forward-transform construction read as if introduced here and should be attributed to their sources (the Zheng and Kwok 2014 / Elliott and Lian 2013 lineage; Zhu and Lian 2015; Broadie and Jain 2008 for discrete monitoring).

Criterion 7. Reporting guidelines and community standards for data availability

Not met.

The Data Availability Statement asserts that the data supporting the findings “are available from the corresponding author upon reasonable request.” This fails the criterion twice over. First, it is category-confused: the study uses no data — every finding is generated by code from the parameterization in Table 1 — so nothing answers to the description “data that support the findings”; the artifact that determines reproducibility is the code, together with the complete numerical settings, and the statement is silent on both. Second, even taken at face value, availability upon request from the corresponding author does not meet contemporary community standards for computational work: it makes reproducibility contingent on the authors’ discretion and continued reachability, cannot be verified at review, and is the practice that data-availability policies of this journal’s type were written to replace — repository or supplementary-file deposit is the norm, with author-mediated access reserved for legally or ethically restricted material, of which there is none here. Community standards for computational finance therefore require: (a) deposit of the pricing

and Monte Carlo code (the paper states seeds are fixed, which is only meaningful if the code is available); (b) a complete settings table per numerical exhibit — N, T, X_0, ψ-quadrature and grid, truncation U, lower cutoff ε, solver, rtol, atol — none of which is currently complete (Criterion 3); and (c) a computational-cost report: the semi-analytical route requires two complex Riccati systems plus the chain ODE on a ψ-grid per monitoring date, and a timing comparison against Monte Carlo at matched accuracy is the relevant evidence that the method adds value at, say, N = 252. Item (b) is not optional: at present the headline tables cannot be reproduced from the manuscript alone, since T and X_0 must be reverse-engineered from figure intercepts.

Revision requirements:

Major comments:

1. Delineate and reconcile with [20]: inheritance paragraph, disclosure to the editor, and quantitative reproduction of published numbers at $\chi = 0$ of [20]. (Criteria 1, 2, 6)
2. Recompute headline tables at rtol $\le 10^{-9}$ or round to demonstrated accuracy; explain the non-monotone rtol errors; disclose full settings per table; reconcile §3.2's truncation guidance with Table 4 via an adaptive rule; add T and X_0 to Table 1. (Criteria 3, 4, 7)
3. Replace Assumptions 3 and 4 with checkable sufficient conditions at the arguments used (0, −j, −2j, the strip), citing Andersen–Piterbarg and Glasserman–Kim; add a verification argument to Proposition 2 and the conditioning step for the chain; cite a precise comparison theorem in Lemma 1. (Criterion 3)
4. Relabel $K(0) = 1$ and $K(-j) = e^{rT}$ as code sanity checks; remove "martingale admissibility" claims tied to them. (Criteria 4, 6)
5. Position the model within the linear–quadratic transform literature; justify the signed-Gaussian specification against the affine CIR-intensity alternative; state the cost of the zero W^2–W^4 correlation; address the scale indeterminacy $(\lambda, \tilde{\alpha}, \beta, \lambda_0) \to c(\cdot)$, $\sigma \to \sigma/c$, $\chi \to \chi/c^2$ by imposing an explicit normalization. (Criteria 1, 3)
6. Report the channel-additivity finding; quantify the ρ_1 effect; remove or ground the risk-premium language; qualify all economic conclusions as conditional on an uncalibrated parameterization. (Criterion 4)
7. Replace the "available upon reasonable request" Data Availability Statement with a code-availability statement and public repository deposit (code with fixed seeds and per-table settings); add a timing comparison versus Monte Carlo. (Criterion 7)

Minor comments:

1. Cut redundancy, fix the dash-encoding defect globally, correct the minor items under Criterion 5, and adopt a colorblind-safe palette. (Criterion 5)

Recommendation

Major revision. Under a soundness-based rubric, the manuscript's core is salvageable: the derivations are correct — I verified them independently — and the numerical study, once its settings are disclosed and its precision honestly stated, is a legitimate validation of a legitimate (if incremental) model. The failures are concentrated in verifiability rather than in the mathematics: undisclosed numerical settings, precision beyond demonstrated accuracy, assumptions that assume the theorems' hard parts, a mislabeled tautological check, an unclarified relationship to a near-identical predecessor paper, and no code deposit. Every one of these is fixable, and criteria 2, 3, 4 and 7 should be re-examined strictly on resubmission.

References:

Ahn, D.-H., Dittmar, R. F., & Gallant, A. R. (2002). Quadratic term structure models: Theory and evidence. *The Review of Financial Studies, 15*(1), 243–288. https://doi.org/10.1093/rfs/15.1.243

Elliott, R. J., & Lian, G.-H. (2013). Pricing variance and volatility swaps in a stochastic volatility model with regime switching: Discrete observations case. *Quantitative Finance, 13*(5), 687–698. https://doi.org/10.1080/14697688.2012.676208

Leif B. G. Andersen and Vladimir V. Piterbarg (2007). Moment Explosions in Stochastic Volatility Models. *Finance and Stochastics*, Vol. 11, pp. 29–50 (2007). https://doi.org/10.1007/s00780-006-0011-7

Broadie, Mark and Jain, Ashish (2008). The Effect of Jumps and Discrete Sampling on Volatility and Variance Swaps. *International Journal of Theoretical and Applied Finance*, 11(8), 761–797. https://doi.org/10.1142/S0219024908005032

Cheng, P., & Scaillet, O. (2007). Linear-quadratic jump-diffusion modeling. *Mathematical Finance, 17*(4), 575–598. https://doi.org/10.1111/j.1467-9965.2007.00316.x

Glasserman, P., & Kim, K.-K. (2010). Moment explosions and stationary distributions in affine diffusion models. Mathematical Finance, 20(1), 1–33. https://doi.org/10.1111/j.1467-9965.2009.00387.x

X.-J. He, W. Chen, T. Lu, S. Lin (2025). Variance and volatility swap valuation with stochastic liquidity and regime switching stochastic volatility. *Communications in Nonlinear Science and Numerical Simulation* 154 (2025) 109558.

W. Zheng, Y. K. Kwok (2014). Closed form pricing formulas for discretely sampled generalized variance swaps, *Mathematical Finance* 24, 855–881. https://doi.org/10.1111/mafi.12016

Leippold, M., & Wu, L. (2002). Asset pricing under the quadratic class. *Journal of Financial and Quantitative Analysis, 37*(2), 271–295. https://doi.org/10.2307/3595006

Song-Ping Zhu and Guang-Hua Lian (2015). Analytically Pricing Volatility Swaps under Stochastic Volatility. *Journal of Computational and Applied Mathematics*, 288, 332–340. https://doi.org/10.1016/j.cam.2015.04.036

W. Zheng, Y. K. Kwok (2014). Closed form pricing formulas for discretely sampled generalized variance swaps, *Mathematical Finance* 24, 855–881. https://doi.org/10.1111/mafi.12016
