# Supplementary material for: Variance and volatility swap valuation with nonlinear liquidity-feedback stochastic volatility and regime switching
Source: PLoS One. 2026 Oct 27;21(10):e0355147. doi: 10.1371/journal.pone.0355147 (PMC13712705; doi:10.1371/journal.pone.0355147)
Supplement: Supplementary file 2 [file pone.0355147.s002.pdf]

Reviewer Report on

"Variance and Volatility Swap Valuation with Nonlinear Liquidity–Feedback Stochastic Volatility and Regime Switching" (PONE-D-26-35832)

by Jing Fu and Ke Wang

September 2026

In this paper the authors present a nonlinear liquidity-feedback regime-switching stochastic volatility model for the pricing of discretely sampled variance and volatility swaps, where the core innovation lies in the introduction of a quadratic feedback channel to transmit stochastic liquidity stress into future variance dynamics while ensuring that signed stress deviations make a nonnegative contribution to the future variance target — thereby enriching the descriptive capacity of the hybrid stochastic framework. By solving the Riccati-type deterministic ordinary differential equation systems and the finite-state Markov-chain backward equation, the authors derive a semi-analytical forward characteristic function and then obtain pricing formulas for discretely monitored variance and volatility swaps. The authors also conduct numerical analysis based on Monte Carlo confidence intervals and various convergence tests to support the validity of the pricing formulas and check the effectiveness of their implementation. The results show the collective role of feedback strength, the long-run signed liquidity-stress mean, liquidity-stress persistence, liquidity-stress volatility, and the correlation parameters collectively in determining volatility-risk valuation.

Overall, I think that the paper offers a meaningful contribution to the stochastic volatility literature by introducing a valid framework that incorporates stochastic liquidity and its interaction with volatility in the context of pricing variance and volatility swaps. That said, beyond minor errors, the proposed framework has several notable limitations, and numerous aspects of the presentation would benefit from greater clarity, and accordingly I encourage the authors to carefully address the following comments in their revision.

1. The abstract is too detailed and contains more information than necessary, including the sentence on p.1 ll.17-19. It should be revised to be more concise and focused.

2. p.2 ll.-15--16: The literature review on variance and volatility swap valuation under stochastic volatility should be expanded to include recent studies – in particular those on rough volatility models, which are compatible with Fourier-based pricing techniques presented; see in particular [1, 2, 3]. This addition will also provide a useful contrast with the proposed framework and highlight possible avenues for integration and future extensions.

3. p.3 l.6: Throughout the manuscript, there are many missing hyphens within word clusters, such as in "liquidity-volatility". Please double check and make sure that they are properly displayed.

4. p.4 l.12: With Equation (4), η is no longer the instantaneous variance of returns, which is also modulated by the liquidity term $\sigma\lambda$. The authors need to adjust the interpretations accordingly.

5. p.4 l.13: Parameter χ in Equation (5) can be absorbed into parameters $\tilde{\gamma}$ and β in Equation (6), or alternatively, one of the latter two parameters can be normalized to 1. Doing so will improve the model on parametric parsimony.

6. p.4: The model involves over ten parameters in total, which makes it somewhat unparsimonious and potentially unstable when estimated or calibrated to market data. My main concern is that empirical estimation may not adequately support the level of interpretation presented in Section 4, which could

limit the model's practical applicability. Though I am not requesting a full calibration study, the authors should consider adding some remarks to help substantiate the model's real-world relevance.

7. p.4 l.24: Please state that $\delta_i > 0$ is for all i.

8. p.5 l.15: By convention, stochastic processes should be written as either X (without the subscript t) or $\{X_t\}$ (or (X_t), parenthesized) to avoid confusion with fixed-time random variables. Please consider making this change throughout the manuscript.

9. p.5 l.16: Please write "For all $t \geq 0$", and apply this language to other arguments alike.

10. p.6 l.16: Please add that z is given by Equation (15).

11. p.6 l.18: Ideally, Assumption 4 should be stated in terms of model parameters and verified a posteriori — i.e., after estimation or calibration. The authors could indicate in Section 4 whether this assumption holds in their numerical illustrations and, if not, specify the parameter ranges for which it does, along with an assessment of practical plausibility.

12. p.10 ll.11-12: The realized volatility defined in Equation (36) is not the square root of the realized variance defined in Equation (30). Please explain why. Moreover, what does the "zero-mean normal assumption" mean? Does it not contradict the model implications for the return distribution?

13. p.11 ll.14-16: Please give more details on how the Riccati system is solved numerically.

14. p.11 ll.-6--7: Even in such cases, the model should ideally be amenable to robust calibration to market data, which reinforces the earlier point regarding parametric parsimony.

15. p.12 ll.1-3: Please provide empirical justification for the parameter choices. In particular, the table assumes a significant price-liquidity leverage effect, which should be supported by empirical evidence, apart from the well-known price-volatility leverage effect. Moreover, $\epsilon = 1.00$ appears somewhat low for a variance process characterized by fast mean reversion.

16. p.18 l.-5: Again, does varying these parameter values (in particular when in the positive range) affect the integrability condition in Proposition 2?

17. Despite that the paper focuses on swaps, it naturally raises the question of how the framework applies to volatility options. Specifically, the semi-explicit tractability of the model stems from the linear structure of variance swaps, which allows the characteristic function of each component to be treated separately via interchange of expectation and summation. As a result, this aspect is mathematically expected and unsurprising. For nonlinear payoffs, however, the characteristic function of the realized variance would need to be treated jointly due to the complex temporal dependence structure, and the realized volatility can be interpreted as its square root. In this case, the pricing of volatility options relies either on simulation techniques (Section 3.2 of [1]) or on a modified Fourier transform-type formula (Proposition 5 in [3]). Therefore, a discussion along these lines would be a valuable addition to the paper.

References

[1] Horvath, B., Jacquier, A., & Tankov, P. (2020). Volatility options in rough volatility models. SIAM Journal on Financial Mathematics, 11(2), 437-469.

[2] Salmon, N., & SenGupta, I. (2021). Fractional Barndorff-Nielsen and Shephard model: Applications in variance and volatility swaps, and hedging. Annals of Finance, 17(4), 529-558.

[3] Wang, L., & Xia, W. (2022). Power-type derivatives for rough volatility with jumps. Journal of Futures Markets, 42(7), 1369-1406.
