# Supplementary material for: Variance and volatility swap valuation with nonlinear liquidity-feedback stochastic volatility and regime switching
Source: PLoS One. 2026 Oct 27;21(10):e0355147. doi: 10.1371/journal.pone.0355147 (PMC13712705; doi:10.1371/journal.pone.0355147)
Supplement: Peer review history

# Peer review: Decision Letter 0

**DOI:** 10.1371/journal.pone.0355147.r001

**Authors:** Szabolcs Blazsek

9 Sep 2026

Dear Dr. Wang,

Thank you for submitting your manuscript to PLOS One. After careful consideration, we feel that it has merit but does not fully meet PLOS One’s publication criteria as it currently stands. Therefore, we invite you to submit a revised version of the manuscript that addresses the points raised during the review process.

==============================

**Please address the comments of the reviewers and resubmit the paper to PLOS One.**

==============================

Please submit your revised manuscript by Nov 08 2026 11:59PM. If you will need more time than this to complete your revisions, please reply to this message or contact the journal office at plosone@plos.org. When you're ready to submit your revision, log on to https://www.editorialmanager.com/pone/ and select the 'Submissions Needing Revision' folder to locate your manuscript file.

- A letter that responds to each point raised by the academic editor and reviewer(s). You should upload this letter as a separate file labeled 'Response to Reviewers'.
- A marked-up copy of your manuscript that highlights changes made to the original version. You should upload this as a separate file labeled 'Revised Manuscript with Track Changes'.
- An unmarked version of your revised paper without tracked changes. You should upload this as a separate file labeled 'Manuscript'.

If you would like to make changes to your financial disclosure, please include your updated statement in your cover letter. Guidelines for resubmitting your figure files are available below the reviewer comments at the end of this letter.

If applicable, we recommend that you deposit your laboratory protocols in protocols.io to enhance the reproducibility of your results. Protocols.io assigns your protocol its own identifier (DOI) so that it can be cited independently in the future. For instructions see: https://journals.plos.org/plosone/s/submission-guidelines#loc-laboratory-protocols. Additionally, PLOS One offers an option for publishing peer-reviewed Lab Protocol articles, which describe protocols hosted on protocols.io. Read more information on sharing protocols at https://plos.org/protocols?utm_medium=editorial-email&utm_source=authorletters&utm_campaign=protocols.

As the corresponding author, your ORCID iD is verified in the submission system and will appear in the published article. PLOS supports the use of ORCID, and we encourage all coauthors to register for an ORCID iD and use it as well. Please encourage your coauthors to verify their ORCID iD within the submission system before final acceptance, as unverified ORCID iDs will not appear in the published article. *Only*  the individual author can complete the verification step; PLOS staff *cannot*  verify ORCID iDs on behalf of authors.

We look forward to receiving your revised manuscript.

Kind regards,

Szabolcs Blazsek

Academic Editor

PLOS One

Journal Requirements:

When submitting your revision, we need you to address these additional requirements.

1. Please ensure that your manuscript meets PLOS ONE's style requirements, including those for file naming. The PLOS ONE style templates can be found at

https://journals.plos.org/plosone/s/file?id=wjVg/PLOSOne_formatting_sample_main_body.pdf and https://journals.plos.org/plosone/s/file?id=ba62/PLOSOne_formatting_sample_title_authors_affiliations.pdf

2. Please note that PLOS One has specific guidelines on code sharing for submissions in which author-generated code underpins the findings in the manuscript. In these cases, we expect all author-generated code to be made available without restrictions upon publication of the work. Please review our guidelines at https://journals.plos.org/plosone/s/materials-and-software-sharing#loc-sharing-code and ensure that your code is shared in a way that follows best practice and facilitates reproducibility and reuse.

3. Please provide a complete Data Availability Statement in the submission form, ensuring you include all necessary access information or a reason for why you are unable to make your data freely accessible. If your research concerns only data provided within your submission, please write "All data are in the manuscript and/or supporting information files" as your Data Availability Statement.

4. Thank you for stating the following financial disclosure:

This work was supported by the Fundamental Research Funds for the Central Universities

(grant no. 25CAFUC04077) and the Scientific Research Fund Project of Yunnan Provincial

Department of Education (grant no. 2026J1134)

Please state what role the funders took in the study. If the funders had no role, please state: "The funders had no role in study design, data collection and analysis, decision to publish, or preparation of the manuscript."

If this statement is not correct you must amend it as needed.

Please include this amended Role of Funder statement in your cover letter; we will change the online submission form on your behalf.

5. If the reviewer comments include a recommendation to cite specific previously published works, please review and evaluate these publications to determine whether they are relevant and should be cited. There is no requirement to cite these works unless the editor has indicated otherwise.

Additional Editor Comments :

Dear Authors,

We received the reports from two reviewers.

Both reviewers provided detailed reviews of the paper.

Both recommend a major revision. I agree with them.

Please carefully address all comments and resubmit the paper to PLOS ONE.

[Note: HTML markup is below. Please do not edit.]

Reviewers' comments:

Reviewer's Responses to Questions

**Comments to the Author**

1. Is the manuscript technically sound, and do the data support the conclusions?

Reviewer #1: Partly

Reviewer #2: Partly

**********

2. Has the statistical analysis been performed appropriately and rigorously?

Reviewer #1: Yes

Reviewer #2: N/A

**********

3. Have the authors made all data underlying the findings in their manuscript fully available?

The PLOS Data policy

Reviewer #1: No

Reviewer #2: No

**********

4. Is the manuscript presented in an intelligible fashion and written in standard English?

Reviewer #1: Yes

Reviewer #2: Yes

**********

Reviewer #1: Criterion 1. The study presents the results of original research

Met, with reservations.

The specific combination — regime-switching variance target, Heston variance, and a signed Ornstein-Uhlenbeck (OU) liquidity factor with a quadratic feedback term χλ² in the variance drift — has not, to my knowledge, been solved in closed transform form before, and the two-stage forward characteristic function for discretely monitored swaps in this model is a genuine derivation. I verified it independently: the coefficient matching in the Riccati systems (17) – (24), the terminal chaining (25), the piecewise source (26), the Feynman–Kac representation (27), and the constant algebra in Corollary 2 (the 2/π identity combined with m√(π/(2NT)) to give m√(2/(πNT))) are all correct. In that narrow sense the work is original.

Two reservations bound the size of the contribution, and the manuscript should state them itself rather than leave them to the reader. First, the tractability device is the quadratic-Gaussian / linear–quadratic transform class, standard since Ahn–Dittmar–Gallant (2002), Leippold–Wu (2002) and Cheng–Scaillet (2007), none of which is cited; the derivation is coefficient matching within a known class, and the label “nonlinear” overstates what is, in the augmented state, a linear–quadratic model. Second, relative to reference [20] — whose title differs from the present one essentially by the phrase “nonlinear liquidity feedback” and which appears to be the χ = 0 backbone of this model — the increment is the single term χλ² and its induced forcings (κχξ_2_, κχξ_6_) in the Riccati systems. Originality also carries an unexercised opportunity cost: a fully affine alternative (Cox-Ingersoll-Ross (CIR) liquidity intensity ℓ, loading σ√ℓ, drift term χℓ) would deliver both channels with a nonnegative intensity and no quadratic extension; the paper should explain what the signed-Gaussian specification buys, especially since its own Fig. 9 shows the distinctive ρ_2_ channel is nearly flat.

Criterion 2. Results reported have not been published elsewhere

Cannot be verified as written; explicit delineation required.

I have no evidence of duplicate publication, but the overlap with reference [20] is close enough — in title, model structure, contract set, and methodology — that the manuscript must make the boundary explicit. Three requirements: (a) A dedicated paragraph stating precisely what is inherited from [20] (model backbone, transform architecture, numerical scheme) and what is new (the feedback term, the associated Riccati forcings, the benchmark decomposition). (b) A quantitative reconciliation: the nested χ = 0 specification should reproduce the numbers published in [20], which would simultaneously provide the external code validation the paper currently lacks. (c) If any text, figures, or derivations are shared with [20] or with other work by the authors, this must be disclosed to the editor. As it stands, a reader cannot determine from the manuscript alone whether the results at χ = 0 — which anchor every decomposition in Section 4 — are new computations or republication.

Criterion 3. Analyses performed to a high technical standard and described in sufficient detail

Partially met. This is where most of the required revision lies.

What meets the standard. The analytical core is correct (see Criterion 1). The internal arithmetic of the numerical study is fully consistent: in Table 6 the nested differences reproduce each other exactly (324.2786 + 39.2011 = 39.2877 + 324.1920 = 363.4797 for the variance swap), and the contribution ratios in Table 7 follow from Table 6 without discrepancy. The Monte Carlo validation in Table 2 is genuine evidence: semi-analytical prices fall inside the 95% intervals at every monitoring frequency.

Where the mathematical standard falls short. The proofs assume precisely what is hard. Assumption 3 is the Novikov condition, so Proposition 1 is immediate and the substantive question — primitive parameter restrictions guaranteeing (8) — is left open. Assumption 4 assumes finiteness of the transform at ψ = −2j and on the shifted strip, exactly the moment-explosion territory mapped by Andersen–Piterbarg (2007) and Glasserman–Kim (2010). Since these functionals are finite iff Riccati systems of the very type the paper solves do not explode, checkable sufficient conditions are within reach and should be supplied. For example: at ψ = −2j the η-block is autonomous, ξ_2_′ = ½ω²ξ_2_² + (2ρ_1_ω − κ)ξ_2_ + 1 with ξ_2_(0) = 0; if (κ − 2ρ_1_ω)² > 2ω² (baseline: 1.30 > 0.04) the quadratic has two positive real roots and ξ_2_ rises monotonically to the smaller one, hence is bounded for every horizon; a comparison argument then bounds the λ-block given the ξ_2_ bound. Closed-form thresholds for the building blocks (Cameron–Martin: exponential moments of ∫λ² up to γ̃²/(2β²); CIR: ∫η up to κ²/(2ω²)) belong in the paper. Separately, Proposition 2 is established by coefficient matching only; a verification argument is needed (candidate solves the backward equation; local-martingale-plus-integrability identifies it with the conditional expectation under Assumption 4), along with an explicit proof of the conditioning-on-the-chain-path step behind the factorization into M(u_1_). Lemma 1 should cite a comparison theorem valid for random drifts (Ikeda–Watanabe; Karatzas–Shreve Prop. 5.2.18).

Where the numerical description is insufficient or internally inconsistent. All of the following are verifiable from the paper’s own tables. (a) The headline variance strike in Tables 2 and 6 (N = 12: 1620.0538) matches the rtol = 10^-1^ row of Table 5 to every printed digit (1620.053829), and Table 5 itself shows this tolerance is off by 5.5×10^-2^ from the rtol = 10^-10^ benchmark (1619.998354): the headline numbers were produced at a tolerance the paper’s own diagnostics deem insufficient, and are printed to four decimals anyway, while §4.6 recommends the tighter tolerance “for final production runs” without applying it. (b) Table 5’s error is non-monotone in rtol (5.447×10^-2^ at 10^-7^ versus 5.547×10^-2^ at 10^-1^); unexplained, this undermines the convergence claim. (c) The diagnostics were run at different, undisclosed monitoring frequencies: Converged volatility strike (39.731069) of Table 4 matches N = 52 row of Table 2, while the reference (39.775055) of Table 5 matches N = 12; neither table states N or T. (d) The claim in §3.2 that “U = 50 – 100 is typically adequate” is contradicted by Table 4 (U = 40 off by 1.2335, ≈3% of the strike; U = 120 needed for 10^-5^); a fixed U is not meaningful in any case, since the required truncation grows with monitoring frequency roughly like √(N/T) — an adaptive rule tied to the per-interval variance should replace it. (e) The maturity T is never stated (evidently 1, inferred from the frequency axis) and the initial regime X₀ is never stated (evidently e_1_, inferred from Fig. 14’s intercepts 0.08 and 0.08 + χλ₀² = 0.12); both belong in Table 1, along with a unified generator notation (v_1__2_/v_2__1_ in the table versus gᵢⱼ in eq. (1)). Every table should disclose its full settings: N, T, quadrature rule and ψ-grid, lower cutoff ε and its bias, solver, rtol, atol. Finally, the advertised “martingale admissibility check” is not a check at all — see Criterion 4.

Criterion 4. Conclusions are presented appropriately and supported by the data

Partially met.

The central qualitative conclusions are supported by the reported results: the feedback channel dominates the direct channel at baseline (Table 6), variance swaps respond more strongly than volatility swaps (consistent across Tables 6–7 and Figs. 4–8), the γ̃ non-monotonicity follows correctly from the mean-versus-dispersion decomposition of E[λ²], and the near-linearity of the contribution ratio in χ is visible in Table 7. One clean supported finding is present in the numbers but never stated and should be: the two channels are almost exactly additive at baseline (cross-channel interaction +0.0866 out of 363.48 for the variance swap; −0.0921 out of 4.66 for the volatility swap), so the interesting nonlinearities are within the feedback channel, not across channels.

Four claims are not supported as presented. (a) The “martingale admissibility check” K(−j) = eʳᵀ (abstract, §3.1, §4.2) is tautological: at ψ = −j one has jψ + ψ² = 0, so every Riccati source vanishes; with zero initial conditions all ξ’s vanish identically, b ≡ 0, F ≡ 1 because the generator’s rows sum to zero, and ξ̄_1_(τ_2_) = rτ_2_ — hence K(−j) = eʳᵀ for any parameters whatsoever, martingale or not (likewise K(0) = 1). These are code tests and must be relabeled as such; presenting them as evidence of martingale validity is a claim the mathematics does not support. (b) The reported precision (four decimals) exceeds the demonstrated accuracy (5.5×10^-2^; Criterion 3). (c) §4.5 characterizes the ρ_1_ channel as “significant” when the full sweep ρ_1_ ∈ [−0.75, 0.75] moves the strike by roughly 1.5%, against a 25% feedback contribution at χ = 1; magnitudes should be quantified, not characterized. (d) The “risk premium” language in §4.3 is undefined in a Q-only model with no specified market prices of risk; either introduce the P-measure link or remove the interpretation. Additionally, all economic conclusions should be explicitly flagged as conditional on an uncalibrated parameterization — the paper says the baseline is “not intended to calibrate or represent any specific market data,” and the abstract and conclusion should carry that qualification rather than bury it in §4.1.

Criterion 5. The article is intelligible and written in standard English

Met, subject to copy-editing.

The English is grammatical and the technical exposition is generally clear. Three defects need attention. First, redundancy: the two-channel mechanism paragraph appears nearly verbatim twice in the Introduction, and the variance-versus-volatility sensitivity explanation is repeated in §§4.3, 4.4, 4.5, 4.6, 4.8 and the Conclusion; Fig. 1 duplicates Figs. 2–3 exactly; Tables 3–5 and Figs. 11–12 are diagnostics best moved to an appendix. Second, a typographic defect: in the copy reviewed, en/em-dashes are dropped in many compounds (“returnliquidity”, “liquiditystress”, “pricevariance”, “designnamely” on pp. 15, 17, 19, 24) while the same compounds are correctly hyphenated elsewhere (p. 4) — evidently a character-encoding problem in the source that must be fixed globally; check also the “U = 50–100” range punctuation in §3.2 and the capitalization of “Fourier” in reference [5]. Third, smaller items: §4.4 calls E[λ²] = Var(λ) + (E[λ])² a “qualitative approximation” when it is an exact identity with closed-form components for an OU process; m = 100 is a quoting convention, not an “annualization scaling factor”; the superscript in Kᵡ collides with the parameter χ; Assumption 1’s independence statement should say plainly that X is independent of (W¹, …, W⁴); the N = 1 case is not a traded contract design and should be labeled a stress case; and several figures rely on red/green contrasts (Figs. 4, 6–8, 10) that are not colorblind-safe, with partially illegible legends in Figs. 5 and 13.

Criterion 6. Ethics of experimentation and research integrity

Met, conditional on disclosure.

The study involves no human or animal subjects and no personal data, so experimental-ethics requirements do not apply. Two integrity-adjacent points require action, neither of which I raise as an accusation. First, the proximity to [20] (Criterion 2) must be transparently disclosed to the editor and delineated in the text; with a near-identical title and a nested model. Second, reporting numbers to a precision the authors’ own diagnostics show is not attained (Criterion 3), and labeling a tautological identity a “martingale admissibility check” (Criterion 4), are reporting practices that overstate the evidence; both must be corrected in revision. Reference attribution also needs care: the sign-function representation of E|eʸ − 1| and the two-stage forward-transform construction read as if introduced here and should be attributed to their sources (the Zheng and Kwok 2014 / Elliott and Lian 2013 lineage; Zhu and Lian 2015; Broadie and Jain 2008 for discrete monitoring).

Criterion 7. Reporting guidelines and community standards for data availability

Not met.

The Data Availability Statement asserts that the data supporting the findings “are available from the corresponding author upon reasonable request.” This fails the criterion twice over. First, it is category-confused: the study uses no data — every finding is generated by code from the parameterization in Table 1 — so nothing answers to the description “data that support the findings”; the artifact that determines reproducibility is the code, together with the complete numerical settings, and the statement is silent on both. Second, even taken at face value, availability upon request from the corresponding author does not meet contemporary community standards for computational work: it makes reproducibility contingent on the authors’ discretion and continued reachability, cannot be verified at review, and is the practice that data-availability policies of this journal’s type were written to replace — repository or supplementary-file deposit is the norm, with author-mediated access reserved for legally or ethically restricted material, of which there is none here. Community standards for computational finance therefore require: (a) deposit of the pricing and Monte Carlo code (the paper states seeds are fixed, which is only meaningful if the code is available); (b) a complete settings table per numerical exhibit — N, T, X₀, ψ-quadrature and grid, truncation U, lower cutoff ε, solver, rtol, atol — none of which is currently complete (Criterion 3); and (c) a computational-cost report: the semi-analytical route requires two complex Riccati systems plus the chain ODE on a ψ-grid per monitoring date, and a timing comparison against Monte Carlo at matched accuracy is the relevant evidence that the method adds value at, say, N = 252. Item (b) is not optional: at present the headline tables cannot be reproduced from the manuscript alone, since T and X₀ must be reverse-engineered from figure intercepts.

Revision requirements:

Major comments:

1. Delineate and reconcile with [20]: inheritance paragraph, disclosure to the editor, and quantitative reproduction of published numbers at χ = 0 of [20]. (Criteria 1, 2, 6)

2. Recompute headline tables at rtol ≤ 10^-9^ or round to demonstrated accuracy; explain the non-monotone rtol errors; disclose full settings per table; reconcile §3.2’s truncation guidance with Table 4 via an adaptive rule; add T and X₀ to Table 1. (Criteria 3, 4, 7)

3. Replace Assumptions 3 and 4 with checkable sufficient conditions at the arguments used (0, −j, −2j, the strip), citing Andersen–Piterbarg and Glasserman–Kim; add a verification argument to Proposition 2 and the conditioning step for the chain; cite a precise comparison theorem in Lemma 1. (Criterion 3)

4. Relabel K(0) = 1 and K(−j) = eʳᵀ as code sanity checks; remove “martingale admissibility” claims tied to them. (Criteria 4, 6)

5. Position the model within the linear–quadratic transform literature; justify the signed-Gaussian specification against the affine CIR-intensity alternative; state the cost of the zero W²–W⁴ correlation; address the scale indeterminacy (λ, α̃, β, λ₀) → c(·), σ → σ/c, χ → χ/c² by imposing an explicit normalization. (Criteria 1, 3)

6. Report the channel-additivity finding; quantify the ρ_1_ effect; remove or ground the risk-premium language; qualify all economic conclusions as conditional on an uncalibrated parameterization. (Criterion 4)

7. Replace the “available upon reasonable request” Data Availability Statement with a code-availability statement and public repository deposit (code with fixed seeds and per-table settings); add a timing comparison versus Monte Carlo. (Criterion 7)

Minor comments:

1. Cut redundancy, fix the dash-encoding defect globally, correct the minor items under Criterion 5, and adopt a colorblind-safe palette. (Criterion 5)

Recommendation

Major revision. Under a soundness-based rubric, the manuscript’s core is salvageable: the derivations are correct — I verified them independently — and the numerical study, once its settings are disclosed and its precision honestly stated, is a legitimate validation of a legitimate (if incremental) model. The failures are concentrated in verifiability rather than in the mathematics: undisclosed numerical settings, precision beyond demonstrated accuracy, assumptions that assume the theorems’ hard parts, a mislabeled tautological check, an unclarified relationship to a near-identical predecessor paper, and no code deposit. Every one of these is fixable, and criteria 2, 3, 4 and 7 should be re-examined strictly on resubmission.

References:

Ahn, D.-H., Dittmar, R. F., & Gallant, A. R. (2002). Quadratic term structure models: Theory and evidence. The Review of Financial Studies, 15(1), 243–288. https://doi.org/10.1093/rfs/15.1.243

Elliott, R. J., & Lian, G.-H. (2013). Pricing variance and volatility swaps in a stochastic volatility model with regime switching: Discrete observations case. Quantitative Finance, 13(5), 687–698. https://doi.org/10.1080/14697688.2012.676208

Leif B. G. Andersen and Vladimir V. Piterbarg (2007). Moment Explosions in Stochastic Volatility Models. Finance and Stochastics, Vol. 11, pp. 29–50 (2007). https://doi.org/10.1007/s00780-006-0011-7

Broadie, Mark and Jain, Ashish (2008). The Effect of Jumps and Discrete Sampling on Volatility and Variance Swaps. International Journal of Theoretical and Applied Finance, 11(8), 761–797. https://doi.org/10.1142/S0219024908005032

Cheng, P., & Scaillet, O. (2007). Linear quadratic jump diffusion modeling. Mathematical Finance, 17(4), 575–598. https://doi.org/10.1111/j.1467-9965.2007.00316.x

Glasserman, P., & Kim, K.-K. (2010). Moment explosions and stationary distributions in affine diffusion models. Mathematical Finance, 20(1), 1–33. https://doi.org/10.1111/j.1467-9965.2009.00387.x

X.-J. He, W. Chen, T. Lu, S. Lin (2025). Variance and volatility swap valuation with stochastic

liquidity and regime switching stochastic volatility. Communications in Nonlinear Science and Numerical Simulation 154 (2025) 109558.

W. Zheng, Y. K. Kwok (2014). Closed form pricing formulas for discretely sampled generalized

variance swaps, Mathematical Finance 24, 855–881. https://doi.org/10.1111/mafi.12016

Leippold, M., & Wu, L. (2002). Asset pricing under the quadratic class. Journal of Financial and Quantitative Analysis, 37(2), 271–295. https://doi.org/10.2307/3595006

Song-Ping Zhu and Guang-Hua Lian (2015). Analytically Pricing Volatility Swaps under Stochastic Volatility. Journal of Computational and Applied Mathematics, 288, 332–340. https://doi.org/10.1016/j.cam.2015.04.036

W. Zheng, Y. K. Kwok (2014). Closed form pricing formulas for discretely sampled generalized

variance swaps, Mathematical Finance 24, 855–881. https://doi.org/10.1111/mafi.12016

Reviewer #2: Reviewer Report on "Variance and Volatility Swap Valuation with Nonlinear Liquidity--Feedback Stochastic Volatility and Regime Switching" (PONE-D-26-35832) by Jing Fu and Ke Wang

Please see the attached report.

**********

what does this mean?). If published, this will include your full peer review and any attached files.

If you choose “no”, your identity will remain anonymous but your review may still be made public.

**Do you want your identity to be public for this peer review?**  For information about this choice, including consent withdrawal, please see our Privacy Policy

Reviewer #1: No

Reviewer #2: No

**********

[NOTE: If reviewer comments were submitted as an attachment file, they will be attached to this email and accessible via the submission site. Please log into your account, locate the manuscript record, and check for the action link "View Attachments". If this link does not appear, there are no attachment files.]

To ensure your figures meet our technical requirements, please review our figure guidelines: https://journals.plos.org/plosone/s/figures

You may also use PLOS’s free figure tool, NAAS, to help you prepare publication quality figures: https://journals.plos.org/plosone/s/figures#loc-tools-for-figure-preparation.

NAAS will assess whether your figures meet our technical requirements by comparing each figure against our figure specifications.

Attachment Submitted filename: referee_report_criteria_based-2 v5.pdf

Attachment Submitted filename: Reviewer_report_PONE-D-26-35832.pdf

# Author comment: Author response to Decision Letter 1

**DOI:** 10.1371/journal.pone.0355147.r002

4 Oct 2026

\documentclass[11pt,a4paper]{article}

Usepackage[margin=1in,footskip=30pt]{geometry}

Usepackage[T1]{fontenc}Usepackage{lmodern}

Usepackage{microtype,amsmath,amssymb,xcolor,xurl,needspace}

Usepackage[hidelinks]{hyperref}

\definecolor{responseheading}{RGB}{29,66,97}

\definecolor{reviewgray}{gray}{0.30}

\setlength{\parindent}{0pt}\setlength{\parskip}{6pt}

\setlength{\emergencystretch}{2em}\pagestyle{plain}

\newcommand{\reviewersection}[1]{\par\noindent{\Large\bfseries\color{responseheading}#1}\par\medskip}

\newcommand{\reviewcomment}[2]{\par\Needspace{8\baselineskip}\noindent\textbf{#1}\par

\begin{quote}\small\color{reviewgray}#2\end{quote}}

\newcommand{\groupheading}[1]{\par\medskip\Needspace{10\baselineskip}\noindent\textbf{#1}\par}

\begin{document}

\reviewersection{Response to Reviewers}

Dear Editor and Reviewers,

Thank you for the time and effort you have devoted to reviewing our manuscript, “Variance and volatility swap valuation with nonlinear liquidity-feedback stochastic volatility and regime switching” (PONE-D-26-35832). These comments are all valuable and very helpful for revising and improving our paper, and they are also of important guiding significance to our research. We have carefully studied the comments and made revisions, and we explain each revision below with reference to the revised clean manuscript. We hope that these revisions will meet with your approval.

Both the clean and marked manuscripts use the provided PLOS ONE template. In the marked copy, additions and revised passages are blue, unchanged text is black, and deleted or superseded material is omitted. All locations below refer to the revised clean English manuscript. Reference [20] in the original submission is He et al., numbered [23] in the revision.

\groupheading{Editorial Formatting Requirement}

\reviewcomment{Editorial Comment E-01}{Use the official PLOS ONE LaTeX template for all revised manuscripts.}

\textbf{Response}\enspace Thank you for specifying the required format. We have reformatted the clean and marked manuscripts using the provided PLOS ONE LaTeX template, version 3.8, and its plos2025 bibliography style. The title page, margins, headings, line numbering, references, and page footer follow the template. Figure captions remain in the manuscripts, and the 13 figures have been prepared as separate TIFF files. The marked manuscript shows additions and revised text in blue and omits deleted or superseded material.

\groupheading{Editorial Requirement — Code Sharing}

\reviewcomment{Editorial Comment E-02}{Author-generated code underlying the numerical results must be deposited in a public repository, and a Data Availability Statement must be provided.}

\textbf{Response}\enspace We have deposited all author-generated code underlying the numerical results in a public Zenodo repository. The archived reproducibility package is publicly accessible at https://doi.org/10.5281/zenodo.23044037 and is licensed under the MIT License. It contains the complete source code, fixed random seeds, numerical settings, generated numerical outputs underlying the reported tables and figures, and detailed reproduction instructions. Access does not require author-mediated permission. The manuscript and the Data Availability Statement in the submission system have been updated accordingly.

\clearpage\reviewersection{Response to Reviewer 1 (Report A)}

Dear Reviewer,

Thank you for your careful reading and constructive comments. We address each point below.

The compound requests in Report A are separated into the existing response identifiers. The quotations below are relevant original excerpts from the detailed criteria or the concluding requests; source pages are given for each. Only line wrapping and typographic notation have been normalized.

\reviewcomment{Reviewer comment A-M1(a)}{(a) A dedicated paragraph stating precisely what is inherited from [20] (model backbone, transform architecture, numerical scheme) and what is new (the feedback term, the associated Riccati forcings, the benchmark decomposition).}

{\small\textit{Source: Report A, p. 2.}}

\textbf{Response}\enspace Thank you for asking us to make the contribution more precise. We have added a dedicated paragraph identifying the no-feedback dynamics, regime-dependent variance target, signed OU factor, simple-return contract definitions and two-stage transform architecture inherited from He et al. The extension is the quadratic term $\chi\lambda^2$ in the variance target, which adds the forcings $\epsilon\chi\xi_2$ and $\epsilon\chi\xi_6$ and enables the nested analysis of direct and delayed liquidity effects. The numerical implementation follows the same transform architecture, with the feedback terms included and the integration stages treated explicitly. We have narrowed the novelty statement accordingly. We also clarify that setting $\chi=0$ recovers the stochastic-liquidity backbone; recovering the regime-switching Heston return model requires removing the direct liquidity channel as well.

\textbf{Location:} Introduction, p. 3, lines 62--72; Proposition 2, p. 6.

\reviewcomment{Reviewer comment A-M1(b)}{(c) If any text, figures, or derivations are shared with [20] or with other work by the authors, this must be disclosed to the editor. As it stands, a reader cannot determine from the manuscript alone whether the results at \ensuremath{\chi} = 0 --- which anchor every decomposition in Section 4 --- are new computations or republication.}

{\small\textit{Source: Report A, pp. 2, 3.}}

\textbf{Response}\enspace We thank you for emphasizing the need for clear disclosure. We have clarified the relationship with He et al. in both the Introduction and our letter to the editor. We confirm that no text, figures, tables, or previously published results from that article are presented as new results in this submission. The graph readings in Table 6 are explicitly attributed comparison data, while the prices in the ``Recomputed'' column were generated by our current implementation. We also confirm that the derivation materials were neither copied nor adapted from that article, and that its code was neither inherited nor modified. At the same time, we explicitly acknowledge the shared mathematical framework, including the exponential-quadratic ansatz, the no-feedback coefficient equations, and the two-stage conditioning construction. This clearly distinguishes the methodological basis of the study from the provenance of its materials and computations.

\textbf{Location:} Introduction, p.~3, lines 62--72; Table 6, p.~17; the editor letter in this response.

\reviewcomment{Reviewer comment A-M1(c)}{(b) A quantitative reconciliation: the nested \ensuremath{\chi} = 0 specification should reproduce the numbers published in [20], which would simultaneously provide the external code validation the paper currently lacks.}

{\small\textit{Source: Report A, p. 2.}}

\textbf{Response}\enspace We thank you for requesting an external numerical comparison. We set $\chi=0$, retained the direct liquidity channel, and used the example parameters on p.~11 of He et al.\ to compare our variance-swap prices with coordinates extracted from Fig.~1(a). Table~6 reports both the agreement and the remaining differences. We note that the referenced study presents its comparison between the model and Monte Carlo simulations only in graphical form and does not provide a numerical table. We can therefore only approximate its reported results from the figure. For example, at $N=1$, the plotted value is approximately 1427.0, whereas our price is 1425.1156, a difference of about $-1.86$ (0.131\%); at larger monitoring frequencies, some differences are about 0.4. We checked the payoff specification defined by the squared simple return, the percentage quotation convention, the maturity, and the parameters shown in the figure, without fitting any parameters to the graph. The reading tolerance of the graph is approximately 0.5 units, and the differences are computed from unrounded axis-converted coordinates. Accordingly, the revised text presents the comparison for the variance-swap leg as a graphical comparison, retains the unresolved differences, and does not present it as an exact reproduction.

\textbf{Location:} External comparison, p.~17, lines 365--378; Table~6, p.~17.

\reviewcomment{Reviewer comment A-M2(a)}{(a) The headline variance strike in Tables 2 and 6 (N = 12: 1620.0538) matches the rtol = 10\ensuremath{{}^{-8}} row of Table 5 to every printed digit (1620.053829), and Table 5 itself shows this tolerance is off by 5.5\ensuremath{\times}10\ensuremath{{}^{-2}} from the rtol = 10\ensuremath{{}^{-10}} benchmark (1619.998354): the headline numbers were produced at a tolerance the paper's own diagnostics deem insufficient, and are printed to four decimals anyway, while \S{}4.6 recommends the tighter tolerance ``for final production runs'' without applying it.}

{\small\textit{Source: Report A, p. 3.}}

\textbf{Response}\enspace Thank you for identifying the inconsistency between the reported precision and the convergence results. We have recomputed all retained price exhibits with DOP853 using relative and absolute tolerances of $10^{-10}$ and $10^{-12}$, respectively, and updated the dependent differences and contribution ratios. At $N=12$, the variance strike is now 1619.9988; at $N=252$, it is 1606.2044, replacing the original 1606.5109. Tighter-tolerance and Fourier-refinement checks support the four-decimal presentation of the deterministic production prices. Monte Carlo estimates are reported separately with confidence intervals based on independent antithetic pair means. This makes the distinction between displayed precision and simulation uncertainty explicit.

\textbf{Location:} Table 2, p. 11; Table 3, p. 11; Table 9, p. 24.

\reviewcomment{Reviewer comment A-M2(b)}{(b) Table 5's error is non-monotone in rtol (5.447\ensuremath{\times}10\ensuremath{{}^{-2}} at 10\ensuremath{{}^{-7}} versus 5.547\ensuremath{\times}10\ensuremath{{}^{-2}} at 10\ensuremath{{}^{-8}}); unexplained, this undermines the convergence claim.}

{\small\textit{Source: Report A, p. 3.}}

\textbf{Response}\enspace We thank the reviewer for pointing out the non-monotone behavior of the error with respect to rtol. We traced the main cause to the original chain integration, which crossed the source junction without terminating the integration at that point, so that the adaptive solver could miss part of a short active interval. With the model and nominal tolerances held fixed, splitting the integration changes the values at $N=12$ for relative tolerances $10^{-7}$ and $10^{-8}$ from 1619.943884 and 1620.053829 to 1619.998789 and 1619.998779, respectively. The revised implementation treats the two stages explicitly and has been verified against the corrected historical equations and a second ODE solver. We also explain that a local solver tolerance does not guarantee monotone error in the final strike, since the strike is formed from combinations of transform values that are susceptible to cancellation. The revised diagnostics and the archived historical comparison records document both the source of the discrepancy and the corresponding correction.

\textbf{Location:} Numerical implementation, p.~10; junction diagnosis, pp.~16--17; Appendix~B, pp.~22--25, including Table~9.

\reviewcomment{Reviewer comment A-M2(c)}{(c) The diagnostics were run at different, undisclosed monitoring frequencies: Converged volatility strike (39.731069) of Table 4 matches N = 52 row of Table 2, while the reference (39.775055) of Table 5 matches N = 12; neither table states N or T.\par\medskip (e) The maturity T is never stated (evidently 1, inferred from the frequency axis) and the initial regime X\ensuremath{{}_{0}} is never stated (evidently e\ensuremath{{}_{1}}, inferred from Fig. 14's intercepts 0.08 and 0.08 + \ensuremath{\chi}\ensuremath{\lambda}\ensuremath{{}_{0}}\ensuremath{{}^{2}} = 0.12); both belong in Table 1, along with a unified generator notation (v\ensuremath{{}_{12}}/v\ensuremath{{}_{21}} in the table versus g\ensuremath{{}_{ij}} in eq. (1)). Every table should disclose its full settings: N, T, quadrature rule and \ensuremath{\psi}-grid, lower cutoff \ensuremath{\epsilon} and its bias, solver, rtol, atol.}

{\small\textit{Source: Report A, pp. 3, 4.}}

\textbf{Response}\enspace Thank you for specifying the information required to reproduce the numerical results. Table~1 now states $T=1$ year, $X_0=e_1$, the baseline $N=12$, the quotation factor, and the fixed liquidity scale. Table~7 maps each numerical exhibit to its corresponding parameter sweep and method, while the saved records contain the Fourier domain employed, the number of nodes, the solver tolerances, the random seeds, and the actual Monte Carlo step counts. The generator entries are denoted consistently by $g_{12}$ and $g_{21}$. The Fourier diagnostic is explicitly labeled $N=52$ and the ODE diagnostic $N=12$, which explains the difference in their reference strikes. In addition, we distinguish 147 production rows, 81 distinct model-parameter sets, and 115 pricing cases; the seven REF20 production frequencies and the 15 separate graphical comparison points are listed separately, with Table~6 presenting six selected comparison points. These clarifications make the numerical settings and the coverage counts traceable.

\textbf{Location:} Table~1, p.~10; Table~7, pp.~22--23; Table~2, p.~11.

\reviewcomment{Reviewer comment A-M2(d)}{(d) The claim in \S{}3.2 that ``U = 50 -- 100 is typically adequate'' is contradicted by Table 4 (U = 40 off by 1.2335, \ensuremath{\approx}3\% of the strike; U = 120 needed for 10\ensuremath{{}^{-5}}); a fixed U is not meaningful in any case, since the required truncation grows with monitoring frequency roughly like \ensuremath{\sqrt{\vphantom{x}}}(N/T) --- an adaptive rule tied to the per-interval variance should replace it.}

{\small\textit{Source: Report A, pp. 3, 4.}}

\textbf{Response}\enspace We thank the reviewer for pointing out the inconsistency in the truncation guidance. We have replaced the fixed range with the following adaptive rule: the initial value is taken as $U_0=\max\{40,8\sqrt{N/(Tv_0)}\}$, where $v_0=\eta_0+\sigma^2\lambda_0^2$. The algorithm compares node refinement on the current domain with that on an enlarged domain, and accepts the calculation as converged when both price differences fall below $2\times10^{-6}$. The final domain and node count are recorded for each production price. Integration starts at zero using interior Gauss--Legendre nodes; the manuscript states that the removable endpoint limit concerns the real part of the integrand. Appendix~B reports separate studies of the domain, node count, and lower cutoff. Since this acceptance criterion does not itself provide a rigorous tail-error bound, we describe it as an empirical convergence check.

\textbf{Location:} Volatility swap and numerical implementation, pp.~9--10; Table~10 and Fig.~13, pp.~24--25.

\reviewcomment{Reviewer comment A-M3(a)}{Assumption 3 is the Novikov condition, so Proposition 1 is immediate and the substantive question --- primitive parameter restrictions guaranteeing (8) --- is left open.}

{\small\textit{Source: Report A, p. 3.}}

\textbf{Response}\enspace We thank the reviewer for emphasizing the need to connect the martingale assumption to primitive parameters. Appendix~A now constructs a regime-independent exponential supersolution for the integral of $\eta+\sigma^2\lambda^2$. Two scalar Riccati explosion-time inequalities provide explicit sufficient conditions for Novikov's condition on the chosen finite horizon. The bound incorporates the feedback term and includes a Young-inequality allowance for the nonzero OU mean. For context, we also state the isolated all-horizon thresholds: $q<\tilde\gamma^2/(2\beta^2)$ for the OU quadratic integral and $q<\epsilon^2/(2\omega^2)$ for the CIR integral, while applying the coupled finite-horizon bound to the present model. Assumption~3 now refers to these parameter-based conditions, and the discussion cites Andersen and Piterbarg and Glasserman and Kim for the moment-explosion framework.

\textbf{Location:} Assumption~3, p.~5; Novikov bound, p.~20; Eq.~A.3, p.~20.

\reviewcomment{Reviewer comment A-M3(b)}{Assumption 4 assumes finiteness of the transform at \ensuremath{\psi} = -2j and on the shifted strip, exactly the moment-explosion territory mapped by Andersen--Piterbarg (2007) and Glasserman--Kim (2010). Since these functionals are finite iff Riccati systems of the very type the paper solves do not explode, checkable sufficient conditions are within reach and should be supplied. For example: at \ensuremath{\psi} = -2j the \ensuremath{\eta}-block is autonomous, \ensuremath{\xi}\ensuremath{{}_{2}}\ensuremath{{}^{\prime}} = \ensuremath{\frac12}\ensuremath{\omega}\ensuremath{{}^{2}}\ensuremath{\xi}\ensuremath{{}_{2}}\ensuremath{{}^{2}} + (2\ensuremath{\rho}\ensuremath{{}_{1}}\ensuremath{\omega} - \ensuremath{\kappa})\ensuremath{\xi}\ensuremath{{}_{2}} + 1 with \ensuremath{\xi}\ensuremath{{}_{2}}(0) = 0; if (\ensuremath{\kappa} - 2\ensuremath{\rho}\ensuremath{{}_{1}}\ensuremath{\omega})\ensuremath{{}^{2}} > 2\ensuremath{\omega}\ensuremath{{}^{2}} (baseline: 1.30 > 0.04) the quadratic has two positive real roots and \ensuremath{\xi}\ensuremath{{}_{2}} rises monotonically to the smaller one, hence is bounded for every horizon; a comparison argument then bounds the \ensuremath{\lambda}-block given the \ensuremath{\xi}\ensuremath{{}_{2}} bound. Closed-form thresholds for the building blocks (Cameron--Martin: exponential moments of \ensuremath{\int}\ensuremath{\lambda}\ensuremath{{}^{2}} up to \ensuremath{\tilde{\gamma}}\ensuremath{{}^{2}}/(2\ensuremath{\beta}\ensuremath{{}^{2}}); CIR: \ensuremath{\int}\ensuremath{\eta} up to \ensuremath{\kappa}\ensuremath{{}^{2}}/(2\ensuremath{\omega}\ensuremath{{}^{2}})) belong in the paper.}

{\small\textit{Source: Report A, p. 3.}}

\textbf{Response}\enspace We thank the reviewer for asking us to provide sufficient conditions that cover all transform arguments used in pricing. Appendix~A now gives scalar sufficient bounds for real exponential loadings on the interval $[-h,2+h]$, with $h=0.1$ in the tests reported here. These bounds cover the second moment, the shifted Fourier arguments at every real frequency, and the pre-monitoring stage with zero return loading and inherited terminal coefficients. We also state the two conditions required for the positive-root second-moment comparison: $\epsilon-2\rho_1\omega>0$ and $(\epsilon-2\rho_1\omega)^2>2\omega^2$. At the baseline parameters, these read $1.14>0$ and $1.2996>0.0392$. All 147 production rows pass the Novikov and strip tests, including the 20 nested-model rows and seven REF20 rows. These rows correspond to 81 model-parameter sets and 115 pricing cases; the separate graphical comparisons use an already verified no-feedback parameter set. We note that the above conditions are sufficient, so failure to satisfy them alone would not establish moment explosion. Assumption~4 and Table~8 now make this scope explicit.

\textbf{Location:} Assumption~4, p.~6; strip bound covering both stages, p.~21; Eq.~A.4, p.~21; Table~8, p.~23; coverage definitions, p.~23, lines 526--536.

\reviewcomment{Reviewer comment A-M3(c)}{Separately, Proposition 2 is established by coefficient matching only; a verification argument is needed (candidate solves the backward equation; local-martingale-plus-integrability identifies it with the conditional expectation under Assumption 4), along with an explicit proof of the conditioning-on-the-chain-path step behind the factorization into M(u\ensuremath{{}_{1}}).}

{\small\textit{Source: Report A, p. 3.}}

\textbf{Response}\enspace We thank the reviewer for identifying the missing verification step. We have supplemented coefficient matching with a path-conditioned martingale argument. For real loadings, the chained candidate is represented as a stochastic exponential. After stopping at the level $V=1+\eta+\lambda^2$, the tilted drifts yield a uniform Lyapunov bound and an exit-probability estimate proportional to $V(0)e^{CT}/n$. This probability tends to zero as the stopping level increases, thereby establishing that the density loses no mass and that the candidate equals the required conditional expectation. Analytic continuation then identifies the complex transform on the admissible strip. We separately explain why conditioning on the independent chain path leaves the regime dependence only in the additive scalar coefficient, and why averaging this factor yields the finite-state backward equation. The proof exploits the independence of the chain and the Brownian vector while retaining the dependence of the variance process on the chain. The Fourier representation is also justified through a damped limit.

\textbf{Location:} Verification argument, pp.~21--22, lines 485--508; Proposition~2, p.~6.

\reviewcomment{Reviewer comment A-M3(d)}{Lemma 1 should cite a comparison theorem valid for random drifts (Ikeda--Watanabe; Karatzas--Shreve Prop. 5.2.18).}

{\small\textit{Source: Report A, p. 3.}}

\textbf{Response}\enspace We thank the reviewer for requesting that we cite a precise comparison theorem. The proof of Lemma~1 now cites Theorem~1.1 and the localized form given in Remark~1.1 of Ikeda and Watanabe (1977). We explain how the square-root diffusion modulus, the adapted lower drift bound, and pathwise uniqueness of the comparison CIR process satisfy the conditions required by this theorem. Localization handles the random variance target, and a finite-horizon non-explosion argument permits removal of the stopping times. The Feller condition for the lower comparison process then yields the stated strict positivity. This provides a rigorous basis for the comparison step that was previously described only informally.

\textbf{Location:} Lemma~1, p.~5; Eq.~7, p.~5.

\reviewcomment{Reviewer comment A-M4}{Relabel K(0) = 1 and K(-j) = e\ensuremath{{}^{rT}} as code sanity checks; remove ``martingale admissibility'' claims tied to them. (Criteria 4, 6)}

{\small\textit{Source: Report A, p. 6.}}

\textbf{Response}\enspace Thank you for explaining why these identities do not establish martingale admissibility. We have relabelled $K_\chi(0)=1$ and $K_\chi(-\mathrm{j})=e^{rT/N}$ as code sanity checks. At these loadings the Riccati source terms vanish and the generator annihilates the all-ones vector, so the identities follow algebraically. The manuscript now uses the separate parameter-based Novikov and moment bounds to establish probabilistic admissibility. We have corrected the interpretation in both the transform discussion and the numerical diagnostics so that the evidence attached to each check is clear.

\textbf{Location:} Identity interpretation, p. 8, lines 225--229; numerical code checks, p. 23, lines 537--543.

\reviewcomment{Reviewer comment A-M5(a)}{Position the model within the linear--quadratic transform literature;}

{\small\textit{Source: Report A, p. 6.}}

\textbf{Response}\enspace Thank you for identifying the relevant transform literature. We have added Ahn, Dittmar and Gallant; Leippold and Wu; and Cheng and Scaillet, and now explicitly place the model within the established quadratic and linear-quadratic class. The term nonlinear refers to the dependence on signed liquidity stress in the original variables; augmenting the state with $\lambda^2$ gives the exponential-quadratic closure used here. We have therefore focused the contribution on the particular feedback specification, its induced Riccati forcings and the associated liquidity-channel analysis. This places the derivation and the scope of the novelty claim in their appropriate context.

\textbf{Location:} Introduction, p. 3, lines 62--72; Transform and monitoring attribution, p. 3, lines 73--77.

\reviewcomment{Reviewer comment A-M5(b)}{Originality also carries an unexercised opportunity cost: a fully affine alternative (Cox-Ingersoll-Ross (CIR) liquidity intensity \ensuremath{\ell}, loading \ensuremath{\sigma}\ensuremath{\sqrt{\vphantom{x}}}\ensuremath{\ell}, drift term \ensuremath{\chi}\ensuremath{\ell}) would deliver both channels with a nonnegative intensity and no quadratic extension; the paper should explain what the signed-Gaussian specification buys, especially since its own Fig. 9 shows the distinctive \ensuremath{\rho}\ensuremath{{}_{2}} channel is nearly flat.}

{\small\textit{Source: Report A, p. 2.}}

\textbf{Response}\enspace We thank the reviewer for this helpful comparison. We have clarified that the signed OU factor captures positive and negative liquidity-stress deviations, whose squared magnitude shifts the variance target; its Gaussian transition structure supports the exponential-quadratic representation adopted here. We also discuss a nonnegative CIR intensity as a parsimonious affine alternative, with return loading $\sigma\sqrt{\ell}$ and linear feedback term $\chi\ell$. The revised manuscript also notes that, under the baseline, the sensitivity to the direct-liquidity correlation is weak. The present analysis establishes the tractability of the selected specification; assessing whether it is empirically preferable would require estimation and comparison with the alternative model.

\textbf{Location:} Scale convention and model restrictions, p.~4.

\reviewcomment{Reviewer comment A-M5(c)}{state the cost of the zero W\ensuremath{{}^{2}}--W\ensuremath{{}^{4}} correlation;}

{\small\textit{Source: Report A, p. 6.}}

\textbf{Response}\enspace We thank the reviewer for asking us to make this restriction explicit. We now explain that a nonzero correlation between $W^2$ and $W^4$ introduces a mixed derivative term proportional to $\sqrt\eta\,f_{\eta\lambda}$. Under the present specification, this term generates additional terms beyond the retained linear-quadratic basis. The zero-correlation assumption preserves the aforementioned closure property but restricts the dependence structure that the model can represent. We have added this explanation to the model assumptions and characterized the restriction as a tractability trade-off whose empirical suitability remains to be assessed.

\textbf{Location:} Scale convention and model restrictions, p.~4.

\reviewcomment{Reviewer comment A-M5(d)}{address the scale indeterminacy (\ensuremath{\lambda}, \ensuremath{\tilde{\alpha}}, \ensuremath{\beta}, \ensuremath{\lambda}\ensuremath{{}_{0}}) \ensuremath{\to} c(·), \ensuremath{\sigma} \ensuremath{\to} \ensuremath{\sigma}/c, \ensuremath{\chi} \ensuremath{\to} \ensuremath{\chi}/c\ensuremath{{}^{2}} by imposing an explicit normalization. (Criteria 1, 3)}

{\small\textit{Source: Report A, p. 6.}}

\textbf{Response}\enspace We thank the reviewer for pointing out the scale indeterminacy. We now give the complete transformation: multiplying $(\lambda,\tilde\alpha,\beta,\lambda_0)$ by $c>0$, while dividing $\sigma$ by $c$ and $\chi$ by $c^2$, leaves the return dynamics and the feedback term unchanged, and $\tilde\gamma$ also remains unchanged. For the positive-initial-stress parameter family studied here, we fix $\lambda_0=0.20$ as the liquidity unit. This removes one scale degree of freedom and is well defined for both $\chi=0$ and $\sigma=0$. The manuscript also states that zero or negative initial stress requires a separately specified convention. As an implementation-level check, rescaling by $c=5$ changes the tested variance and absolute-return prices by at most $3.91\times10^{-9}$ and $1.42\times10^{-14}$, respectively.

\textbf{Location:} Scale convention and model restrictions, p.~4; Table~1, p.~10; Appendix~B. Numerical settings, convergence and reproducibility, p.~22; numerical rescaling test, p.~23, lines 540--543.

\reviewcomment{Reviewer comment A-M6(a)}{One clean supported finding is present in the numbers but never stated and should be: the two channels are almost exactly additive at baseline (cross-channel interaction +0.0866 out of 363.48 for the variance swap; -0.0921 out of 4.66 for the volatility swap), so the interesting nonlinearities are within the feedback channel, not across channels.}

{\small\textit{Source: Report A, p. 4.}}

\textbf{Response}\enspace We thank the reviewer for drawing attention to the near-additivity evident in the results. We now report the interaction term explicitly as $P(\chi,\sigma)-P(0,\sigma)-P(\chi,0)+P(0,0)$. At the baseline $N=12$, this interaction is approximately $+0.0866$ variance units and $-0.0921$ absolute-return units, corresponding to $0.024\%$ and $-1.976\%$ of the respective total two-channel increments. Accordingly, the revised discussion describes the two channels as approximately additive at this baseline and locates the main nonlinear effects within the feedback channel. This conclusion is confined to the reported parameterization and is not extended to all admissible settings.

\textbf{Location:} Table~4, p.~16; Fig.~9, p.~16.

\reviewcomment{Reviewer comment A-M6(b)}{(c) \S{}4.5 characterizes the \ensuremath{\rho}\ensuremath{{}_{1}} channel as ``significant'' when the full sweep \ensuremath{\rho}\ensuremath{{}_{1}} \ensuremath{\in} [-0.75, 0.75] moves the strike by roughly 1.5\%, against a 25\% feedback contribution at \ensuremath{\chi} = 1; magnitudes should be quantified, not characterized.}

{\small\textit{Source: Report A, p. 4.}}

\textbf{Response}\enspace Thank you for asking us to quantify this effect. We have replaced the description ``significant'' with the observed price changes. As $\rho_1$ varies from $-0.75$ to $0.75$, the variance strike increases from 1615.5112 to 1642.6992, a change of 1.683\%, while the absolute-return strike changes by about 0.098\%. We present these as model sensitivities under the specified parameters, without a statistical-significance interpretation. The separate $\rho_2$ sweep is also identified clearly and includes negative, zero and positive values.

\textbf{Location:} Fig 8, p. 15.

\reviewcomment{Reviewer comment A-M6(c)}{(d) The ``risk premium'' language in \S{}4.3 is undefined in a Q-only model with no specified market prices of risk; either introduce the P-measure link or remove the interpretation.}

{\small\textit{Source: Report A, p. 4.}}

\textbf{Response}\enspace We thank the reviewer for identifying the unsupported risk-premium interpretation. The model is specified under the risk-neutral measure, and the present study does not estimate physical-measure dynamics or market prices of risk. We have therefore replaced that language with model-implied changes in risk-neutral swap strikes. The liquidity-mean discussion and the concluding interpretation now use this terminology consistently. A risk-premium interpretation would require a separately specified and estimated link between the physical and risk-neutral measures.

\textbf{Location:} Model-implied price contribution, p.~12, lines 325--327; conclusion limitations, p.~19, lines 410--415.

\reviewcomment{Reviewer comment A-M6(d)}{Additionally, all economic conclusions should be explicitly flagged as conditional on an uncalibrated parameterization --- the paper says the baseline is ``not intended to calibrate or represent any specific market data,'' and the abstract and conclusion should carry that qualification rather than bury it in \S{}4.1.}

{\small\textit{Source: Report A, p. 4.}}

\textbf{Response}\enspace We thank the reviewer for asking us to define the scope of the economic conclusions more consistently. We have revised the Abstract, the introduction to the numerical section, the mechanism discussion, and the Conclusion to make explicit that the results are conditional on illustrative, uncalibrated parameters. In particular, the relative importance of the two liquidity channels holds only for the reported scenarios. The revised limitations section further states that drawing market-wide conclusions about their magnitudes or ranking would require empirical estimation and validation. This qualification now accompanies the main conclusions and the parameter table.

\textbf{Location:} Abstract, p.~1; Calibration and nonlinear-payoff limitations, pp.~18--19; conclusion limitations, p.~19, lines 410--415.

\reviewcomment{Reviewer comment A-M7(a)}{Replace the ``available upon reasonable request'' Data Availability Statement with a code-availability statement and public repository deposit (code with fixed seeds and per-table settings); add a timing comparison versus Monte Carlo. (Criterion 7)}

{\small\textit{Source: Report A, p. 6.}}

\textbf{Response}\enspace We thank the reviewer for emphasizing the importance of unrestricted access to the computational materials. We have now deposited the complete reproducibility package in the Zenodo public repository, publicly accessible at https://doi.org/10.5281/zenodo.23044037. The archived package contains the pricing and Monte Carlo source code, fixed random seeds, complete numerical settings, parameter-admissibility certificates, source data underlying the tables and figures, computational-timing scripts, and detailed reproduction instructions. The archive is accessible without author-mediated permission. The manuscript and the Data Availability Statement have been updated accordingly. The requested computational-time comparison between the transform-based method and Monte Carlo is addressed separately in Response A-M7(c) and reported in Appendix B of the revised manuscript.

\reviewcomment{Reviewer comment A-M7(b)}{(a) deposit of the pricing and Monte Carlo code (the paper states seeds are fixed, which is only meaningful if the code is available); (b) a complete settings table per numerical exhibit --- N, T, X\ensuremath{{}_{0}}, \ensuremath{\psi}-quadrature and grid, truncation U, lower cutoff \ensuremath{\epsilon}, solver, rtol, atol --- none of which is currently complete (Criterion 3);}

{\small\textit{Source: Report A, pp. 5, 6.}}

\textbf{Response}\enspace We thank the reviewer for these comments.

(a) The complete reproducibility package has now been deposited publicly at https://doi.org/10.5281/zenodo.23044037. It contains the current production pricing and Monte Carlo code, complete parameter records, fixed random seeds, generated numerical outputs, confidence-interval calculations, source data for the figures, and scripts for rebuilding the numerical exhibits.

(b) The revised manuscript now reports the common production settings and identifies the settings associated with each numerical exhibit. The Zenodo archive provides the complete per-exhibit numerical records, including \(N\), \(T\), \(X_0\), the Fourier quadrature rule and accepted truncation domain \(U\), lower-endpoint treatment, solver, rtol, atol, random seeds, and Monte Carlo step counts. These materials allow the reported tables and figures to be independently reproduced.

\reviewcomment{Reviewer comment A-M7(c)}{(c) a computational-cost report: the semi-analytical route requires two complex Riccati systems plus the chain ODE on a \ensuremath{\psi}-grid per monitoring date, and a timing comparison against Monte Carlo at matched accuracy is the relevant evidence that the method adds value at, say, N = 252.}

{\small\textit{Source: Report A, p. 6.}}

\textbf{Response}\enspace We thank the reviewer for requesting a timing comparison at a relevant monitoring frequency. We have added an actual $N=252$ comparison on the reported hardware, specifying the timing boundaries and the thread settings of the numerical libraries. The median transform time for both swap legs is 0.1754 seconds. Monte Carlo with 300,000 paths takes 47.19 seconds at 1008 steps and 92.55 seconds at 2016 steps. The manuscript reports these timings together with the corresponding prices and paired confidence half-widths, using common empirical targets of 2 variance quotation units and 0.03 absolute-return quotation units. The finer-step result provides an additional check on discretization sensitivity. This supports a practical comparison at the stated tolerances; a rigorously matched total-error comparison would additionally require a certified discretization-bias bound, which is not established here.

\textbf{Location:} Computational cost, p.~26.

\groupheading{Minor comments and method attribution}

\reviewcomment{Reviewer comment A-Minor-01}{First, redundancy: the two-channel mechanism paragraph appears nearly verbatim twice in the Introduction, and the variance-versus-volatility sensitivity explanation is repeated in \S{}\S{}4.3, 4.4, 4.5, 4.6, 4.8 and the Conclusion;}

{\small\textit{Source: Report A, p. 4.}}

\textbf{Response}\enspace Thank you for pointing out the repetition. We have consolidated the repeated mechanism discussion in the Introduction and shortened the recurring sensitivity explanations in the numerical section. The revised presentation states the model inheritance and contribution together, then uses the numerical discussion to explain the specific effects shown in each exhibit. We have also aligned the interpretation with the absolute-return contract defined in the paper. These changes reduce repetition while preserving the reasoning needed to understand the results.

\textbf{Location:} Introduction, pp. 2--3; Numerical analysis, pp. 10--19.

\reviewcomment{Reviewer comment A-Minor-02}{Fig. 1 duplicates Figs. 2--3 exactly;}

{\small\textit{Source: Report A, p. 4.}}

\textbf{Response}\enspace Thank you for identifying the duplicated validation figure. We have removed the original combined figure and retained the two separate validation plots, now numbered Figs. 1 and 2. Both have been regenerated from the revised numerical results. Each plot presents one payoff leg clearly, and all distinct validation evidence is retained. The separate figure package follows the revised numbering and excludes the duplicated combined plot.

\textbf{Location:} Fig 1, p. 12; Fig 2, p. 12.

\reviewcomment{Reviewer comment A-Minor-03}{Tables 3--5 and Figs. 11--12 are diagnostics best moved to an appendix.}

{\small\textit{Source: Report A, pp. 4, 5.}}

\textbf{Response}\enspace We thank the reviewer for suggesting a clearer separation between the main results and the numerical diagnostics. We have moved the ODE, Fourier, and time-step studies to Appendix~B while retaining the details required for reproduction. Original Table~3 is now split into Tables~11 and~12, original Table~4 is now Table~10, and original Table~5 is now Table~9; original Figs.~11 and~12 are now Figs.~13 and~12, respectively. These diagnostics have also been recomputed following the numerical corrections. This arrangement keeps the main discussion more focused while making the supporting checks easier to locate.

\textbf{Location:} Appendix~B: Table~9, p.~24; Tables~10--12, pp.~24--25; Fig.~12, p.~24; Fig.~13, p.~25.

\reviewcomment{Reviewer comment A-Minor-04}{Second, a typographic defect: in the copy reviewed, en/em-dashes are dropped in many compounds (``returnliquidity'', ``liquiditystress'', ``pricevariance'', ``designnamely'' on pp. 15, 17, 19, 24) while the same compounds are correctly hyphenated elsewhere (p. 4) --- evidently a character-encoding problem in the source that must be fixed globally; check also the ``U = 50--100'' range punctuation in \S{}3.2 and the capitalization of ``Fourier'' in reference [5].}

{\small\textit{Source: Report A, p. 5.}}

\textbf{Response}\enspace We thank the reviewer for pointing out these typographic issues. We have replaced the problematic dash characters with stable TeX forms and adopted T1 encoding with Latin Modern fonts. We have checked the affected compounds and ranges in the rendered manuscript and corrected the capitalization of ``Fourier'' in the references. The former fixed truncation range has been replaced by the adaptive rule described in response A-M2(d), so that the numerical guidance and its typography are now consistent.

\textbf{Location:} Throughout the revised manuscript, including the Volatility swap discussion, pp.~9--10, and the References, pp.~26--27.

\reviewcomment{Reviewer comment A-Minor-05}{\S{}4.4 calls E[\ensuremath{\lambda}\ensuremath{{}^{2}}] = Var(\ensuremath{\lambda}) + (E[\ensuremath{\lambda}])\ensuremath{{}^{2}} a ``qualitative approximation'' when it is an exact identity with closed-form components for an OU process;}

{\small\textit{Source: Report A, p. 5.}}

\textbf{Response}\enspace Thank you for pointing out that this relation is exact. We have replaced the approximation wording and now give the finite-time OU second moment explicitly in Eq. (38): $\mathbb E[\lambda_t^2]=[\tilde\alpha+(\lambda_0-\tilde\alpha)e^{-\tilde\gamma t}]^2+\beta^2(1-e^{-2\tilde\gamma t})/(2\tilde\gamma)$. The first term is the squared conditional mean and the second is the conditional variance. Their sum is the exact quantity used in the target calculation. The surrounding explanation now distinguishes this identity from the qualitative interpretation of its competing components.

\textbf{Location:} Eq. 38, p. 13; Fig 11, p. 18.

\reviewcomment{Reviewer comment A-Minor-06}{m = 100 is a quoting convention, not an ``annualization scaling factor'';}

{\small\textit{Source: Report A, p. 5.}}

\textbf{Response}\enspace We thank the reviewer for noting the inaccurate description of $m$. We now clarify that $m=100$ converts decimal returns into quotation units expressed in percentage terms. Annualization is governed by the time normalization and the sampling convention adopted in the contract definitions. We have correspondingly revised the descriptions of both realized measures so that quotation units and time scaling are consistently distinguished.

\textbf{Location:} variance-swap definition (Eq.~30), p.~8; volatility swap, p.~9.

\reviewcomment{Reviewer comment A-Minor-07}{the superscript in K\ensuremath{{}^{\chi}} collides with the parameter \ensuremath{\chi};}

{\small\textit{Source: Report A, p. 5.}}

\textbf{Response}\enspace Thank you for noting the ambiguity in the transform notation. We now write the model-dependent transform as $K_\chi$ throughout the equations and the accompanying text. This makes the dependence on the feedback coefficient explicit and avoids the conflicting superscript. The payoff-strike notation is retained where its meaning is unambiguous.

\textbf{Location:} Proposition 2, p. 6.

\reviewcomment{Reviewer comment A-Minor-08}{Assumption 1's independence statement should say plainly that X is independent of (W\ensuremath{{}^{1}}, \ldots\{\}, W\ensuremath{{}^{4}});}

{\small\textit{Source: Report A, p. 5.}}

\textbf{Response}\enspace We thank the reviewer for requesting a precise independence statement. Assumption~1 now states explicitly that the process $X$ is independent of the Brownian vector $(W^1,W^2,W^3,W^4)$. We also clarify that the variance process depends on $X$ through its drift term. The same distinction is employed in the chain-conditioning argument, thereby ensuring that the probabilistic assumption and its role in the proof remain consistent.

\textbf{Location:} Assumption~1, p.~4; verification and conditioning on the chain, pp.~21--22.

\reviewcomment{Reviewer comment A-Minor-09}{the N = 1 case is not a traded contract design and should be labeled a stress case;}

{\small\textit{Source: Report A, p. 5.}}

\textbf{Response}\enspace We thank the reviewer for clarifying the interpretation of the $N=1$ case. We have now labeled it as a coarse-monitoring stress case in the numerical discussion and the relevant figure caption. This case is retained to examine sensitivity to the monitoring interval, and the text no longer presents it as a representative traded-contract design. The payoff definition itself remains unchanged.

\textbf{Location:} Formula validation, p.~11; caption of the feedback-effects figure (Fig.~3).

\reviewcomment{Reviewer comment A-Minor-10}{and several figures rely on red/green contrasts (Figs. 4, 6--8, 10) that are not colorblind-safe, with partially illegible legends in Figs. 5 and 13.}

{\small\textit{Source: Report A, p. 5.}}

\textbf{Response}\enspace We thank the reviewer for pointing out the accessibility and legibility issues in the figures. We have regenerated the affected figures using a colorblind-safe palette together with distinct line styles and markers, so that the curves remain distinguishable under grayscale printing. The legends have been enlarged, and the heatmap now uses a sequential palette with explicit parameter ticks. Original Figs.~5 and~13 correspond to revised Figs.~4 and~10, respectively. The 13 separate TIFF files are output at 600 dpi in RGB and labeled in 8--12 point Arial font; the mappings between their source data and the figures have been retained with the figure records.

\textbf{Location:} Fig.~4, p.~13; Fig.~10, p.~18.

\reviewcomment{Reviewer comment A-Minor-11}{Reference attribution also needs care: the sign-function representation of E|e\ensuremath{{}^{y}} - 1| and the two-stage forward-transform construction read as if introduced here and should be attributed to their sources (the Zheng and Kwok 2014 / Elliott and Lian 2013 lineage; Zhu and Lian 2015; Broadie and Jain 2008 for discrete monitoring).}

{\small\textit{Source: Report A, p. 5.}}

\textbf{Response}\enspace We thank the reviewer for pointing out the need for more explicit attribution. We now acknowledge the sources of the sign-function representation, the two-stage forward-transform construction, and the discrete-monitoring methods at the points where they are used. The revised discussion cites He et al., together with Elliott and Lian, Zheng and Kwok, Zhu and Lian, and Broadie and Jain, to provide the relevant methodological context. The contribution paragraph now clearly distinguishes these established tools from the feedback-specific extension developed in this manuscript.

\textbf{Location:} Introduction, p.~3, lines 62--72; transform and monitoring attribution, p.~3, lines 73--77; Fourier attribution, p.~10, lines 276--278.

\clearpage\reviewersection{Response to Reviewer 2 (Report B)}

Dear Reviewer,

Thank you for your careful reading and constructive comments. We address each point below.

The original numbered comments are reproduced below, with line wrapping and typographic notation normalized.

\reviewcomment{Reviewer comment B-01}{The abstract is too detailed and contains more information than necessary, including the sentence on p.1 ll.17-19. It should be revised to be more concise and focused.}

{\small\textit{Source: Report B, comment 1, p. 1.}}

\textbf{Response}\enspace We thank the reviewer for suggesting that the abstract be more focused. We have shortened it to state the pricing problem, the quadratic-feedback extension, the transform approach, and the principal numerical finding, while removing detailed constants and implementation-level statements. The revised abstract also identifies the absolute-return-based contract and clarifies that the channel comparison is conditional on an illustrative, uncalibrated parameterization. This allows readers to grasp the main contribution and its scope from the outset.

\textbf{Location:} Abstract, p.~1.

\reviewcomment{Reviewer comment B-02}{p.2 ll.-15--16: The literature review on variance and volatility swap valuation under stochastic volatility should be expanded to include recent studies -- in particular those on rough volatility models, which are compatible with Fourier-based pricing techniques presented; see in particular [1, 2, 3]. This addition will also provide a useful contrast with the proposed framework and highlight possible avenues for integration and future extensions.}

{\small\textit{Source: Report B, comment 2, p. 1.}}

\textbf{Response}\enspace Thank you for recommending these studies. We have added Horvath, Jacquier and Tankov; Salmon and SenGupta; and Wang and Xia, and expanded the discussion of their relation to the present framework. The revised text distinguishes Gaussian and Volterra volatility-option methods, fractional BNS long memory with $H>1/2$, and rough volatility with jumps and transforms of average forward variance. These approaches provide useful context for memory effects and nonlinear volatility payoffs. We also explain that combining them with the present finite-dimensional liquidity-feedback model would require additional state or transform constructions, which we identify as a direction for further work.

\textbf{Location:} Introduction, p. 2, lines 41--48; Calibration and nonlinear-payoff limitations, pp. 18--19.

\reviewcomment{Reviewer comment B-03}{p.3 l.6: Throughout the manuscript, there are many missing hyphens within word clusters, such as in ``liquidity-volatility''. Please double check and make sure that they are properly displayed.}

{\small\textit{Source: Report B, comment 3, p. 1.}}

\textbf{Response}\enspace We thank the reviewer for pointing out the missing hyphens. We have corrected the affected compound terms throughout the manuscript, including ``liquidity-volatility'', and replaced the problematic dash encodings in the source files. The revised PDF has been checked to confirm that these characters display correctly in both the main text and the references. This correction is consistent with the global typographical correction described in response A-Minor-04.

\textbf{Location:} Throughout the manuscript.

\reviewcomment{Reviewer comment B-04}{p.4 l.12: With Equation (4), \ensuremath{\eta} is no longer the instantaneous variance of returns, which is also modulated by the liquidity term \ensuremath{\sigma}\ensuremath{\lambda}. The authors need to adjust the interpretations accordingly.}

{\small\textit{Source: Report B, comment 4, p. 1.}}

\textbf{Response}\enspace Thank you for identifying this distinction. We now refer to $\eta$ as the base variance factor. Under the stated zero correlation between $W^1$ and $W^3$, the instantaneous variance rate of returns is $v_t=\eta_t+\sigma^2\lambda_t^2$, and instantaneous return volatility is $\sqrt{v_t}$. We have added this explanation immediately after the state equations and adjusted the interpretation of the direct liquidity channel accordingly. This makes clear how both diffusion terms contribute to return variation.

\textbf{Location:} Liquidity-adjusted price and variance dynamics, p. 4; Eq. 4, p. 4.

\reviewcomment{Reviewer comment B-05}{p.4 l.13: Parameter \ensuremath{\chi} in Equation (5) can be absorbed into parameters \textasciitilde{} \ensuremath{\gamma} and \ensuremath{\beta} in Equation (6), or alternatively, one of the latter two parameters can be normalized to 1. Doing so will improve the model on parametric parsimony.}

{\small\textit{Source: Report B, comment 5, p. 1.}}

\textbf{Response}\enspace We thank the reviewer for pointing out the redundancy in the liquidity scale. We have made the corresponding scale transformation explicit: multiplying $(\lambda,\tilde\alpha,\beta,\lambda_0)$ by $c>0$ requires adjusting $\sigma$ and $\chi$ to $\sigma/c$ and $\chi/c^2$, respectively. Under this transformation, the mean-reversion speed $\tilde\gamma$ remains unchanged. We exploit this degree of freedom by fixing $\lambda_0=0.20$ in the positive-initial-stress family studied here, while preserving the $\chi=0$ and $\sigma=0$ benchmarks. The revised manuscript describes this normalization, its scope, and the associated numerical invariance check. Removing this scale redundancy improves parametric parsimony; the identification of the remaining coefficients is treated as a separate calibration issue.

\textbf{Location:} Scale convention and model restrictions, p.~4; Appendix~B. Numerical settings, convergence and reproducibility, p.~22; numerical rescaling test, p.~23, lines 540--543.

\reviewcomment{Reviewer comment B-06}{p.4: The model involves over ten parameters in total, which makes it somewhat unparsimonious and potentially unstable when estimated or calibrated to market data. My main concern is that empirical estimation may not adequately support the level of interpretation presented in Section 4, which could limit the model's practical applicability. Though I am not requesting a full calibration study, the authors should consider adding some remarks to help substantiate the model's real-world relevance.}

{\small\textit{Source: Report B, comment 6, pp. 1, 2.}}

\textbf{Response}\enspace We thank the reviewer for the concern regarding estimation stability. We have added a discussion of calibration that distinguishes between eliminating the free liquidity scale and identifying the remaining parameters. This discussion covers several possible approaches, including the use of an observed liquidity proxy or informative restrictions, joint information across instruments, admissibility-constrained estimation, multi-start optimization, and out-of-sample checks. We also recommend comparison with simpler nested models to assess whether the additional parameters are supported by the observed data. The numerical conclusions are now explicitly confined to the illustrative scenarios studied. These additions are intended to clarify what would be required to assess the practical applicability of the model; no empirical calibration has been performed in this study.

\textbf{Location:} Calibration and nonlinear-payoff limitations, pp.~18--19.

\reviewcomment{Reviewer comment B-07}{p.4 l.24: Please state that \ensuremath{\delta}i > 0 is for all i.}

{\small\textit{Source: Report B, comment 7, p. 2.}}

\textbf{Response}\enspace Thank you for noting the missing quantifier. Assumption 2 now states $\tilde\delta_i>0$ for every $i=1,\ldots,N$. The Feller lower-bound condition takes the minimum over this same set of regimes. This makes the positivity requirement explicit for the entire state space.

\textbf{Location:} Assumption 2, p. 5; Eq. 7, p. 5.

\reviewcomment{Reviewer comment B-08}{p.5 l.15: By convention, stochastic processes should be written as either X (without the subscript t) or\{Xt\} (or (Xt), parenthesized) to avoid confusion with fixed-time random variables. Please consider making this change throughout the manuscript.}

{\small\textit{Source: Report B, comment 8, p. 2.}}

\textbf{Response}\enspace We thank the reviewer for suggesting this notational clarification. We have revised process-level descriptions to use either $X$ or an indexed family such as $(X_t)_{t\geq0}$. The same convention is applied to the variance and liquidity processes as well as to the discounted-price martingale. Time subscripts are retained in the state equations and conditional expectations where they denote a random variable at a specified time. This consistently distinguishes the two uses throughout the relevant arguments.

\textbf{Location:} Liquidity-adjusted price and variance dynamics, p.~4; Lemma~1, p.~5; Proposition~1, p.~5.

\reviewcomment{Reviewer comment B-09}{p.5 l.16: Please write ``For all t\ensuremath{\geq} 0'', and apply this language to other arguments alike.}

{\small\textit{Source: Report B, comment 9, p. 2.}}

\textbf{Response}\enspace Thank you for asking us to state the time ranges explicitly. Lemma 1 now gives the almost-sure positivity assertion for all $t\geq0$. We have also checked the related statements and specified $0\leq t\leq T$ in the martingale proposition, where $T$ is the horizon satisfying the finite-horizon sufficient conditions. The time qualifiers therefore match the hypotheses of each result.

\textbf{Location:} Lemma 1, p. 5; Proposition 1, p. 5; Assumption 3, p. 5.

\reviewcomment{Reviewer comment B-10}{p.6 l.16: Please add that z is given by Equation (15).}

{\small\textit{Source: Report B, comment 10, p. 2.}}

\textbf{Response}\enspace Thank you for noting the missing reference. We have added a direct reference to the log-price dynamics in Eq. (15) when defining the forward transform in Eq. (16). This identifies $z$ at the point where it is used and allows the definition to be followed without searching the preceding derivation.

\textbf{Location:} Eq. 16, p. 6; Eq. 15, p. 6.

\reviewcomment{Reviewer comment B-11}{p.6 l.18: Ideally, Assumption 4 should be stated in terms of model parameters and verified a posteriori --- i.e., after estimation or calibration. The authors could indicate in Section 4 whether this assumption holds in their numerical illustrations and, if not, specify the parameter ranges for which it does, along with an assessment of practical plausibility.}

{\small\textit{Source: Report B, comment 11, p. 2.}}

\textbf{Response}\enspace We thank the reviewer for asking us to make Assumption~4 more operational. We have replaced the abstract finiteness requirement in Appendix~A with an explicit finite-horizon sufficiency test and verified it for all reported production settings. This test covers the real-loading strip $[-0.1,2.1]$, including both transform stages and all real Fourier frequencies. All 147 production rows pass the Novikov and strip tests; these rows correspond to 81 distinct model-parameter sets and 115 pricing cases, including the positive-correlation scans. At the baseline, the strip envelopes are $B_*=0.733941$ and $D(T)=0.252662$. Table~8 and the saved row-level records document this coverage. We also explain that, while these theoretical tests establish admissibility for the stated scenarios, empirical plausibility and post-calibration checking remain requirements for an estimated application.

\textbf{Location:} Assumption~4, p.~6; Eq.~A.4, p.~21; Table~8, p.~23; coverage definitions, p.~23, lines 526--536.

\reviewcomment{Reviewer comment B-12}{p.10 ll.11-12: The realized volatility defined in Equation (36) is not the square root of the realized variance defined in Equation (30). Please explain why. Moreover, what does the ``zero-mean normal assumption'' mean? Does it not contradict the model implications for the return distribution?}

{\small\textit{Source: Report B, comment 12, p. 2.}}

\textbf{Response}\enspace We thank the reviewer for identifying this ambiguity in the interpretation of the contract. The quantity in Eq.~(36) is an absolute-return-based realized measure inherited from the predecessor model, and we now distinguish it explicitly from the square root of realized variance. The zero-mean normal calculation is used only to explain the normalization: if benchmark returns were independent with variance $vT/N$, the expected scaled absolute-return measure would equal $m\sqrt{v}$. This calculation imposes no normality or independence assumption on the returns generated by the proposed model. We now distinguish $\mathbb{E}[RV_O]$, $\mathbb{E}[\sqrt{RV_A}]$, and $\sqrt{\mathbb{E}[RV_A]}$, and we have removed the square-root concavity explanation as well as the claim that the chosen contract is a universal market convention. The related sensitivity discussion has been corrected consistently.

\textbf{Location:} variance-swap definition (Eq.~30), p.~8; volatility swap, p.~9.

\reviewcomment{Reviewer comment B-13}{p.11 ll.14-16: Please give more details on how the Riccati system is solved numerically.}

{\small\textit{Source: Report B, comment 13, p. 2.}}

\textbf{Response}\enspace We thank the reviewer for requesting a more detailed description of the numerical solution procedure. We now explain that the complex Riccati coefficients and the finite-state chain vector are integrated jointly using DOP853, with relative and absolute tolerances set to $10^{-10}$ and $10^{-12}$, respectively. The active-return stage terminates exactly at the monitoring-interval length, and its terminal coefficients are used to initialize the pre-monitoring stage. On the equally spaced grid, the common active stage is reused, while the earlier stage is evaluated at the required pre-monitoring horizons. The source junction is treated explicitly. The revised description further provides the Fourier refinement rule, the saved settings, and the failure-handling procedure; Appendix~B reports the solver checks against the corrected historical implementation.

\textbf{Location:} Numerical analysis, p.~10; Table~7, pp.~22--23; Table~9, p.~24.

\reviewcomment{Reviewer comment B-14}{p.11 ll.-6--7: Even in such cases, the model should ideally be amenable to robust calibration to market data, which reinforces the earlier point regarding parametric parsimony.}

{\small\textit{Source: Report B, comment 14, p. 2.}}

\textbf{Response}\enspace We thank the reviewer for again emphasizing the importance of robust calibration. We have connected the scale normalization with a more complete discussion of parameter identification. Although fixing one amplitude scale removes a redundancy, the remaining coefficients may still be weakly identified, particularly when the direct-liquidity correlation generates only a small price effect in the illustrative experiments. Accordingly, the revised manuscript recommends constrained estimation, multiple starting values, out-of-sample validation, and comparison with simpler nested models. These requirements indicate how the practical value of the additional parameters should be assessed before interpreting calibrated effects.

\textbf{Location:} Scale convention and model restrictions, p.~4; Calibration and nonlinear-payoff limitations, pp.~18--19.

\reviewcomment{Reviewer comment B-15}{p.12 ll.1-3: Please provide empirical justification for the parameter choices. In particular, the table assumes a significant price-liquidity leverage effect, which should be supported by empirical evidence, apart from the well-known price-volatility leverage effect. Moreover, \ensuremath{\epsilon} = 1.00 appears somewhat low for a variance process characterized by fast mean reversion.}

{\small\textit{Source: Report B, comment 15, p. 2.}}

\textbf{Response}\enspace We thank the reviewer for asking us to clarify the basis for the parameter choices. The baseline parameters are taken from an illustrative example in He et al., with the feedback coefficient added; they are not an empirical estimate for the present model. We have stated this explicitly and have removed the implication that $\rho_2=-0.30$ is supported by a measured price--liquidity leverage effect. The positive correlation estimated in that article under a different setting does not establish the negative choice adopted here. We have therefore added sensitivity results for this parameter under positive, negative, and zero values, which show the extent to which the illustrative conclusions depend on it. With time measured in years, $\epsilon=1$ corresponds to a one-year e-folding time, that is, a half-life of approximately 0.693 years; accordingly, we have removed the description ``fast mean reversion.'' These revisions correct the interpretation; empirical support for the parameter magnitudes would still require a calibration study.

\textbf{Location:} Table~1, p.~10; numerical analysis, p.~10; Fig.~8, p.~15.

\reviewcomment{Reviewer comment B-16}{p.18 l.-5: Again, does varying these parameter values (in particular when in the positive range) affect the integrability condition in Proposition 2?}

{\small\textit{Source: Report B, comment 16, p. 2.}}

\textbf{Response}\enspace Thank you for asking whether the correlation experiments remain admissible. We have checked the analytic Novikov and strip conditions for the separate $\rho_1$ and $\rho_2$ sweeps over $-0.75,-0.7,-0.4,0,0.4,0.7,0.75$. Every reported setting passes both sufficient tests at $T=1$, including the positive endpoints. The bounds cover the full frequency strip and both transform stages. Table 8 summarizes the checks, and the saved records give a certificate for each correlation row. We have also retained the positive-$\rho_2$ repricing check, distinguishing numerical evaluation from the analytic admissibility evidence.

\textbf{Location:} Table 8, p. 23; Eq. A.3, p. 20; Eq. A.4, p. 21.

\reviewcomment{Reviewer comment B-17}{Despite that the paper focuses on swaps, it naturally raises the question of how the framework applies to volatility options. Specifically, the semi-explicit tractability of the model stems from the linear structure of variance swaps, which allows the characteristic function of each component to be treated separately via interchange of expectation and summation. As a result, this aspect is mathematically expected and unsurprising. For nonlinear payoffs, however, the characteristic function of the realized variance would need to be treated jointly due to the complex temporal dependence structure, and the realized volatility can be interpreted as its square root. In this case, the pricing of volatility options relies either on simulation techniques (Section 3.2 of [1]) or on a modified Fourier transform-type formula (Proposition 5 in [3]). Therefore, a discussion along these lines would be a valuable addition to the paper.}

{\small\textit{Source: Report B, comment 17, p. 2.}}

\textbf{Response}\enspace We thank you for suggesting this extension and for clarifying the role of temporal dependence. We have added a discussion noting that the two contracts considered here are additive across monitoring intervals, so their expectations can be assembled from per-interval components. By contrast, a payoff based on the square root of realized variance, or an option on realized volatility, requires information on the joint path dependence across intervals. We discuss simulation, state augmentation, and an appropriate joint transform as possible approaches, and cite Section~3.2 of Horvath et al.~[1] and Proposition~5 of Wang and Xia~[3]. We also clarify that the latter result concerns average forward variance in a different rough-volatility model. Extending that approach to the discretely monitored realized variance in the present model would require further derivation. The added discussion identifies this as a substantive extension beyond the swap formulas established here.

\textbf{Location:} Calibration and nonlinear-payoff limitations, pp.~18--19.

\groupheading{Closing Remarks}

We thank the editor and the reviewers for their careful assessment and constructive suggestions. This revision has clarified the scope of the contribution, strengthened the mathematical and numerical arguments, and made the assumptions and limitations easier to assess. The point-by-point responses indicate the corresponding changes and their locations. We appreciate your consideration of the revised manuscript.

Sincerely,

Jing Fu and Ke Wang

\end{document}

Attachment Submitted filename: Response_to_Reviewers.pdf

# Editor report: Decision Letter 1

**DOI:** 10.1371/journal.pone.0355147.r003

**Authors:** Szabolcs Blazsek

7 Oct 2026

Variance and volatility swap valuation with nonlinear liquidity-feedback stochastic volatility and regime switching

PONE-D-26-35832R1

Dear Dr. Wang,

We’re pleased to inform you that your manuscript has been judged scientifically suitable for publication and will be formally accepted for publication once it meets all outstanding technical requirements.

Within one week, you’ll receive an e-mail detailing the required amendments. When these have been addressed, you’ll receive a formal acceptance letter and your manuscript will be scheduled for publication.

An invoice will be generated when your article is formally accepted. Please note, if your institution has a publishing partnership with PLOS and your article meets the relevant criteria, all or part of your publication costs will be covered. Please make sure your user information is up-to-date by logging into Editorial Manager at Editorial Manager® and clicking the ‘Update My Information' link at the top of the page. For questions related to billing, please contact billing support.

If your institution or institutions have a press office, please notify them about your upcoming paper to help maximize its impact. If they’ll be preparing press materials, please inform our press team as soon as possible -- no later than 48 hours after receiving the formal acceptance. Your manuscript will remain under strict press embargo until 2 pm Eastern Time on the date of publication. For more information, please contact onepress@plos.org.

Kind regards,

Szabolcs Blazsek

Academic Editor

PLOS One

# Editor report: Acceptance letter

**DOI:** 10.1371/journal.pone.0355147.r004

**Authors:** Szabolcs Blazsek

PONE-D-26-35832R1

PLOS One

Dear Dr. Wang,

I'm pleased to inform you that your manuscript has been deemed suitable for publication in PLOS One. Congratulations! Your manuscript is now being handed over to our production team.

At this stage, our production department will prepare your paper for publication. This includes ensuring the following:

* All references, tables, and figures are properly cited

* All relevant supporting information is included in the manuscript submission,

* There are no issues that prevent the paper from being properly typeset

You will receive further instructions from the production team, including instructions on how to review your proof when it is ready. Please keep in mind that we are working through a large volume of accepted articles, so please give us a few days to review your paper and let you know the next and final steps.

Lastly, if your institution or institutions have a press office, please let them know about your upcoming paper now to help maximize its impact. If they'll be preparing press materials, please inform our press team within the next 48 hours. Your manuscript will remain under strict press embargo until 2 pm Eastern Time on the date of publication. For more information, please contact onepress@plos.org.

You will receive an invoice from PLOS for your publication fee after your manuscript has reached the completed accept phase. If you receive an email requesting payment before acceptance or for any other service, this may be a phishing scheme. Learn how to identify phishing emails and protect your accounts at https://explore.plos.org/phishing.

If we can help with anything else, please email us at customercare@plos.org.

Thank you for submitting your work to PLOS One and supporting open access.

Kind regards,

PLOS One Editorial Office Staff

on behalf of

Dr. Szabolcs Blazsek

Academic Editor

PLOS One
